# A randomised Phase IIa trial of amine oxidase copper-containing 3 (AOC3) inhibitor BI 1467335 in adults with non-alcoholic steatohepatitis

**DOI:** 10.1038/s41467-023-42398-w

**Published:** 2023-11-06

**Authors:** Philip N. Newsome, Arun J. Sanyal, Guy Neff, Jörn M. Schattenberg, Vlad Ratziu, Judith Ertle, Jasmin Link, Alison Mackie, Corinna Schoelch, Eric Lawitz, BI 1467335 NASH Phase IIa trial team

**Affiliations:** 1 National Institute for Health Research, Birmingham Biomedical Research Centre at University Hospitals Birmingham NHS Foundation Trust, Birmingham, UK; 2 Centre for Liver & Gastrointestinal Research, Institute of Immunology and Immunotherapy, University of Birmingham, Birmingham, UK; 3 Virginia Commonwealth University, Richmond, VA USA; 4 Covenant Research, Sarasota, FL USA; 5 Metabolic Liver Research Program University Medical Center, Mainz, Germany; 6 Sorbonne Université, Institute of Cardiometabolism and Nutrition, Hospital Pitié-Salpêtrière, Paris, France; 7 Boehringer Ingelheim, Ingelheim am Rhein, Germany; 8 Boehringer Ingelheim, Biberach, Germany; 9 Texas Liver Institute, University of Texas Health, San Antonio, TX USA

**Keywords:** Clinical pharmacology, Non-alcoholic steatohepatitis

## Abstract

Non-alcoholic steatohepatitis (NASH) is a progressive, inflammatory liver disease with no approved pharmacological treatment. This Phase IIa, double-blind, placebo-controlled, multicentre trial (ClinicalTrials.gov: NCT03166735) investigated pharmacodynamics and safety of BI 1467335, an amine oxidase copper-containing 3 (AOC3) inhibitor, in adults with NASH from Europe and North America. Participants from 44 centres across the US, Germany, Spain, Belgium, the UK, Netherlands, Canada, France and Ireland were randomised (2:1:1:1:2; 27 July 2017 to 14 June 2019) to daily oral BI 1467335 1 mg (*n* = 16), 3 mg (*n* = 16), 6 mg (*n* = 17), 10 mg (*n* = 32) or placebo (*n* = 32) for 12 weeks, with follow-up to Week 16. Primary endpoint was AOC3 activity relative to baseline (%), 24 hours post-dose after 12 weeks’ treatment. Secondary biomarker endpoints included changes from baseline at Week 12 in alanine aminotransferase (ALT) and caspase-cleaved cytokeratin 18 (CK-18 caspase). Mean AOC3 activities relative to baseline at Week 12: 90.4% (placebo; *n* = 32), 26.5% (1 mg; *n* = 16), 10.4% (3 mg; *n* = 16), 5.0% (6 mg; *n* = 16), 3.3% (10 mg; *n* = 32). These changes indicated that BI 1467335 dose-dependently inhibited AOC3 activity; ≥3 mg doses achieved >80% inhibition ( < 20% activity) at Week 4. At Week 12 following doses of BI 1467335 ≥ 3 mg, ALT and CK-18 caspase decreased dose-dependently. All tested BI 1467335 doses were well tolerated, with no clinically relevant treatment-emergent safety signals. BI 1467335 strongly inhibited AOC3 in participants with NASH, with doses ≥3 mg dose-dependently reducing the levels of liver injury biomarkers, ALT and CK-18. This trial was registered with ClinicalTrials.gov (NCT03166735) and the European Union Drug Regulating Authorities Clinical Trials Database (EudraCT 2016-000499-83).

## Abstract (web summary)

The authors report data from a Phase IIa randomised, double-blind trial in patients with NASH showing that BI 1467335 strongly and dose-dependently inhibited AOC3 activity (involved in hepatic inflammation) and was well tolerated at all tested doses.

## Introduction

Non-alcoholic fatty liver disease (NAFLD) is a progressive disease with associated morbidity and mortality, and is the most common cause of liver disease, with an increasing global prevalence of 25% linked to the global epidemic of obesity and type 2 diabetes^1^. NAFLD is an independent risk factor for chronic kidney disease^2^, and depression and anxiety^3^, An estimated 7–30% of patients with NAFLD develop non-alcoholic steatohepatitis (NASH), which is a progressive and more serious subtype characterised by lobular inflammation and liver cell injury^1,4^ and has a prevalence of 5% worldwide^5^. NASH covers a wide spectrum of disease which includes progressive fibrosis that can result in cirrhosis, and its associated complications such as liver cancer or liver failure and the need for transplantation^4,6^. An estimated 20% of patients with NASH will go on to develop cirrhosis and/or hepatocellular carcinoma (HCC)^4,7^. A recent meta-analysis reported a mortality rate over a median of 6.2 years follow-up in patients with NAFLD of approximately 18%, with around 3% of deaths being specifically related to liver disease^8^. The current standard of care for NAFLD/NASH includes lifestyle interventions, such as weight loss and exercise^6,9^, which reduce inflammation and, indirectly, fibrosis^10^. The investigational treatments semaglutide, lanifibranor^11^, and resmetirom^12^ are currently in Phase III trials and also aim to reduce inflammation and fibrosis. However, thus far, there are no licensed pharmacological treatments for NASH.

Persistent inflammation resulting from liver injury drives progression to fibrosis, cirrhosis and HCC^13^. Hepatic inflammation results from the accumulation of leukocytes recruited from the circulation, which reflects a series of steps, including interaction between leukocytes and the hepatic sinusoidal endothelium, transendothelial migration and migration within hepatic tissue toward the focus of inflammation^14^. Hepatic inflammation leads not only to liver-related fibrosis but also to extrahepatic comorbidities;^15^ therefore, given the redundancy of inflammatory pathways and the upstream causes of liver injury, the ability to reduce inflammation is central to any potential intervention for NASH^13^.

A key protein in promoting the recruitment of leukocytes to liver tissue is amine oxidase copper-containing 3 (AOC3), formerly known as copper-dependent semicarbazide-sensitive amine oxidase (SSAO) or vascular adhesion protein-1^16,17^. AOC3 is constitutively expressed on human hepatic endothelium^16,17^ and, as a membrane-bound adhesion protein, is involved in inflammatory leukocyte recruitment and subsequent transmigration across the endothelium to sites of inflammation^17–20^. AOC3 is also expressed in the smooth muscle cells and endothelium of blood vessels within the human brain^21^. AOC3 enzymatic activity also catalyses the oxidative deamination of primary amines to produce hydrogen peroxide, ammonium and aldehyde^17^, which are associated with oxidative stress.

Several lines of evidence, including its enzymatic activity and role in leukocyte recruitment, implicate AOC3 in the pathophysiology of NASH^16,22^. A soluble form of AOC3 (sAOC3), also found in human serum, is derived from a cleavage product of the membrane-bound protein and has amine oxidase activity. Sera from patients with chronic and inflammatory liver diseases (including alcohol-related liver disease, primary biliary cholangitis, cryptogenic cirrhosis and HCC) show elevated levels of sAOC3 (thought to be derived mainly from the liver) and AOC3 enzymatic activity^23,24^. Furthermore, hepatic expression of membrane-bound AOC3 and serum levels of sAOC3 are increased in patients with NAFLD, and levels of sAOC3 correlate with fibrosis stage and are predictive of progression to NASH^16^. Lastly, animal models suggest that AOC3 promotes progression of steatohepatitis and that AOC3 enzymatic activity is crucial for the establishment of fibrosis^16^. AOC3 inhibition, using anti-AOC3 antibodies, has been shown to reduce inflammatory cell recruitment to the liver in murine hepatic injury models and in vitro cultured active hepatic stellate cells from liver tissue of patients with NASH cirrhosis^16^. AOC3 inhibition also attenuated fibrosis in a murine NAFLD/NASH model (mice fed a methionine choline-deficient diet)^16^. BI 1467335 (Pharmaxis PXS-4728A) is an oral, small-molecule inhibitor of AOC3^25^, which we hypothesised may reduce oxidative stress and hepatic inflammation in steatohepatitis, and halt or reverse the progression of fibrosis in patients with NASH^16,22,25^.

Herein, we report results of a randomised, placebo-controlled Phase IIa trial that assessed the safety, tolerability and pharmacodynamics of different doses of orally administered BI 1467335 during a 12-week treatment period, compared with placebo, in patients with clinical evidence of NASH.

## Results

### Trial patients and compliance

Between 27 July 2017 and 14 June 2019, 114 patients were enrolled and randomly assigned to one of five treatment groups. The treated population included 113 patients who received at least one dose of daily oral BI 1467335 1 mg (*n* = 16), 3 mg (*n* = 16), 6 mg (*n* = 17), 10 mg (*n* = 32) or placebo (*n* = 32). In total, 16 patients had at least one important protocol deviation (*n* = 11, treatment duration was too short; *n* = 5, failure to meet entry criteria; *n* = 4, non-compliance to trial medication; *n* = 2, prohibited medication use; *n* = 2, missing on-treatment biomarker value; *n* = 1, no trial medication taken) resulting in a per-protocol population of 98 patients. The safety population included 113 patients. The full analysis set, which was used for the sensitivity analysis of the primary endpoint, included 112 patients. Patient characteristics and demographics were similar between groups. There was no relevant change over time in body mass index (BMI) or any other physical parameters over the course of the trial. Almost half of the 113 patients were male (48.7%), most patients (96.5%) were white and the mean age (standard deviation [SD]) was 51.1 years (12.5) (Table 1). Baseline demographics, disease characteristics and key biomarkers are shown in Table 1.

**Table 1:** Baseline demographics, disease characteristics and biomarkers (TS)

	Placebo (*n* = 32)	BI 1467335				Total overall (*N* = 113)
		1 mg (*n* = 16)	3 mg (*n* = 16)	6 mg (*n* = 17)	10 mg (*n* = 32)	
Sex, *n* (%)						
Female	13 (40.6)	10 (62.5)	8 (50.0)	9 (52.9)	18 (56.3)	58 (51.3)
Male	19 (59.4)	6 (37.5)	8 (50.0)	8 (47.1)	14 (43.8)	55 (48.7)
Mean age ± SD, years	51.8 ± 12.3	52.6 ± 13.3	53.9 ± 11.5	48.2 ± 10.1	49.8 ± 14.0	51.1 ± 12.5
Race, *n* (%)						
White	30 (93.8)	14 (87.5)	16 (100)	17 (100)	32 (100)	109 (96.5)
Asian	1 (3.1)	1 (6.3)	0	0	0	2 (1.8)
Native Hawaiian or other Pacific Islander	1 (3.1)	1 (6.3)	0	0	0	2 (1.8)
Weight, mean ± SD, kg	95.1 ± 18.8	96.7 ± 21.7	89.7 ± 12.6	91.7 ± 16.9	90.1 ± 18.2	92.7 ± 17.9
BMI, mean ± SD, kg/m^2^	33.1 ± 5.1	34.2 ± 5.4	31.7 ± 4.2	31.5 ± 4.2	32.3 ± 4.9	32.6 ± 4.8
Overweight, *n* (%)	27 (84.4)	15 (93.8)	13 (81.3)	15 (88.2)	26 (81.3)	96 (85.0)
T2DM, *n* (%)	12 (37.5)	7 (43.8)	5 (31.3)	5 (29.4)	12 (37.5)	41 (36.3)
Arterial hypertension, *n* (%)	17 (53.1)	10 (62.5)	10 (62.5)	6 (35.3)	17 (53.1)	60 (53.1)
Hyperlipidaemia, *n* (%)	21 (65.6)	7 (43.8)	12 (75.0)	8 (47.1)	20 (62.5)	70 (61.9)
Depression, *n* (%)	3 (9.4)	4 (25.0)	3 (18.8)	3 (17.6)	3 (9.4)	16 (14.2)
AOC3, mean ± SD, μg/L	471.4 ± 165.7	537.7 ± 204.4^a^	498.0 ± 141.0	527.3 ± 142.4	516.2 ± 144.1^b^	505.0 ± 157.5^c^
ALT, mean ± SD, U/L	78.0 ± 28.8	89.6 ± 24.7	82.3 ± 25.5	87.2 ± 33.6	88.7 ± 51.4	84.7 ± 36.2
AST, mean ± SD, U/L	50.1 ± 18.5	61.8 ± 31.5	66.1 ± 38.7	54.4 ± 18.8	60.2 ± 38.5	57.5 ± 30.4
AP, mean ± SD, U/L	76.2 ± 21.8	86.1 ± 25.1	90.1 ± 25.3	100.3 ± 43.4	88.4 ± 33.9	86.6 ± 30.8
GGT, mean ± SD, U/L	82.2 ± 51.3	138.3 ± 319.1	170.4 ± 140.0	108.4 ± 132.9	128.4 ± 136.3	119.7 ± 159.7
CK-18 caspase, mean ± SD, U/L	574.0 ± 556.1	726.1 ± 386.0	866.5 ± 916.4	593.0 ± 477.6	677.7 ± 670.1	669.2 ± 620.0
CK-18 total, mean ± SD, U/L	1142.3 ± 725.3	1400.8 ± 592.0^d^	1594.6 ± 1060.2	1066.9 ± 480.2	1242.6 ± 785.2	1258.7 ± 759.6^e^

*ALT* alanine aminotransferase, *AOC3* amine oxidase copper-containing 3, *AP* alkaline phosphatase, *AST* aspartate aminotransferase, *BMI* body mass index, *CK-18 caspase* caspase-cleaved cytokeratin 18, *CK-18 total* total cytokeratin 18, *GGT* gamma-glutamyl transferase, *T2DM* type 2 diabetes mellitus, *TS* treated set, *ULN* upper limit of normal.

^a^*n* = 14

^b^*n* = 31

^c^*n* = 110

^d^*n* = 15

^e^*n* = 112

**Fig. 1:**
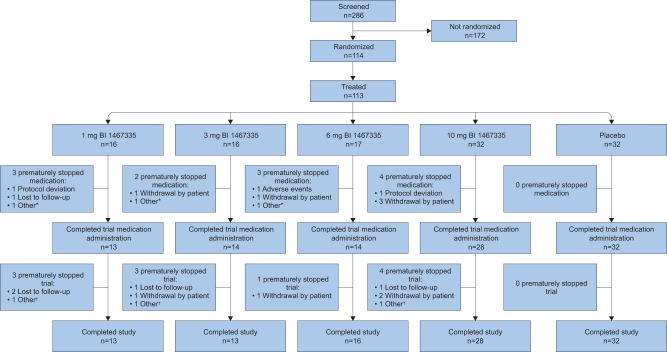
CONSORT flow diagram. *Other reasons for the premature discontinuation of trial medication were the patient started prohibited concomitant medication (*n* = 2) and sponsor’s decision (*n* = 1). ^†^Other reasons for trial discontinuation were that follow-up was not completed as planned, whether completed early or by telephone (*n* = 3).

Of the 113 patients treated during the trial, 12 patients (10.6%) discontinued trial medication (all BI 1467335); one discontinuation in the 6 mg arm was due to a treatment-emergent adverse event (TEAE): moderate headache (other reasons for discontinuation are given in Fig. 1).

Good compliance to trial medication was documented in 15 patients (94%) from the 1 mg arm and in all patients from the other arms. Patient disposition is shown in Fig. 1.

### Primary outcome

**Fig. 2:**
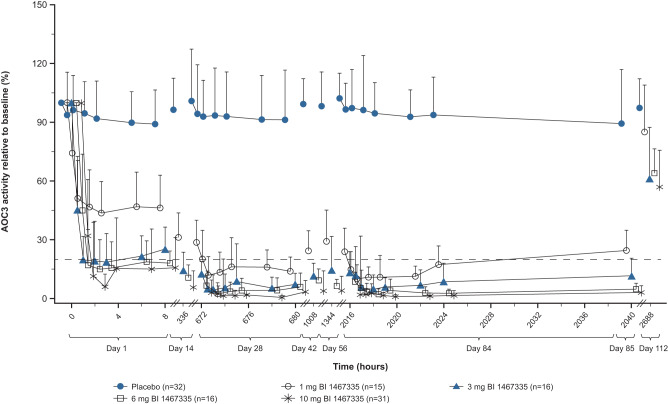
AOC3 activity relative to baseline. Mean AOC3 activity relative to baseline versus time curves after once daily administration of 1 mg, 3 mg, 6 mg or 10 mg BI 1467335 or placebo for up to 85 days (whole profile days at Day 1, 28 and 84; FAS). Error bars show standard deviation from the mean. Source data are provided as a Source Data file. Dotted line indicates 20% residual AOC3 activity relative to baseline. AOC3 amine oxidase copper-containing 3, FAS full analysis set.

BI 1467335 resulted in dose-dependent inhibition of plasma AOC3 activity, with >80% reductions from baseline seen by Week 4 with doses of ≥3 mg. Relative to baseline values, mean AOC3 activity at Week 12 (24 hours post dose) was 26.5% in the BI 1467335 1 mg dose group, (indicating 73.5% inhibition), 10.4% in the 3 mg group (indicating 89.6% inhibition), 5.0% in the 6 mg group (95.0% inhibition) and 3.3% in the 10 mg group (96.7% inhibition); mean plasma AOC3 activity in placebo recipients was 90.4%, indicating that no substantial change was observed with placebo and thus meeting the primary endpoint (Supplementary Table 1). The duration of inhibition also increased with dose: BI 1467335 treatment resulted in >80% inhibition of AOC3 for the entire 24-hour dosing interval, following the first 10 mg dose and from around Week 4 of treatment for doses of 3 mg and 6 mg, whereas for the 1 mg dose, the duration of >80% AOC3 inhibition was for approximately half a day (Fig. 2). Statistical analysis of the primary endpoint predicted that a 90% reduction of baseline AOC3 activity at Week 12 could be achieved with a daily dose of BI 1467335 3.45 mg and within a post hoc analyses an 80% reduction of baseline AOC3 activity at Week 12 could be reached with a daily dose of 1.58 mg. Plasma AOC3 concentrations fluctuated but remained similar to baseline levels throughout the trial in the placebo and across all BI 1467335 groups (Supplementary Table 2). Therefore, BI 1467335 inhibited AOC3 activity without altering the plasma concentration of AOC3.

### Secondary outcomes

**Fig. 3:**
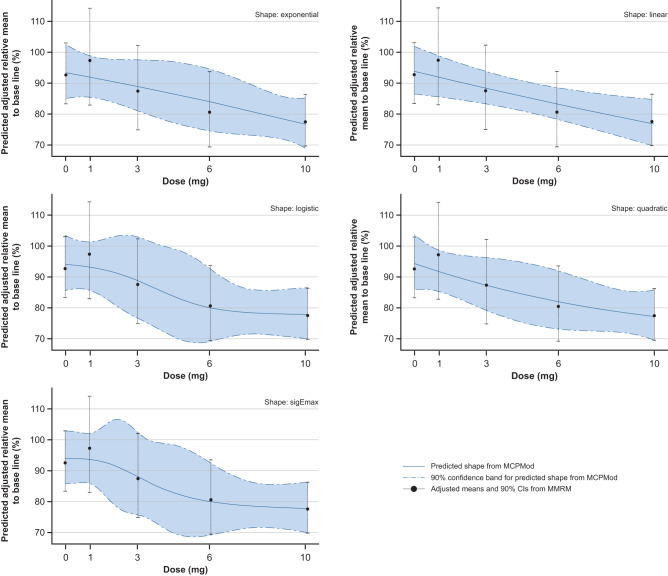
Significant dose–response relationship of ALT relative to baseline at Week 12 (PPS). Error bars show MMRM-generated adjusted mean (least-square means) ALT and two-sided 90% CI estimates based on the t-distribution (detailed in Table 2) for each dose relative to baseline at Week 12. Five different candidate dose–response patterns (blue trend lines), generated using the multiple contrast test MCPMod, show the predicted shape of the dose–response relationship. The figure shows all models that had a significant test result (i.e. the null hypothesis of a flat dose-response curve was rejected with alpha 0.05, one-sided). *P*-values are provided thereafter for each of these models: exponential, 0.0321; linear, 0.0256; logistic, 0.0216; quadratic, 0.0254; sigEmax, 0.0212. Source data are provided as a Source Data file. ALT alanine aminotransferase, MCPMod Multiple Comparison Procedure—Modelling, MMRM mixed effects model for repeated measurements, PPS per-protocol set.

**Fig. 4:**
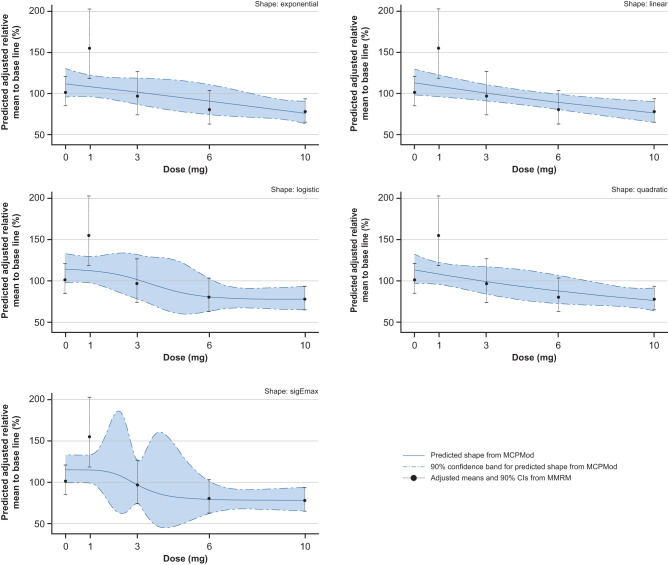
Significant dose–response relationship of CK-18 caspase relative to baseline at Week 12 (PPS). Error bars show MMRM-generated adjusted mean (least-square means) CK-18 caspase and two-sided 90% CI estimates based on the t-distribution (detailed in Table 3) for each dose relative to baseline at Week 12. Five candidate dose–response patterns (blue shapes), generated using the multiple contrast test MCPMod, show the predicted shape of the dose–response relationship. The figure shows all models that had a significant test result (i.e. the null hypothesis of a flat dose-response curve was rejected with alpha 0.05, one-sided). *P*-values are provided thereafter for each of these models: exponential, 0.0112; linear, 0.0093; logistic, 0.0047; quadratic, 0.0098; sigEmax, 0.0042. Source data are provided as a Source Data file. CK-18 caspase caspase-cleaved cytokeratin 18, MCPMod Multiple Comparison Procedure—Modelling, MMRM mixed effects model for repeated measurements, PPS per-protocol set.

At Week 12, alanine aminotransferase (ALT) and caspase-cleaved cytokeratin 18 (CK-18 [M30]) showed a significant (*p* < 0.05) non-flat dose–response relationship with BI 1467335 treatment (exponential, linear, logistic, quadratic and sigma Emax shapes are shown in Figs. 3 and 4, respectively). Dose–response relationships were not significant for aspartate aminotransferase (AST), alkaline phosphatase (AP), gamma-glutamyl transferase (GGT) and total cytokeratin 18 (CK-18 total [M65]) (Supplementary Fig. 2).

After 12 weeks of treatment, the 3 mg, 6 mg and 10 mg doses of BI 1467335 resulted in an adjusted mean placebo-corrected relative change in ALT of −5.2% (90% CI: −21.7, 11.4), −12.0% (90% CI: −27.4, 3.3) and −15.1% (90% CI: −27.8, −2.3), respectively (Table 2). Doses of 3 mg, 6 mg and 10 mg BI 1467335 also resulted in a dose-dependent, adjusted mean placebo-corrected relative change in CK-18 caspase of −4.5% (90% CI: −35.6, 26.6), −20.8% (90% CI: −47.5, 5.8) and −23.3% (90% CI: −45.8, −0.8) (Table 3). The decrease in AST at Week 12 following treatment with 3 mg, 6 mg and 10 mg BI 1467335 showed no clear dose dependency (Supplementary Table 3). No clear change in ALT and AST was observed with the 1 mg BI 1467335 dose (although placebo-corrected values indicated increases of a similar magnitude to the decreases observed with higher doses), but an increase in CK-18 caspase was observed with the 1 mg BI 1467335 dose (confirmed by placebo-corrected values).

**Table 2:** ALT relative to baseline at Week 12 (PPS)

		Relative to baseline, %				Comparison vs placebo, %		
Treatment group	*n*	Adjusted mean		SE	90% CI	Adjusted mean	SE	90% CI
Placebo	28	92.7		106.6	83.4, 103.0	–	–	–
BI 1467335 1 mg	12	97.3		110.1	82.9, 114.2	4.7	11.0	−13.5, 22.8
BI 1467335 3 mg	13	87.5		109.8	74.9, 102.2	−5.2	10.1	−21.7, 11.4
BI 1467335 6 mg	14	80.6		109.4	69.4, 93.6	−12.0	9.3	−27.4, 3.3
BI 1467335 10 mg	27	77.6		106.7	69.7, 86.4	−15.1	7.8	−27.8, −2.3

*ALT* alanine aminotransferase, *PPS* per-protocol set.

**Table 3:** CK-18 caspase relative to baseline at Week 12 (PPS)

		Relative to baseline, %				Comparison vs placebo, %		
Treatment group	*n*	Adjusted mean		SE	90% CI	Adjusted mean	SE	90% CI
Placebo	29	101.4		111.2	85.0, 120.8	–	–	–
BI 1467335 1 mg	12	155.0		117.6	118.4, 203.0	53.7	27.2	9.0, 98.4
BI 14673353 mg	12	96.9		117.5	74.1, 126.7	−4.5	18.9	−35.6, 26.6
BI 1467335 6 mg	14	80.5		116.3	62.6, 103.5	−20.8	16.2	−47.5, 5.8
BI 1467335 10 mg	26	78.1		111.6	65.1, 93.7	−23.3	13.7	−45.8, −0.8

*CK-18 caspase* caspase-cleaved cytokeratin 18, *PPS* per-protocol set.

Changes in other biomarkers were inconsistent (GGT (Supplementary Table 4) and CK-18 total (Supplementary Table 5)) or absent (AP (Supplementary Table 6)). Placebo treatment was not associated with substantial changes in these biomarkers. The dynamic change over time relative to baseline and the concentrations over time of ALT, AST, AP, GGT, CK-18 caspase and CK-18 total are shown in Supplementary Fig. 3 and Supplementary Tables 7 to 12, respectively. At follow-up, levels of these secondary biomarkers were broadly similar in magnitude to baseline levels. A post hoc analysis performed on the pro-peptide of type lll collagen (Pro-C3) in patients with a baseline Pro-C3 value > 15 ng/mL indicated a positive but weak relationship between the change from baseline in ALT or AST and the change from baseline in Pro-C3 (Supplementary Figs. 4 and 5). This weak positive relationship seems to become more pronounced the higher the dose and the longer the treatment. However, no dose-dependent change from baseline in Pro-C3 over time was identified in the full analysis set (Supplementary Fig. 6). In general, the descriptive statistics on metabolic (fasting plasma glucose, insulin, cholesterol and triglyceride) and inflammatory markers (interferon γ and interleukins 1β, 6 and 8) and other markers and scores reflecting fibrosis status (aspartate aminotransferase to platelet ratio index [APRI], Fibrosis-4 score [Fib-4 score], enhanced liver fibrosis [ELF] score, NAFLD fibrosis score and Pro-C3) did not show relevant trends over time or differences for any BI 1467335 dose (Supplementary Tables 13–25).

### Safety

The incidence of TEAEs was similar in the 81 patients receiving BI 1467335 (65.6–76.5%) and the 32 patients receiving placebo (62.5%) (Table 4). There was no dose-related increase in TEAEs. Most patients had TEAEs of grade ≤2. One patient receiving placebo and three patients receiving BI 1467335 had at least one grade ≥3 TEAE; all patients recovered. Two of these events were considered related to the trial medication (GGT increased in the 3 mg group and AST increased in the 10 mg group: both grade 3). The most frequently reported TEAE in >10% of the 32 patients in the placebo group was headache (12.5%). Of the 81 patients in the BI 1467335 groups, the most frequently reported TEAEs in >10% participants were nasopharyngitis (13.6%), nausea (12.3%) and headache (11.1%).

**Table 4:** Summary of treatment-emergent adverse events (TS)

Treatment-emergent adverse event, *n* (%)	Placebo (*n* = 32)	BI 1467335			
		1 mg (*n* = 16)	3 mg (*n* = 16)	6 mg (*n* = 17)	10 mg (*n* = 32)
Any TEAE	20 (62.5)	12 (75.0)	12 (75.0)	13 (76.5)	21 (65.6)
Severe TEAEs (CTCAE grade 3 or 4)	1 (3.1)	0	2 (12.5)	0	1 (3.1)
Investigator-defined drug-related TEAEs	8 (25.0)	5 (31.3)	2 (12.5)	2 (11.8)	8 (25.0)
TEAEs leading to drug discontinuation	0	0	0	1 (5.9)	0
TEAESIs^a^	1 (3.1)	0	0	0	1 (3.1)
Serious TEAEs^b^	1 (3.1)	1 (6.3)	1 (6.3)	0	0
Common TEAEs^c^					
Diarrhoea	3 (9.4)	1 (6.3)	1 (6.3)	2 (11.8)	3 (9.4)
Nausea	1 (3.1)	4 (25.0)	1 (6.3)	0	5 (15.6)
Vomiting	0	2 (12.5)	0	0	0
Fatigue	3 (9.4)	0	1 (6.3)	0	5 (15.6)
Influenza	0	2 (12.5)	0	0	0
Nasopharyngitis	3 (9.4)	3 (18.8)	2 (12.5)	1 (5.9)	5 (15.6)
Urinary tract infection	2 (6.3)	2 (12.5)	0	0	1 (3.1)
Back pain	1 (3.1)	2 (12.5)	1 (6.3)	0	1 (3.1)
Neck pain	0	0	0	2 (11.8)	0
Dizziness	0	1 (6.3)	2 (12.5)	0	3 (9.4)
Headache	4 (12.5)	3 (18.8)	0	4 (23.5)	2 (6.3)

TEAEs were coded using MedDRA v22.0. The severity of TEAEs was graded according to CTCAE v4.03.

*AE* adverse event, *ALT* alanine aminotransferase, *AST* aspartate aminotransferase, *CTCAE* Common Terminology Criteria for Adverse Events, *MedDRA* Medical Dictionary for Drug Regulatory Activities, *TEAE* treatment-emergent adverse event, *TEAESI* treatment-emergent adverse event of special interest, *TS* treated set, *ULN* upper limit of normal.

^a^TEAESIs were liver injury events (ALT and/or AST 5–8 × baseline or >300 U/l in patients with ALT and/or AST > ULN at baseline; AST 3–8 × ULN in patients with normal AST at baseline).

^b^A serious TEAE was defined as any AE which resulted in death, was immediately life-threatening, resulted in persistent or significant disability/incapacity, required or prolonged patient hospitalisation, was a congenital anomaly/birth defect, or was to be deemed serious for any other reason.

^c^Common TEAEs were reported in ≥10% of patients in any treatment group.

The incidence of these TEAEs did not increase with increasing doses of BI 1467335. A total of six serious TEAEs were reported in three patients (one in the placebo group [sinusitis and nasal septum deviation requiring hospitalisation], one in the 1 mg group [mild pancreatitis, not considered a serious TEAE by the investigator but categorised as such per the sponsor’s request] and one in the 3 mg group [H1N1 influenza, acute respiratory failure and chronic obstructive pulmonary disease requiring hospitalisation]); none were related to treatment and all three patients recovered. During the trial, one patient discontinued treatment with 6 mg BI 1467335 due to a moderate TEAE (headache) that was considered related to the trial medication, but the patient completed the trial.

Fewer investigator-defined drug-related TEAEs were observed in the 81 patients receiving BI 1467335 (21.0% all dose groups [1 mg, *n* = 16; 3 mg, *n* = 16; 6 mg, *n* = 17 and 10 mg, *n* = 32]) than in the 32 patients receiving placebo (25.0%). Two patients experienced liver injury events (TEAE of special interest [TEAESI]) during the trial. One patient in the placebo group had a mild AST increase (Day 140–152) that did not require treatment and a concurrent ALT increase (not considered a TEAESI) and subsequently recovered. ALT and AST levels were still elevated at follow-up. One patient in the BI 1467335 10 mg group had an AST increase (Day 15–43) of severe intensity that was not treated and was considered as possibly related to trial medication. The patient recovered. The same patient experienced an AST increase and blood bilirubin increase, starting on Day 130, both of mild intensity and not requiring treatment. ALT, AST and bilirubin levels were still elevated at follow-up. Neither patient was a Hy’s Law case since bilirubin was normal throughout treatment in the placebo case and elevation of liver enzymes was explained by Gilbert’s syndrome in the BI 1467335 10 mg case. There were no deaths in the trial.

## Discussion

At Week 12 in this Phase IIa trial, BI 1467335 strongly inhibited plasma AOC3 activity in a dose-dependent manner, along with showing a reduction in markers of liver injury. There was >80% inhibition of AOC3 activity for the entire 24-hour dosing interval from Week 4 onwards of treatment for doses ≥3 mg indicating adequate target engagement. The magnitude of AOC3 inhibition was similar to that seen in previous studies in healthy volunteers [Mackie et al., manuscript in preparation]. While strong inhibition of plasma AOC3 activity was seen earlier than Week 4 with the 6 mg and 10 mg doses of BI 1467335 in healthy volunteers, this was most likely due to a difference in the timing of sample collection rather than an actual difference in these populations.

At Week 12 in this trial, the liver injury biomarkers ALT and CK-18 caspase decreased in response to treatment with 3–10 mg BI 1467335 in a dose-dependent manner, with a similar trend being observed for AST. ALT level is a marker of liver injury and elevated serum ALT indicates hepatic inflammation and liver injury in patients with various liver diseases^26^. CK-18 caspase is released from hepatocytes during apoptosis and is associated with inflammation and fibrosis in various chronic liver diseases, including NASH^27–29^. A positive but weak relationship was observed between the change from baseline in Pro-C3 (another marker of fibrosis) and the changes from baseline in ALT or AST. There was no clear dose effect, however this weak relationship became more pronounced with higher doses and longer treatment. No clear dose-dependent trend over time was seen for further metabolic, inflammatory and fibrosis biomarkers that, together with the increases in ALT, AST and CK-18 caspase in the BI 1467335 1 mg group is likely to be due to the presumed sub-therapeutic dose, large variability between patients, the presence of outliers and the small sample size in each group. Nevertheless, the dose-dependent reduction in the liver injury biomarkers ALT and CK-18 caspase in the absence of changes in BMI observed in the 3–10 mg BI 1467335 groups provides further support for adequate target engagement and proof-of-mechanism in patients with NASH.

The administration of up to 10 mg BI 1467335 once daily for 12 weeks did not identify any unexpected safety signals and BI 1467335 was well tolerated by the patients with NASH enrolled in this trial. Only one patient in the 6 mg BI 1467335 treatment group discontinued treatment due to a moderate TEAE (headache) and both patients who experienced a liver injury event (TEAESI) recovered and completed the trial. The safety profile of BI 1467335 in the present trial was consistent with that seen in Phase I trials in healthy volunteers (Mackie, et al. manuscript in preparation). Further development of BI 1467335 was stopped due to the risk of drug interactions of the compound with monoamine oxidase (MAO)-B in NASH patients identified in another Phase I trial^30,31^. Preclinical data suggest that BI 1467335 and RTU-1096 (another AOC3 inhibitor) have an IC_50_ against AOC3 and MAO-B in the nanomolar and micromolar range, respectively indicating a high degree of specificity for AOC3^32,33^. Whether the MAO-B inhibition observed in humans^31^ is a class effect or specific to BI 1467335 is, as yet, unknown. Nevertheless, liver injury biomarkers were reduced and BI 1467335 was well tolerated in this Phase IIa trial. As such, AOC3 remains an attractive target for reducing hepatic inflammation, with the potential for subsequent beneficial effects on fibrosis in patients with NASH.

In conclusion, BI 1467335 strongly inhibited AOC3, was well tolerated at all tested doses, and showed dose-dependent reductions in serum ALT and CK-18 caspase, resulting in >10% placebo-corrected reduction from baseline for ≥6 mg doses.

## Methods

### Trial design and participants

All patients provided written informed consent before entering the trial and this trial was conducted in accordance with Good Clinical Practice, the ethical principles laid down in the Declaration of Helsinki and applicable regulatory requirements. The full study protocol is available as a supplementary file. Five global protocol amendments were implemented to the study protocol with Amendments 3, 4 and 5 implemented following the approval of version 3.0 of the study protocol. Details and rationale for the implemented protocol amendments are provided in the supplementary materials. The Institutional Review Boards and Independent Ethics Committees that approved each version of the study protocol are listed in Supplementary Table 26.

This multicentre, parallel-group, randomised, double-blind, placebo-controlled Phase IIa trial (ClinicalTrials.gov: NCT03166735) was conducted at 44 centres across the US, Germany, Spain, Belgium, the UK, Netherlands, Canada, France and Ireland between 27 July 2017 and 14 June 2019. Eligible patients were adults (aged 18–75 years), with either clinical evidence of NASH defined as histological evidence no more than 3 years prior to screening or clinical imaging suggestive of NASH (evidence of hepatic steatosis by magnetic resonance imaging of proton density fat fraction or ultrasound and evidence of liver fibrosis defined by stiffness >3.64 kPa with magnetic resonance elastography or >7.2 kPa with transient elastography) no more than 3 years prior to screening or within the screening phase. Patients were also required to have an ALT level >1.5 to ≤5.0 × the upper limit of normal (ULN) or historic ALT > 1.25 × ULN within 1 week to 3 months prior to screening and two consecutive ALT > 1.5 × ULN measurements at least one week apart during the screening period to be included in the trial.

Patients were excluded if they had cirrhosis; other causes of chronic liver disease; a current or recent (within 5 years) history of significant alcohol consumption (>210 g or >140 g per week in men or women, respectively); a bilirubin level >ULN; or glycosylated haemoglobin ≥9.5% or a change in body weight ≥5% in the 3 months prior to screening. A prior risk evaluation using a mechanistic static model suggested that BI 1467335 concentrations in man following 10 mg once daily could potentially inhibit MAO-B irreversibly. Due to this potential risk, concomitant medications, such as antidepressants, MAO inhibitors and serotonergic compounds, were prohibited.

### Randomisation and blinding

Eligible patients were randomised using interactive response technology (IRT) 2:1:1:1:2 (block size 7) to receive placebo or BI 1467335 1 mg, 3 mg, 6 mg or 10 mg orally once daily for 12 weeks. Use of IRT provided a depersonalised patient identification code and ensured confidentiality of patient data; the randomisation list was generated using a validated system, which involved a pseudo-random number generator so that the resulting treatment was both reproducible and non-predictable. All trial participants, investigators and site staff were blinded to the assigned treatment.

### Procedures

All patients received 5 film-coated tablets daily to be taken orally before breakfast, supplied as placebo or BI 1467335 in 1 mg and 5 mg dose strengths. Each treatment group received a combination of placebo and BI 1467335 tablets corresponding to the appropriate dose (e.g. the 6 mg dose group received 1 × 5 mg BI 1467335, 1 × 1 mg BI 1467335, 3 × placebo). All treatments were double blind. Assessments of sAOC3 activity and concentration, ALT, AST, AP, GGT, CK-18 caspase and CK-18 total, and other exploratory biomarkers were carried out at each study visit. Study visits took place every 2 weeks for the first 8 weeks, at Week 12 and at a follow-up visit 4 weeks after trial drug termination (Week 16). TEAEs were recorded at each study visit, at follow-up and by telephone call on Days 27 and 83. TEAESIs were liver injury events (ALT and/or AST 5–8 × baseline or >300 U/L in patients with ALT and/or AST > ULN at baseline; AST 3–8 × ULN in patients with normal AST at baseline) and trial-specific procedures for the removal of individual patients were defined in cases of increased liver enzymes (AST, ALT and total bilirubin) after randomisations^34^ and are shown in Fig. S1. In suspected cases of elevated liver enzymes, treatment was temporarily interrupted until other potential causes of liver injury were excluded and values were confirmed by retesting within 48 hours. If values were confirmed, trial medication remained interrupted and the patient was monitored weekly until resolution or stabilisation. Full details of interruption, restarting and stopping criteria are provided in supplementary materials.

### Outcomes

The primary endpoint was plasma AOC3 activity relative to baseline measured as a percentage, 24 hours post dose after 12 weeks of treatment. AOC3 activity was measured via a quasi-quantitative, one-step fluorometric activity assay (Amplex® Red Monoamine Oxidase Assay Kit). In this assay, hydrogen peroxide, produced during oxidation of benzylamine by AOC3, was used as a proxy for quantification of AOC3 activity. Hydrogen peroxide oxidised Amplex Red to its fluorescent analogue, resorufin, allowing for colorimetric analysis of AOC3 activity. The number and percentage of patients with drug-related adverse events (AEs) was a secondary endpoint in this trial. Safety and tolerability were further assessed based on the general occurrence of TEAEs, safety laboratory parameters, physical examination, vital sign measurements and a 12-lead electrocardiogram. The intensity of AEs was classified and recorded according to the Common Terminology Criteria for Adverse Events (CTCAE) v4.03. Secondary biomarker endpoints were relative changes from baseline in ALT, AST, AP, GGT, CK-18 caspase, and CK-18 total at Week 12. Further biomarker endpoints included markers of metabolism (e.g. fasting plasma glucose, insulin and lipids), inflammation (e.g. interleukins 1β, 6 and 8, and interferon γ) and markers and scores reflecting fibrosis status (e.g. APRI, Fib-4 score, ELF score, NAFLD fibrosis score and Pro-C3).

### Sample size calculation

The sample size calculation was based on the primary endpoint, as well as the ALT change from baseline; the latter being the biomarker with the least favourable ratio of anticipated effect size to variance. A sample size of 108 patients with an allocation ratio of 2:1:1:1:2 for placebo, BI 1467335 1 mg, 3 mg, 6 mg or 10 mg, with an assumed 10% discontinuation rate and assumed maximum change of 30%, was anticipated to provide sufficient precision for the primary endpoint evaluation as well as an 84.8% probability of detecting a 20% relative change from baseline in ALT at Week 12. This calculation was based on 1000 simulations using Multiple Comparison Procedure—Modelling (MCPMod)^35^, assuming a baseline ALT of 80 U/L with an SD of 40, a placebo effect of −10 U/L and a treatment effect of −34 U/L at Week 12, with the null hypothesis of no dose relationship rejected at one-sided alpha 0.05.

### Missing data

Missing AOC3 activity data were subject to imputation of missing values. Prior to imputation, the pattern of missing data was explored using a blinded dataset. If the pattern was monotone, a regression model was used for the imputation. Otherwise, a Markov chain Monte Carlo step was applied to create monotone data in multiple datasets prior to using a regression method. A decreasing 3-parameter curve model was then applied. For the secondary biomarkers, the pattern of missing values was assumed to be random, and directly handled within the mixed effects model for repeated measurements (MMRM) based on the likelihood method. If one of the three electrocardiogram cardiac cycles was missing, the arithmetic mean for this single electrocardiogram was computed using the available cardiac cycles. Imputation was not performed for safety endpoints, further exploratory biomarker and pharmacogenomic endpoints or other variables. Only patients without missing baseline data and with at least one non-missing post-baseline, on-treatment biomarker measurement for any primary, secondary or further biomarker endpoint were included in the full analysis set and the per-protocol set.

### Statistical analysis

Statistical analyses were performed using SAS version 9.4 (SAS Inc., Cary, NC, USA). Primary and secondary non-safety endpoints were evaluated using the per-protocol population of all randomised patients, excluding patients with no baseline and/or on-treatment value and important protocol deviations leading to exclusion. Safety analyses used all randomised patients who received trial treatment. The dose–response relationship of the primary endpoint was analysed using a nonlinear regression model (decreasing Emax curve) applied to the AOC3 activity at Week 12. The fitted regression model was used to derive the smallest dose where the mean plasma AOC3 activity curve dropped below 10%. For the secondary biomarker endpoints, the MMRM was used to generate adjusted mean and 90% CI estimates for the treatment effects at Week 12. These estimates, together with the corresponding covariance matrix, were used to analyse the dose–response relationship by examining the fit of eight shapes modelled using MCPMod^36,37^, allowing for simultaneous evaluation of different potential dose–response patterns, while protecting the overall probability of type I error (one-sided alpha of 0.05). A test for non-flat dose–response relationship was first performed; if a relationship could be shown, all significant models from a set of candidate models were selected and fitted to the data as a second step.

### Reporting summary

Further information on research design is available in the Nature Portfolio Reporting Summary linked to this article.

### Supplementary information

Supplementary Information

Peer Review File

Reporting Summary

### Source data

Source Data

## Data Availability

To ensure independent interpretation of clinical study results and enable authors to fulfil their role and obligations under the ICMJE criteria, Boehringer Ingelheim grants all authors who are not working for Boehringer Ingelheim access to relevant clinical study data pertinent to the development of the publication. In adherence with the Boehringer Ingelheim Policy on Transparency and Publication of Clinical Study Data, scientific and medical researchers can request access to clinical study data after publication of the primary manuscript and this request will be evaluated within 3 months. The data access criteria can be found on the Boehringer Ingelheim’s member page: https://vivli.org/ourmember/boehringer-ingelheim/. Researchers should use the https://vivli.org/members/enquiries-about-studies-not-listed-on-the-vivli-platform/ link to request access to study data. The research proposal should provide the scientific rationale of the planned analysis and explain the potential public interest, and will be reviewed by Boehringer Ingelheim and Pharmaxis, as well as an external independent review panel of experts. In addition to anonymised clinical study data, upon request, Boehringer Ingelheim provides the redacted study protocol, statistical analysis plan, annotated case report forms, clinical study reports and data specifications, as appropriate. The Study Protocol and Statistical Analysis Plan are available as Supplementary Notes 1 and 2 in the Supplementary Information file. The remaining data are available within the Article, Supplementary Information. Controlled access to patient level clinical study data is an essential element of data protection requirements. Further information can be found at https://www.mystudywindow.com/msw/datasharing. Source data are provided with this paper.
