# Supplementary material for: A randomised Phase IIa trial of amine oxidase copper-containing 3 (AOC3) inhibitor BI 1467335 in adults with non-alcoholic steatohepatitis
Source: Nat Commun. 2023 Nov 6;14:7151. doi: 10.1038/s41467-023-42398-w (PMC10628239; doi:10.1038/s41467-023-42398-w)
Supplement: Supplementary file 1 — Supplementary Information [file 41467_2023_42398_MOESM1_ESM.pdf]

Supplementary information

Title: A randomised phase IIa trial of amine oxidase copper-containing 3 (AOC3) inhibitor BI 1467335 in adults with non-alcoholic steatohepatitis

Authors: Philip N Newsome, Arun J Sanyal, Guy Neff, Jörn M Schattenberg, Vlad Ratziu, Judith Ertle, Jasmin Link, Alison Mackie, Corinna Schoelch, Eric Lawitz, BI 1467335 NASH Phase IIa trial team

Table of Contents

Supplementary methods S3
Removal of individual patients in cases of increased liver enzymes S3
Supplementary Figure. 1. Trial procedures in cases of further increased liver enzymes after randomisation. S5
Global protocol amendments S6
Supplementary tables S7
Supplementary Table 1. AOC3 activity relative to baseline over time (Week 2 to Week 12; PPS) S7
Supplementary Table 2. AOC3 concentration over time (baseline to follow-up; FAS). S7
Supplementary Table 3. AST relative to baseline at Week 12 (PPS). S8
Supplementary Table 4. GGT relative to baseline at Week 12 (PPS). S9
Supplementary Table 5. CK-18 total relative to baseline at Week 12 (PPS). S10
Supplementary Table 6. AP relative to baseline at Week 12 (PPS). S11
Supplementary Table 7. ALT concentration over time (baseline to follow-up; FAS). S12
Supplementary Table 8. AST concentration over time (baseline to follow-up; FAS). S13
Supplementary Table 9. AP concentration over time (baseline to follow-up; FAS). S14
Supplementary Table 10. GGT concentration over time (baseline to follow-up; FAS). S15
Supplementary Table 11. CK-18 caspase concentration over time (baseline to follow-up; FAS). S16
Supplementary Table 12. CK-18 total concentration over time (baseline to follow-up; FAS). S17
Supplementary Table 13. FPG concentration over time (baseline to follow-up; FAS). S18
Supplementary Table 14. Fasting insulin concentration over time (baseline to follow-up; FAS). S19
Supplementary Table 15. Fasting cholesterol concentration over time (baseline to follow-up; FAS). S20
Supplementary Table 16. Fasting triglyceride concentration over time (baseline to follow-up; FAS). S21
Supplementary Table 17. Interferon γ concentration over time (baseline to follow-up; FAS). S21
Supplementary Table 18. Interleukin 1β concentration over time (baseline to follow-up; FAS). S22

Supplementary Table 19. Interleukin 6 concentration over time (baseline to follow-up; FAS). S22
Supplementary Table 20. Interleukin 8 concentration over time (baseline to follow-up; FAS). S23
Supplementary Table 21. APRI score over time (baseline to follow-up; FAS). S23
Supplementary Table 22. Fib-4 score over time (baseline to follow-up, FAS). S24
Supplementary Table 23. ELF test score over time (baseline to follow-up; FAS). S24
Supplementary Table 24. NAFLD fibrosis score over time (baseline to follow-up; FAS). S25
Supplementary Table 25. Pro-C3 concentration over time (baseline to follow-up; FAS). S25
Supplementary Table 26. Ethical Approval. S26
Supplementary figures S29
Supplementary Figure 2. Non-significant linear dose–response relationships of AST, AP, GGT and CK-18 total relative to baseline at Week 12 (PPS). S29
Supplementary Figure 3. Placebo-corrected ALT, AST, AP, GGT, CK-18 caspase and CK-18 total relative to baseline MMRM results over time (PPS). S30
Supplementary Figure 4. Change of Pro-C3 from baseline versus ALT change from baseline over time (FAS). S31
Supplementary Figure 5. Change of Pro-C3 from baseline versus AST change from baseline over time (FAS). S32
Supplementary Figure 6. Mean change (SD) of Pro-C3 from baseline over time (FAS). S33
Trial Collaborators S34
Full list of BI 1467335 NASH Phase IIa trial team collaborators S34
Full list of collaborator affiliations S34
Supplementary Notes S36
Supplementary Note 1. The CONSORT checklist. S36
Supplementary Note 2. Study Protocol. S39
Supplementary Note 3. Statistical analysis plan. S10139

Supplementary methods

Removal of individual patients in cases of increased liver enzymes

Treatment emergent adverse events of special interest (TEAESIs) were liver injury events (alanine aminotransferase [ALT] and/or aspartate aminotransferase [AST] 5–8 × baseline or >300 U/L in patients with ALT and/or AST > upper limit of normal [ULN] at baseline; AST 3–8 × ULN in patients with normal AST at baseline). Trial-specific procedures for the removal of individual patients were defined in cases of increased liver enzymes (AST, ALT and total bilirubin) after randomisation and are summarised in Supplementary Figure 1.

In suspected cases of elevated liver enzymes, treatment was to be temporarily interrupted, the increased laboratory values were to be confirmed by a second measurement (i.e. the patient was notified to stop the trial medication and requested to attend an additional unscheduled visit within 48 hours of initial laboratory hepatic injury alert/notification or as soon as possible if timelines could not be met) and other potential causes for liver injury had to be excluded.

1. If the values were confirmed or even further increased, the trial medication was permanently stopped, and the patient was withdrawn from treatment and was monitored closely on a weekly basis until resolution or stabilisation in the following cases:

- AST normal at baseline and:
    - AST >8 × ULN after randomisation
    - AST 5–8 × ULN for more than 2 weeks after randomisation
- ALT and/or AST abnormal at baseline and:
    - ALT and/or AST >8 × baseline OR >500 U/L as an absolute value, whichever was lower

After resolution or stabilisation, the patient was encouraged to continue the trial and complete all visits. If the patient was not willing to continue, the patient was withdrawn, and then encouraged to undergo procedures for early discontinuation and follow-up.

2. If the values were confirmed, the trial medication stayed interrupted and the patient was monitored closely until resolution or stabilisation in the following cases:

- AST normal at baseline and:
    - AST 3–8 × ULN AND total bilirubin >2 mg/dL after randomisation
    - AST 3–8x ULN OR total bilirubin >2 mg/dL AND the presence of clinical symptoms of hepatic injury (e.g. encephalopathy, nausea, vomiting, pruritus, severe fatigue) after randomisation
- ALT and/or AST abnormal at baseline and:

  - ALT and/or AST 5–8 × baseline OR >300 U/L as an absolute value (whichever was lower) AND total bilirubin >2 mg/dL after randomisation
  - ALT and/or AST 5–8 × baseline OR >300 U/L as an absolute value (whichever was lower) OR total bilirubin >2 mg/dL AND the presence of clinical symptoms of hepatic injury (as above) after randomisation

If the values returned to baseline, a re-challenge with the trial medication could be considered after discussion with the sponsor. After re-starting the trial medication, the patient was to be monitored on a weekly basis until end of trial. If the initially elevated liver enzyme(s) that led to interruption increased again by more than 2 × baseline levels, the trial medication was to be discontinued permanently. The patient continued the trial, unless they disagreed in which case the patient was encouraged to undergo procedures for early discontinuation and follow-up.

Supplementary Figure. 1. Trial procedures in cases of further increased liver enzymes after randomisation.

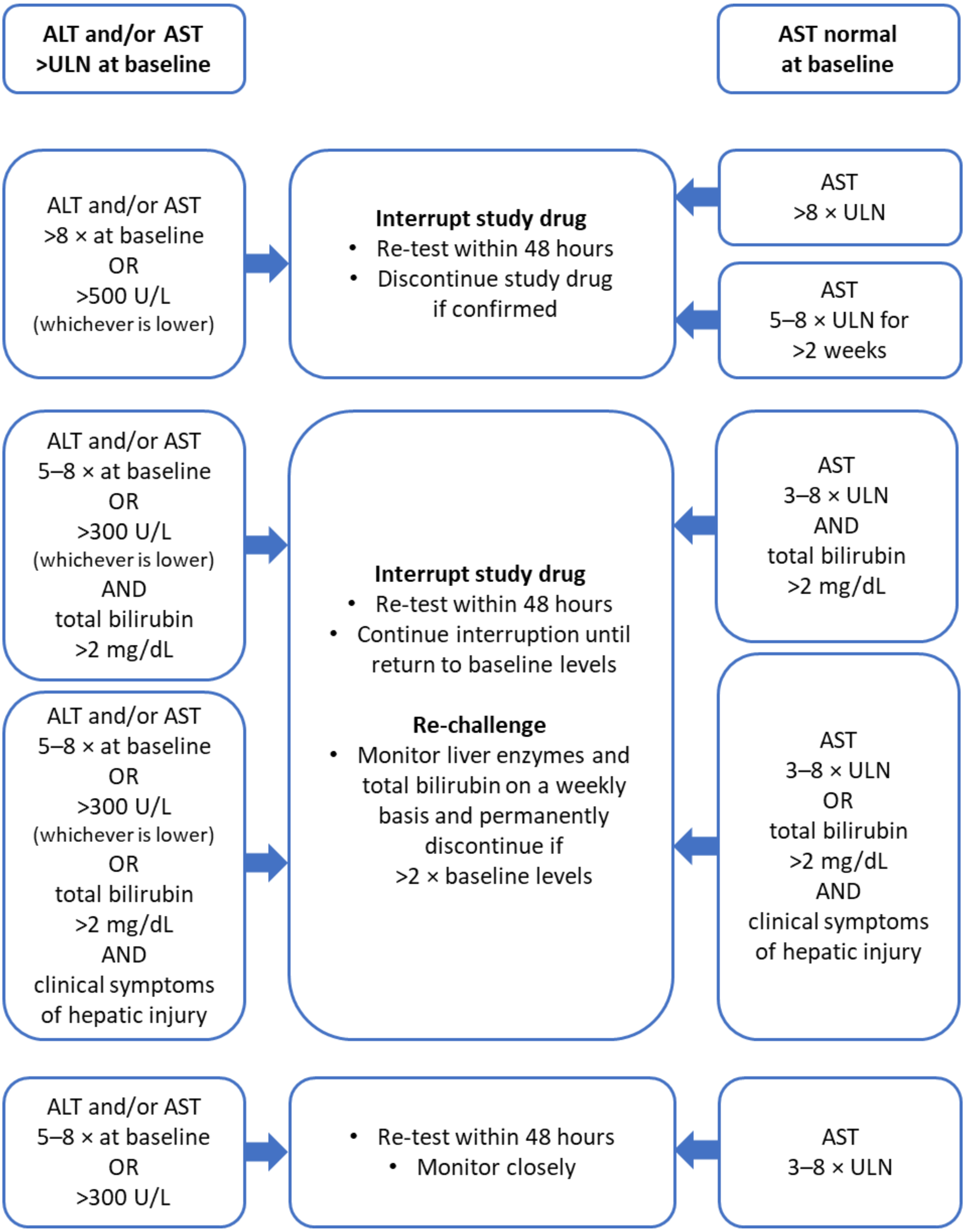

ALT, alanine aminotransferase; AST, aspartate aminotransferase; ULN, upper limit of normal.

Global protocol amendments

Amendment 1, implemented on 23 March 2017, introduced clarifications to the schedule of assessments, the timing of the procedures regarding the PK and PD analyses, the inclusion and exclusion criteria, the criteria for the removal of individual patients, the restrictions regarding concomitant treatments, the definition of the residual effect period and the procedures for elevated transaminases. Since the residual effect period was to be explored in this trial, it was considered by default as the entire follow-up period (i.e. time from the last administration of trial medication until the individual patient's end of trial). All adverse events which occurred through the treatment phase until the entire follow-up period were considered as on-treatment adverse events.

Amendment 2, implemented on 19 May 2017, added a modified algorithm for symptomatic patients with elevated bilirubin levels and describing the possible implementation of an interim PK/PD analysis. The scope of the interim analysis was extended beyond PK/PD and included biomarker and safety analyses.

Amendment 3, implemented on 16 Mar 2018, introduced clinical imaging as an optional screening procedure, the option of 2 consecutive ALT measurements during screening for patients with local ALT value older than 3 months, the biomarker urine collection, and blood sampling for platelet-rich plasma. To align with the investigator's brochure, the dug profile and the wording of the criteria required for randomisation of women of childbearing potential were updated. Additional restrictions regarding concomitant treatment and food intake were implemented leading to the deletion of one exclusion criterion. Urine creatinine was added for urine biomarker assessment. Urine and blood samples were added to evaluate the potential MAO-B inhibitory effect of BI 1467335 in patients.

Amendment 4, implemented on 19 Jul 2018, clarified trial procedures in the footnotes of the schedule of assessments and updated the list of restrictions regarding concomitant medications (bupropion, triptans, linezolide, tedizolid, methylene blue, lithium and pethidine added to the list), and diet and lifestyle (avoidance of large amounts of tyramine-rich food while taking BI 1467335).

Amendment 5, introduced on 12 September 2018, reduced the sample size from 147 to 108 randomised patients due to a lower expected variability for ALT based on new external and blinded internal data. Further clarifications to restricted concomitant medications were also implemented allowing for the short-term treatment of acute conditions such as headache or sinusitis, if not otherwise prohibited.

Supplementary tables

Supplementary Table 1. AOC3 activity relative to baseline over time (Week 2 to Week 12; PPS)

AOC3 activity, adjusted mean ± SE, %	Placebo (n=32)	BI 1467335			
		1 mg (n=16)	3 mg (n=16)	6 mg (n=16)	10 mg (n=32)
Week 2	95.3 ± 2.2[a]	31.4 ± 3.3[b]	14.4 ± 3.3[b]	10.7 ± 3.2[b]	5.3 ± 2.3[c]
Week 4	100.6 ± 3.0[d]	29.0 ± 4.6[b]	11.3 ± 4.8[b]	6.6 ± 4.5[e]	2.5 ± 3.2[a]
Week 6	100.9 ± 2.2[f]	24.3 ± 3.3[g]	12.0 ± 3.3[g]	9.7 ± 3.4[h]	3.8 ± 2.4[i]
Week 8	100.1 ± 2.3[d]	29.1 ± 3.5[b]	14.4 ± 3.5[b]	6.3 ± 3.5[g]	4.0 ± 2.5[c]
Week 12	90.4 ± 3.2[d]	26.5 ± 5.0[g]	10.4 ± 5.0[b]	5.0 ± 5.0[b]	3.3 ± 3.5[c]

[a]n=27; [b]n=13; [c]n=25; [d]n=30; [e]n=14; [f]n=29; [g]n=12; [h]n=11; [i]n=24.

AOC3, amine oxidase copper-containing 3; CV, coefficient of variation; PPS, per-protocol set; SE, standard error.

Supplementary Table 2. AOC3 concentration over time (baseline to follow-up; FAS).

AOC3 concentration, mean ± SD, µg/L	Placebo (n=32)	BI 1467335			
		1 mg (n=16)	3 mg (n=16)	6 mg (n=16)	10 mg (n=32)
Baseline	487 ± 159[a]	561 ± 192[b]	498 ± 141	535 ± 144	526 ± 137
Week 2	473 ± 119[c]	561 ± 147[d]	468 ± 126	507 ± 119[e]	540 ± 151[f]
Week 4	519 ± 149[f]	485 ± 136[b]	489 ± 137[g]	513 ± 114[g]	543 ± 143[h]
Week 6	522 ± 177[c]	568 ± 181[d]	492 ± 138[g]	555 ± 118[b]	526 ± 156[i]
Week 8	527 ± 162[c]	542 ± 204[b]	488 ± 180[g]	604 ± 223[g]	538 ± 162[i]
Week 12	495 ± 172	503 ± 188[d]	503 ± 171[g]	468 ± 119[b]	499 ± 143[j]
Follow-up (Week 16)	536 ± 200[i]	535 ± 205[d]	506 ± 158 [k]	515 ± 153[g]	508 ± 136[j]

[a]n=30; [b]n=13; [c]n=31; [d]n=12; [e]n=15; [f]n=28; [g]n=14; [h]n=29; [i]n=27; [j]n=25, [k]n=11.

AOC3, amine oxidase copper-containing 3; FAS, full analysis set.

Supplementary Table 3. AST relative to baseline at Week 12 (PPS).

		Relative to baseline, %			Comparison versus placebo, %		
Treatment group	n	Adjusted mean	SE	90% CI	Adjusted mean	SE	90% CI
Placebo	27	93.8	105.9	85.2, 103.2	–	–	–
BI 1467335 1 mg	12	105.2	108.9	91.2, 121.3	11.4	10.5	–5.8, 28.6
BI 1467335 3 mg	13	90.1	108.7	78.4, 103.6	–3.7	9.2	–18.8, 11.5
BI 1467335 6 mg	14	84.1	108.3	73.6, 99.1	–9.7	8.6	–23.8, 4.5
BI 1467335 10 mg	23	87.8	106.3	79.4, 97.2	–5.9	7.6	–18.5, 6.6

AST, aspartate aminotransferase; PPS, per-protocol set.

Supplementary Table 4. GGT relative to baseline at Week 12 (PPS).

		Relative to baseline, %			Comparison versus placebo, %		
Treatment group	n	Adjusted mean	SE	90% CI	Adjusted mean	SE	90% CI
Placebo	29	91.4	105.2	83.9, 99.5	–	–	–
BI 1467335 1 mg	12	99.4	108.3	87.2, 113.4	8.1	9.2	–7.1, 23.2
BI 1467335 3 mg	13	92.4	108.1	81.2, 105.2	1.1	8.5	–13.0, 15.1
BI 1467335 6 mg	14	99.5	107.7	88.0, 112.6	8.1	8.8	–6.5, 22.5
BI 1467335 10 mg	28	83.7	105.4	76.7, 91.3	–7.7	6.4	–18.2, –2.9

GGT, gamma-glutamyl transferase; PPS, per-protocol set.

Supplementary Table 5. CK-18 total relative to baseline at Week 12 (PPS).

		Relative to baseline, %			Comparison versus placebo, %		
Treatment group	n	Adjusted mean	SE	90% CI	Adjusted mean	SE	90% CI
Placebo	28	92.4	109.1	80.0, 106.9	–	–	–
BI 1467335 1 mg	11	128.3	114.6	102.3, 161.0	35.9	19.2	4.3, 67.5
BI 1467335 3 mg	13	99.6	113.8	80.3, 123.4	7.1	15.0	–17.6, 31.9
BI 1467335 6 mg	14	94.7	113.1	77.2, 116.3	2.3	14.2	–21.1, 25.7
BI 1467335 10 mg	26	81.5	109.4	70.2, 94.5	–11.0	10.9	–28.9, 6.9

CK-18 total, total cytokeratin 18; PPS, per-protocol set.

Supplementary Table 6. AP relative to baseline at Week 12 (PPS).

		Relative to baseline, %			Comparison versus placebo, %		
Treatment group	n	Adjusted mean	SE	90% CI	Adjusted mean	SE	90% CI
Placebo	29	96.6	102.3	93.0, 100.4	–	–	–
BI 1467335 1 mg	12	97.6	103.6	92.1, 103.4	1.0	4.1	–5.8, 7.7
BI 1467335 3 mg	13	100.5	103.5	95.0, 106.4	3.9	4.0	–2.8, 10.5
BI 1467335 6 mg	14	98.5	103.3	93.2, 104.0	1.9	3.9	–4.5, 8.2
BI 1467335 10 mg	28	94.7	102.3	91.1, 98.4	–1.9	3.1	–7.0, 3.2

AP, alkaline phosphatase; PPS, per-protocol set.

Supplementary Table 7. ALT concentration over time (baseline to follow-up; FAS).

ALT, mean ± SD, U/L	Placebo (n=32)	BI 1467335			
		1 mg (n=16)	3 mg (n=16)	6 mg (n=16)	10 mg (n=32)
Baseline	78.0 ± 28.8	89.6 ± 24.7	82.3 ± 25.5	82.8 ± 29.3	88.7 ± 51.4
Week 2	72.9 ± 29.9[a]	83.3 ± 19.1	77.1 ± 25.5	81.3 ± 27.4[b]	81.7 ± 55.1[c]
Week 4	79.8 ± 47.9[a]	92.1 ± 28.0[d]	76.7 ± 22.8[d]	78.8 ±22.6[e]	74.5 ± 31.7[c]
Week 6	76.3 ±40.7[a]	85.7 ± 20.7[b]	74.7 ± 25.9[d]	75.5 ± 30.0[f]	71.7 ± 30.0[c]
Week 8	74.8 ±36.3	94.7 ± 26.0[d]	81.0 ± 30.6[d]	73.9 ± 27.4[d]	73.0 ± 31.7[g]
Week 12	75.4 ±36.2[a]	89.4 ±24.8[f]	73.9 ± 31.5[d]	74.9 ± 36.2[d]	65.6 ± 30.4[g]
Follow-up (Week 16)	78.8 ± 43.3	88.8 ± 26.6[f]	72.2 ± 31.1[f]	78.9 ± 43.3	80.8 ± 56.7[c]

[a]n=31; [b]n=15; [c]n=29; [d]n=14; [e]n=12; [f]n=13 [g]n=27.

ALT, alanine aminotransferase; FAS, full analysis set.

Supplementary Table 8. AST concentration over time (baseline to follow-up; FAS).

AST, mean ± SD, U/L	Placebo (n=32)	BI 1467335			
		1 mg (n=16)	3 mg (n=16)	6 mg (n=16)	10 mg (n=32)
Baseline	50.1 ± 18.5	61.8 ± 31.5	66.1 ± 38.7	53.0 ± 18.5	60.2 ± 38.5
Week 2	47.2 ± 16.8[a]	54.6 ± 13.0	59.0 ± 28.4[b]	53.7 ± 17.3[b]	64.0 ± 75.2[c]
Week 4	46.9 ± 24.3[a]	63.7 ± 22.2[d]	69.6 ± 34.6[e]	52.3 ± 19.7[f]	58.6 ± 45.5[g]
Week 6	49.6 ± 24.4[h]	66.8 ± 49.0[b]	61.6 ± 31.0[d]	54.5 ± 26.1[e]	53.0 ± 37.1[g]
Week 8	49.8 ± 23.6	67.6 ± 24.8[d]	61.4 ± 28.3[d]	53.4 ± 22.5[d]	54.4 ± 40.0[c]
Week 12	48.4 ± 22.0[i]	58.0 ± 16.2[e]	57.4 ± 35.7[d]	50.6 ± 26.3[d]	49.2 ± 23.4[j]
Follow-up (Week 16)	58.6 ± 52.2[h]	57.9 ± 20.6[e]	54.5 ± 24.9[e]	56.4 ± 36.3	65.3 ± 65.2[a]

[a]n=29; [b]n=15; [c]n=27; [d]n=14; [e]n=13; [f]n=12; [g]n=28; [h]n=31; [i]n=30; [j]n=23.

AST, aspartate aminotransferase; FAS, full analysis set.

Supplementary Table 9. AP concentration over time (baseline to follow-up; FAS).

AP, mean ± SD, U/L	Placebo (n=32)	BI 1467335			
		1 mg (n=16)	3 mg (n=16)	6 mg (n=16)	10 mg (n=32)
Baseline	76.2 ± 21.8	86.1 ± 25.1	90.1 ± 25.3	99.7 ± 44.7	88.4 ± 33.9
Week 2	74.3 ± 23.2[a]	84.8 ± 31.0	86.7 ± 25.0	100.4 ± 45.3[b]	87.8 ± 31.8[a]
Week 4	75.6 ±26.2[a]	85.4 ± 26.5[c]	89.4 ± 24.2[b]	94.8 ± 35.6[d]	85.9 ± 31.7[e]
Week 6	75.5 ± 22.7[a]	83.9 ± 27.6[b]	87.4 ± 23.6[c]	94.8 ± 38.8[d]	85.9 ± 33.8[f]
Week 8	73.5 ± 22.0	80.2 ± 23.0[c]	90.4 ± 26.2[c]	92.4 ± 36.8[c]	87.5 ± 33.9[g]
Week 12	74.7 ± 24.4	79.8 ± 20.2[d]	86.4 ± 22.6[c]	95.0 ± 43.1[c]	81.8 ± 25.8[h]
Follow-up (Week 16)	78.7 ± 25.4	85.4 ± 22.3[c]	86.7 ± 22.2[d]	100.9 ± 51.1	86.1 ± 30.3[f]

[a]n=31; [b]n=15; [c]n=14; [d]n=13; [e]n=30; [f]n=29; [g]n=27; [h]n=28.

AP, alkaline phosphatase; FAS, full analysis set.

Supplementary Table 10. GGT concentration over time (baseline to follow-up; FAS).

GGT, mean ± SD, U/L	Placebo (n=32)	BI 1467335			
		1 mg (n=16)	3 mg (n=16)	6 mg (n=16)	10 mg (n=32)
Baseline	82.2 ± 51.3	138.3 ±319.1	170.4 ± 140.0	111.3 ± 136.7	128.4 ± 136.3
Week 2	76.1 ± 44.6[a]	107.8 ± 213.6	162.3 ± 140.5	108.7 ± 154.6[b]	114.0 ± 119.4[a]
Week 4	78.6 ± 46.5[a]	157.1 ± 355.2[c]	169.0 ± 153.8[b]	81.9 ± 69.5[d]	118.8 ± 131.4[e]
Week 6	77.9 ± 49.1[a]	186.4 ± 494.2[b]	172.6 ± 146.1[c]	82.8 ± 74.4[d]	116.6 ± 119.4[f]
Week 8	76.3 ± 45.0	129.5 ± 257.8[c]	158.0 ± 139.7[c]	78.5 ± 71.8[c]	129.9 ± 149.3[g]
Week 12	82.0 ± 69.9	59.3 ± 38.5[d]	139.8 ± 129.7[c]	92.7 ± 88.4[c]	103.0 ± 100.1[h]
Follow-up (Week 16)	90.1 ± 75.0	68.8 ± 50.6[c]	125.3 ± 117.9[d]	127.1 ± 172.7	98.1 ± 97.4[f]

[a]n=31; [b]n=15; [c]n=14; [d]n=13; [e]n=30; [f]n=29; [g]n=27; [h]n=28.

FAS, full analysis set; GGT, gamma-glutamyl transferase.

Supplementary Table 11. CK-18 caspase concentration over time (baseline to follow-up; FAS).

CK-18 caspase, mean ± SD, U/L	Placebo (n=32)	BI 1467335			
		1 mg (n=16)	3 mg (n=16)	6 mg (n=16)	10 mg (n=32)
Baseline	574.0 ± 556.1	726.1 ± 386.0	866.5 ± 916.4	589.5 ± 493.0	677.7 ± 670.1
Week 2	515.2 ± 447.8[a]	867.5 ± 466.2	767.9 ± 728.4	493.1 ± 333.2	663.4 ± 745.7[a]
Week 4	622.0 ± 729.3	942.5 ± 494.7[b]	843.9 ± 966.3[c]	473.3 ± 298.7[d]	632.7 ± 514.9[e]
Week 6	685.5 ± 758.9[f]	820.1 ± 600.9[c]	919.0 ± 1032.9[d]	552.4 ± 419.7[b]	542.6 ± 416.7[e]
Week 8	621.6 ± 746.9	1139.1 ± 730.8[d]	679.4 ± 450.5[b]	686.9 ± 537.5[d]	621.7 ± 736.4[g]
Week 12	617.7 ± 678.8	989.5 ± 612.6[b]	721.9 ± 766.4[b]	479.6 ± 318.5[d]	493.6 ± 339.1[h]
Follow-up (Week 16)	551.3 ± 547.8[f]	908.1 ± 542.9[d]	694.8 ± 626.9[b]	502.4 ± 403.4	778.4 ± 975.5[i]

[a]n=30; [b]n=13; [c]n=15; [d]n=14; [e]n=29; [f]n=31; [g]n=27; [h]n=26; [i]n=28.

CK-18 caspase, caspase-cleaved cytokeratin 18; FAS, full analysis set.

Supplementary Table 12. CK-18 total concentration over time (baseline to follow-up; FAS).

CK-18 total, mean ± SD, U/L	Placebo (n=32)	BI 1467335			
		1 mg (n=16)	3 mg (n=16)	6 mg (n=16)	10 mg (n=32)
Baseline	1142.3 ± 725.3	1400.8 ± 592.0[a]	1594.6 ± 1060.2	1015.9 ± 445.9	1242.6 ± 785.2
Week 2	1005.8 ± 543.9[b]	1484.9 ± 313.4	1364.4 ± 700.7[a]	936.1 ± 322.9[a]	1208.2 ± 906.2[b]
Week 4	1208.2 ± 1196.7	2083.2 ± 1319.3[c]	1429.5 ± 1148.3[d]	1020.4 ± 439.9[d]	1204.6 ± 721.4[e]
Week 6	1166.5 ± 857.6[f]	1460.0 ± 587.6[d]	1600.4 ± 1849.3[c]	1088.3 ± 554.6[c]	1063.4 ± 610.3[e]
Week 8	1126.4 ± 829.0	1778.4 ± 929.2[c]	1355.9 ± 750.3[d]	1257.3 ± 616.3[d]	1216.3 ± 1006.1[g]
Week 12	1091.5 ± 840.3[f]	1628.5 ± 733.2[c]	1384.9 ± 750.3[d]	1074.8 ± 533.7[d]	1057.8 ± 580.6[h]
Follow-up (Week 16)	998.0 ± 579.9[f]	1483.0 ± 695.1[d]	1297.0 ± 842.8[i]	948.1 ± 464.7	1284.2 ± 1138.6[j]

[a]n=15; [b]n=30; [c]n=12; [d]n=14; [e]n=29; [f]n=31; [g]n=27; [h]n=26; [i]n=13; [j]n=28.

CK-18 total, total cytokeratin 18; FAS, full analysis set.

Supplementary Table 13. FPG concentration over time (baseline to follow-up; FAS).

FPG, mean ± SD, mg/dL	Placebo (n=32)	BI 1467335			
		1 mg (n=16)	3 mg (n=16)	6 mg (n=16)	10 mg (n=32)
Baseline	113.4 ± 31.3[a]	122.1 ± 38.3	125.0 ± 48.6	123.9 ± 35.3	125.9 ± 36.6
Week 2	112.5 ± 29.8[a]	124.1 ± 32.9[b]	125.4 ± 42.9	126.1 ± 35.5	124.5 ± 43.0[a]
Week 4	119.3 ± 28.7	119.8 ± 24.1[c]	130.0 ± 40.3[b]	127.9 ± 41.0[c]	129.6 ± 44.2[d]
Week 6	114.5 ± 28.3[a]	124.8 ± 38.8[b]	137.1 ± 50.5[c]	130.2 ± 41.5[e]	125.9 ± 46.7[f]
Week 8	115.3 ± 32.5	118.8 ± 32.0[c]	131.6 ± 48.2[c]	126.7 ± 40.5[c]	129.2 ± 50.7[f]
Week 12	113.1 ± 24.8	124.3 ± 30.0[e]	134.9 ± 46.8[c]	131.9 ± 43.7[c]	130.9 ± 50.2[f]
Follow-up (Week 16)	113.9 ± 20.2	117.7 ± 41.9[c]	130.2 ± 40.5[e]	125.7 ± 36.6	134.2 ± 51.9[g]

[a]n=31; [b]n=15; [c]n=14; [d]n=30; [e]n=13; [f]n=28, [g]n=26.

FAS, full analysis set; FPG, fasting plasma glucose.

Supplementary Table 14. Fasting insulin concentration over time (baseline to follow-up; FAS).

Fasting insulin, mean ± SD, pmol/L	Placebo (n=32)	BI 1467335			
		1 mg (n=16)	3 mg (n=16)	6 mg (n=16)	10 mg (n=32)
Baseline	116.6 ± 64.6[a]	168.2 ± 96.8	81.4 ± 42.0	145.2 ± 159.1	116.4 ± 76.9
Week 2	114.9 ± 56.7[a]	172.6 ± 147.1[b]	85.9 ± 52.4	148.5 ± 224.6[b]	111.7 ± 53.0[a]
Week 4	131.8 ± 82.5[c]	152.3 ± 119.3[d]	96.2 ± 54.0[b]	126.7 ± 120.5[d]	117.6 ± 71.6[e]
Week 6	119.2 ± 51.8[c]	181.9 ± 88.2[d]	91.5 ± 48.5[d]	142.4 ± 145.4[f]	104.0 ± 56.6[a]
Week 8	120.9 ± 64.5	152.2 ± 88.4[d]	84.8 ± 34.5[d]	108.8 ± 72.7[d]	104.3 ± 58.4[g]
Week 12	112.1 ± 66.8[c]	178.4 ± 107.4[h]	94.3 ± 41.7[d]	159.7 ± 229.2[d]	102.9 ± 60.1[i]
Follow-up (Week 16)	103.6 ± 47.9[c]	184.0 ± 126.1[d]	89.9 ± 50.4[h]	132.4 ± 149.1[b]	117.2 ± 81.6[j]

[a]n=29; [b]n=15; [c]n=31; [d]n=14; [e]n=27; [f]n=12; [g]n=28; [h]n=13; [i]n=25; [j]n=26.

FAS, full analysis set.

Supplementary Table 15. Fasting cholesterol concentration over time (baseline to follow-up; FAS).

Fasting cholesterol, mean ± SD, mg/dL	Placebo (n=32)	BI 1467335			
		1 mg (n=16)	3 mg (n=16)	6 mg (n=16)	10 mg (n=32)
Baseline	185.4 ± 40.7[a]	196.0 ± 45.7	193.5 ± 38.4	171.1 ± 44.3	206.4 ± 45.7
Week 2	179.9 ± 40.0[a]	181.2 ± 35.1[b]	191.3 ± 42.0	174.3 ± 54.4	195.5 ± 48.7[a]
Week 4	185.5 ± 42.0	190.6 ± 42.2[c]	186.2 ± 36.5[b]	167.4 ± 43.3[d]	201.4 ± 44.3[e]
Week 6	180.6 ± 45.1[a]	195.8 ± 63.6[b]	190.3 ± 41.1[d]	175.7 ± 39.1[c]	200.1 ± 43.7[f]
Week 8	180.4 ± 43.7	195.8 ± 43.0[d]	185.1 ± 39.1[d]	177.1 ± 46.4[d]	200.9 ± 38.9[g]
Week 12	176.0 ± 38.5	185.9 ± 33.8[c]	189.8 ± 43.2[d]	174.4 ± 49.2[d]	192.1 ± 37.4[h]
Follow-up (Week 16)	186.3 ± 42.2	177.7 ± 30.6[d]	180.5 ± 44.3[c]	175.1 ± 48.7	200.3 ± 45.2[g]

[a]n=31; [b]n=15; [c]n=13; [d]n=14; [e]n=30; [f]n=29; [g]n=27; [h]n=28.

FAS, full analysis set.

Supplementary Table 16. Fasting triglyceride concentration over time (baseline to follow-up; FAS).

Fasting triglyceride, mean ± SD, mg/dL	Placebo (n=32)	BI 1467335			
		1 mg (n=16)	3 mg (n=16)	6 mg (n=16)	10 mg (n=32)
Baseline	179.5 ± 120.7[a]	168.4 ± 64.4	164.3 ± 79.9	175.4 ± 135.7	185.3 ± 98.5
Week 2	163.5 ± 71.3[a]	187.2 ± 117.6[b]	173.5 ± 122.8	146.2 ± 102.2	170.6 ± 83.2[a]
Week 4	170.0 ± 82.7	201.3 ± 179.6[c]	165.0 ± 78.2[b]	158.1 ± 108.0[c]	172.7 ± 82.4[d]
Week 6	164.3 ± 68.5[a]	254.9 ± 358.2[b]	166.4 ± 100.3[e]	183.1 ± 176.7[c]	174.2 ± 84.9[f]
Week 8	171.3 ± 96.6	146.3 ± 39.5[e]	170.2 ± 107.8[e]	178.7 ± 150.0[e]	181.6 ± 75.0[g]
Week 12	165.1 ± 67.0	165.2 ± 77.9[c]	167.9 ± 91.3[e]	172.7 ± 121.7[e]	182.0 ± 115.6[h]
Follow-up (Week 16)	167.1 ± 88.4	153.7 ± 51.8[e]	177.2 ± 147.6[c]	149.9 ± 94.0	189.1 ± 115.9[g]

[a]n=31; [b]n=15; [c]n=13; [d]n=30; [e]n=14; [f]n=29; [g]n=27; [h]n=28.

FAS, full analysis set.

Supplementary Table 17. Interferon γ concentration over time (baseline to follow-up; FAS).

Interferon γ, mean ± SD, ng/L	Placebo (n=32)	BI 1467335			
		1 mg (n=16)	3 mg (n=16)	6 mg (n=16)	10 mg (n=32)
Baseline	14.9 ± 0.0	14.9 ± 0.0[a]	14.9 ± 0.0	14.9 ± 0.0	15.0 ± 0.8[b]
Week 4	14.9 ± 0.0[b]	15.6 ± 2.5[c]	15.0 ± 0.4[a]	14.9 ± 0.0[a]	14.9 ± 0.1[b]
Week 12	14.9 ± 0.0[b]	14.9 ± 0.0[d]	20.3 ± 20.4[c]	15.0 ± 0.5[c]	15.6 ± 2.5[e]
Follow-up (Week 16)	14.9 ± 0.0	14.9 ± 0.0[c]	15.4 ± 1.7[d]	14.9 ± 0.0	14.9 ± 0.0[f]

[a]n=15; [b]n=31; [c]n=14; [d]n=13; [e]n=25; [f]n=28.

FAS, full analysis set.

Supplementary Table 18. Interleukin 1β concentration over time (baseline to follow-up; FAS).

[a]n=15; [b]n=31; [c]n=14; [d]n=13; [e]n=25; [f]n=28.

Interleukin 1β, mean ± SD, ng/L	Placebo (n=32)	BI 1467335			
		1 mg (n=16)	3 mg (n=16)	6 mg (n=16)	10 mg (n=32)
Baseline	4.3 ± 0.0	4.3 ± 0[a]	4.3 ± 0.0	4.3 ± 0.0	4.3 ± 0.0[b]
Week 4	4.3 ± 0.0[b]	4.3 ± 0.0[c]	4.3 ± 0.0[a]	4.3 ± 0.0[a]	4.3 ± 0.0[b]
Week 12	4.3 ± 0.0[b]	4.3 ± 0.0[d]	4.3 ± 0.0[c]	4.3 ± 0.0[c]	4.3 ± 0.0[e]
Follow-up (Week 16)	4.3 ± 0.0	4.3 ± 0.0[c]	4.3 ± 0.0[d]	4.3 ± 0.0	4.3 ± 0.0[f]

FAS, full analysis set.

Supplementary Table 19. Interleukin 6 concentration over time (baseline to follow-up; FAS).

Interleukin 6, mean ± SD, μg/L	Placebo (n=32)	BI 1467335			
		1 mg (n=16)	3 mg (n=16)	6 mg (n=16)	10 mg (n=32)
Baseline	3.2 ± 0.0	3.3 ± 0.4[a]	3.3 ± 0.7	3.3 ± 0.6	3.2 ± 0.2[b]
Week 4	3.2 ± 0.2[b]	3.3 ± 0.5[c]	3.6 ± 1.1[a]	3.2 ± 0.0[a]	3.2 ± 0.2[b]
Week 12	3.3 ± 0.7[b]	3.2 ± 0.3[d]	3.5 ± 0.8[c]	3.2 ± 0.0[c]	3.2 ± 0.0[e]
Follow-up (Week 16)	3.4 ± 1.2	3.2 ± 0.1[c]	3.2 ± 0.0[d]	3.2 ± 0.0	3.2 ± 0.1[f]

[a]n=15; [b]n=31; [c]n=14; [d]n=13; [e]n=25; [f]n=28.

FAS, full analysis set.

Supplementary Table 20. Interleukin 8 concentration over time (baseline to follow-up; FAS).

Interleukin 8, mean ± SD, µg/L	Placebo (n=32)	BI 1467335			
		1 mg (n=16)	3 mg (n=16)	6 mg (n=16)	10 mg (n=32)
Baseline	22.6 ± 13.9	26.4 ± 20.6[a]	35.7 ± 33.8	88.1 ± 199.6	27.0 ± 22.2[b]
Week 4	19.9 ± 8.3[b]	32.5 ± 41.0[c]	30.2 ± 23.7[a]	39.2 ± 56.5[a]	26.8 ± 15.2[b]
Week 12	20.8 ± 9.1[b]	26.0 ± 16.8[d]	30.4 ± 20.0[c]	26.3 ± 18.5[c]	22.6 ± 10.6[e]
Follow-up (Week 16)	21.7 ± 19.6	34.4 ± 40.6[c]	28.9 ± 20.5[d]	27.9 ± 24.5	25.6 ± 14.6[f]

[a]n=15; [b]n=31; [c]n=14; [d]n=13; [e]n=25; [f]n=28.

FAS, full analysis set.

Supplementary Table 21. APRI score over time (baseline to follow-up; FAS).

APRI score, mean ± SD	Placebo (n=32)	BI 1467335			
		1 mg (n=16)	3 mg (n=16)	6 mg (n=16)	10 mg (n=32)
Baseline	0.7 ± 0.3	0.8 ± 0.5	0.9 ± 0.7	0.7 ± 0.2	0.7 ± 0.5
Week 2	0.6 ± 0.3[a]	0.7 ± 0.3	0.8 ± 0.5[b]	0.7 ± 0.3[b]	0.8 ± 0.9[c]
Week 4	0.6 ± 0.4[a]	0.8 ± 0.4[d]	1.0 ± 0.6[e]	0.7 ± 0.3[f]	0.7 ± 0.5[g]
Week 6	0.6 ± 0.4[h]	0.8 ± 0.7[b]	0.7 ± 0.5[e]	0.7 ± 0.4[e]	0.6 ± 0.4[i]
Week 8	0.6 ± 0.3[h]	0.9 ± 0.5[d]	0.8 ± 0.5[d]	0.7 ± 0.3[e]	0.7 ± 0.5[c]
Week 12	0.6 ± 0.4[a]	0.7 ± 0.4[j]	0.8 ± 0.7[e]	0.6 ± 0.4[d]	0.6 ± 0.3[j]
Follow-up (Week 16)	0.8 ± 0.8[h]	0.7 ± 0.4[e]	0.7 ± 0.5[f]	0.7 ± 0.5	0.8 ± 0.9[i]

[a]n=29; [b]n=15; [c]n=26; [d]n=14; [e]n=13; [f]n=12; [g]n=27; [h]n=31; [i]n=28; [j]n=22.

APRI, aspartate aminotransferase to platelet ratio index; FAS, full analysis set.

Supplementary Table 22. Fib-4 score over time (baseline to follow-up, FAS).

FIB-4 score, mean ± SD	Placebo (n=32)	BI 1467335			
		1 mg (n=16)	3 mg (n=16)	6 mg (n=16)	10 mg (n=32)
Baseline	1.4 ± 0.8	1.5 ± 0.9	1.9 ± 1.3	1.2 ± 0.4	1.5 ± 1.0
Week 2	1.4 ± 0.8[a]	1.4 ± 0.8	1.8 ± 1.1[b]	1.2 ± 0.5[b]	1.5 ± 1.3[c]
Week 4	1.2 ± 0.7[a]	1.6 ± 0.8[d]	2.1 ± 1.3[e]	1.2 ± 0.4[f]	1.5 ± 0.9[g]
Week 6	1.3 ± 0.7[h]	1.6 ± 1.1[b]	1.7 ± 1.0[e]	1.3 ± 0.5[e]	1.4 ± 0.8[i]
Week 8	1.4 ± 0.7[h]	1.7 ± 1.0[d]	1.8 ± 1.2[d]	1.3 ± 0.4[e]	1.4 ± 0.9[c]
Week 12	1.4 ± 0.7[a]	1.5 ± 0.9[j]	1.9 ± 1.6[e]	1.2 ± 0.5[d]	1.3 ± 0.8[k]
Follow-up (Week 16)	1.5 ± 0.9[h]	1.4 ± 0.8[e]	1.7 ± 1.4[f]	1.3 ± 0.7	1.7 ± 1.5[i]

[a]n=29; [b]n=15; [c]n=26; [d]n=14; [e]n=13; [f]n=12; [g]n=27; [h]n=31; [i]n=28; [j]n=11; [k]n=22.

FAS, full analysis set; Fib-4 score, Fibrosis-4 score.

Supplementary Table 23. ELF test score over time (baseline to follow-up; FAS).

ELF test score, mean ± SD	Placebo (n=32)	BI 1467335			
		1 mg (n=16)	3 mg (n=16)	6 mg (n=16)	10 mg (n=32)
Baseline	9.4 ± 1.0	9.5 ± 1.1	9.6 ± 1.3	9.1 ± 0.6	9.3 ± 1.2
Week 4	9.5 ± 1.0	9.8 ± 1.2	9.6 ± 1.4[a]	9.0 ± 0.7[a]	9.3 ± 1.3[b]
Week 12	9.5 ± 1.0	9.8 ± 1.2[c]	9.5 ± 1.3[d]	9.1 ± 0.8[d]	9.2 ± 1.2[e]
Follow-up (Week 16)	9.5 ± 1.0	9.6 ± 0.8[d]	9.7 ± 1.1[c]	9.1 ± 0.9	9.3 ± 1.1[f]

[a]n=15; [b]n=31; [c]n=13; [d]n=14; [e]n=27; [f]n=29.

ELF, enhanced liver fibrosis; FAS, full analysis set.

Supplementary Table 24. NAFLD fibrosis score over time (baseline to follow-up; FAS).

NAFLD fibrosis score, mean ± SD	Placebo (n=32)	BI 1467335			
		1 mg (n=16)	3 mg (n=16)	6 mg (n=16)	10 mg (n=32)
Baseline	-1.6 ± 1.5	-1.6 ± 1.5	-1.4 ± 1.3	-1.9 ± 1.0	-1.8 ± 1.6
Week 2	-1.5 ± 1.4[a]	-1.5 ± 1.5	-1.5 ± 1.4[b]	-2.0 ± 1.0[b]	-1.9 ± 1.5[c]
Week 4	-1.8 ± 1.4[a]	-1.6 ± 1.6[d]	-1.3 ± 1.4[e]	-1.7 ± 1.0[f]	-1.8 ± 1.5[g]
Week 6	-1.8 ± 1.4[h]	-1.6 ± 1.6[b]	-1.7 ± 1.3[e]	-1.8 ± 0.8[e]	-1.9 ± 1.4[i]
Week 8	-1.6 ± 1.2[h]	-1.6 ± 1.7[d]	-1.6 ± 1.6[d]	-1.8 ± 0.9[e]	-1.7 ± 1.2[c]
Week 12	-1.6 ± 1.3[a]	-1.7 ± 1.7[j]	-1.5 ± 1.5[e]	-1.8 ± 0.9[d]	-1.7 ± 1.4[k]
Follow-up (Week 16)	-1.6 ± 1.3[h]	-1.7 ± 1.3[e]	-1.5 ± 1.4[f]	-1.9 ± 1.1	-1.6 ± 1.5[i]

[a]n=29; [b]n=15; [c]n= 26; [d]n=14; [e]n=13; [f]n=12; [g]n=27; [h]n=31; [i]n=28; [j]n=11; [k]n=22.

FAS, full analysis set; NAFLD, non-alcoholic fatty liver disease.

Supplementary Table 25. Pro-C3 concentration over time (baseline to follow-up; FAS).

Pro-C3, mean ± SD, ng/mL	Placebo (n=32)	BI 1467335			
		1 mg (n=16)	3 mg (n=16)	6 mg (n=16)	10 mg (n=32)
Baseline	16.6 ± 6.5[a]	20.6 ± 15.0	19.9 ± 14.2	14.9 ± 4.4	18.8 ± 12.4[a]
Week 4	16.7 ± 6.5	19.5 ± 10.2[b]	21.7 ± 18.0[c]	14.3 ± 5.4[c]	18.8 ± 14.4[a]
Week 12	16.2 ± 6.9	17.4 ± 6.6[d]	20.2 ± 18.9[b]	14.6 ± 8.1[b]	14.6 ± 5.1[e]
Follow-up (Week 16)	19.2 ± 16.1	18.3 ± 7.8[d]	17.5 ± 13.0[d]	15.1 ± 3.9	15.1 ± 5.7[f]

[a]n=31; [b]n=14; [c]n=15; [d]n=13; [e]n=26; [f]n=28.

FAS, full analysis set; Pro-C3, pro-peptide of type lll collagen.

Supplementary Table 26. Ethical Approval.

IRB/IEC	Protocol version approved	Date of approval
Comité voor medische ethiek,<br>Universitair Ziekenhuis (UZ) Antwerpen,<br>Wilrijkstraat 10,<br>2650 Edegem,<br>Belgium	2.0	26 June 2017
	3.0	5 July 2017
	4.0	4 April 2018
	5.0	31 August 2018
	6.0	22 October 2018
Institutional Review Board Services,<br>372 Hollandview Trail,<br>Aurora,<br>Ontario,<br>Canada	3.0	16 June 2017
	4.0	3 April 2018
ADVARRA Inc.,<br>6940 Columbia Gateway Drive,<br>Columbia,<br>Maryland	5.0	12 August 2018
	6.0	15 October 2018
University Health Network Research Ethics Board,<br>Hydro Building,<br>700 University Ave,<br>Toronto,<br>Canada	3.0	20 November 2017
	4.0	26 June 2018
	6.0*	4 January 2019
Conjoint Health Research Ethics Board (CHREB),<br>Mackimmie Library Tower,<br>2500 University Drive N.W,<br>T2N 1N4,<br>Calgary,<br>Alberta,<br>Canada	3.0	19 June 2017
	4.0	4 April 2018
	5.0	14 September 2018
	6.0	17 October 2018
Comité de Protection des Personnes (CPP) SUD-EST II,<br>Bâtiment Pinel,<br>Groupement Hospitalier Est,<br>52 Boulevard Pinel,<br>69003 LYON,<br>France	1.0	12 April 2017
	2.0	23 August 2017
	3.0	23 August 2017
	4.0	25 April 2018
	5.0	7 November 2018
	6.0	21 December 2018
Ethik-Kommission bei der Medizinischen,<br>Fakultät der Universität Würzburg,<br>Institut für Pharmakologie und Toxikologie,<br>Versbacher Straße 9,	2.0	30 August 2017
	3.0	20 September 2017
	4.0	2 May 2018

97078 Würzburg,<br>Germany	5.0	29 August 2018
	6.0	7 December 2018
Clinical Research Ethics Committee,<br>Lancaster Hall,<br>6 Little Hanover Street,<br>Cork,<br>County Cork,<br>Ireland	2.0	23 March 2017
	3.0	19 May 2017
	4.0	16 March 2018
	5.0	19 July 2018
	6.0	12 September 2018
Stichting Beoordeling Ethiek Biomedisch,<br>Stationsstraat 9,<br>Assen 9401 KV,<br>Netherlands	2.0	4 May 2017
	3.0	2 June 2017
	4.0	2 April 2018
	5.0	31 August 2018
	6.0	8 November 2018
Comité de ética de la Investigación con<br>Medicamentos, del Hospital Universitario La Paz<br>Paseo de la Castellana 261;<br>28046 Madrid,<br>Spain	3.0	10 August 2017
	4.0	12 April 2018
	6.0*	06 November 2018
South Central – Oxford A Research Ethics<br>Committee,<br>Bristol Research Ethics Committee Centre,<br>Whitefriars,<br>Lewins Mead,<br>Bristol BS1 2NT,<br>UK	2.0	23 March 2017
	3.0	19 May 2017
	4.0	16 March 2018
	5.0	19 July 2018
	6.0	12 September 2018
Quorum Review IRB,<br>1501 Fourth Avenue,<br>Seattle,<br>Washington 98101,<br>US	1.0	9 February 2017
	2.0	21 June 2017
	3.0	21 June 2017
	4.0	16 April 2018
	5.0	22 August 2018
	6.0	28 September 2018
Virginia Commonwealth University<br>Western IRB<br>1019 39th Avenue,<br>SE Puyallup,<br>Washington 98374,<br>US	3.0	2 November 2017
	4.0	29 November 2018
	5.0	29 November 2018
	6.0	29 November 2018
Duke University Health System IRB,	1.0	19 June 2017

Duke University Medical Center,<br>Hock Plaza,<br>2424 Erwin Road,<br>Durham,<br>North Carolina 27705,<br>US	3.0	17 August 2017
	4.0	27 April 2018
	5.0	24 September 2018
	6.0	01 February 2019
University of California San Diego,<br>UCSD Human Research Protection Program,<br>9500 Gilman Drive<br>La Jolla,<br>California 92093,<br>US	2.0	4 October 2017
	3.0	25 October 2017
	4.0	24 May 2018
	5.0	04 October 2018
	6.0	19 December 2018
Rutgers Robert Wood Johnson Medical School,<br>Western IRB,<br>1019 39th Avenue SE,<br>Puyallup,<br>Washington 98374-2115,<br>US	3.0	5 October 2017
	4.0	30 April 2018
	5.0	11 September 2018
	6.0	15 October 2018

*Global Protocol Version 5.0 and Version 6.0 were submitted together to IEC; as protocol Version 6.0 includes all the changes implemented at version 5.0, the IEC only performed one approval for version 6.0. IRB, Institutional Review Board; IEC, Independent Ethics Committee.

Supplementary figures

Supplementary Figure 2. Non-significant linear dose–response relationships of AST, AP, GGT and CK-18 total relative to baseline at Week 12 (PPS).

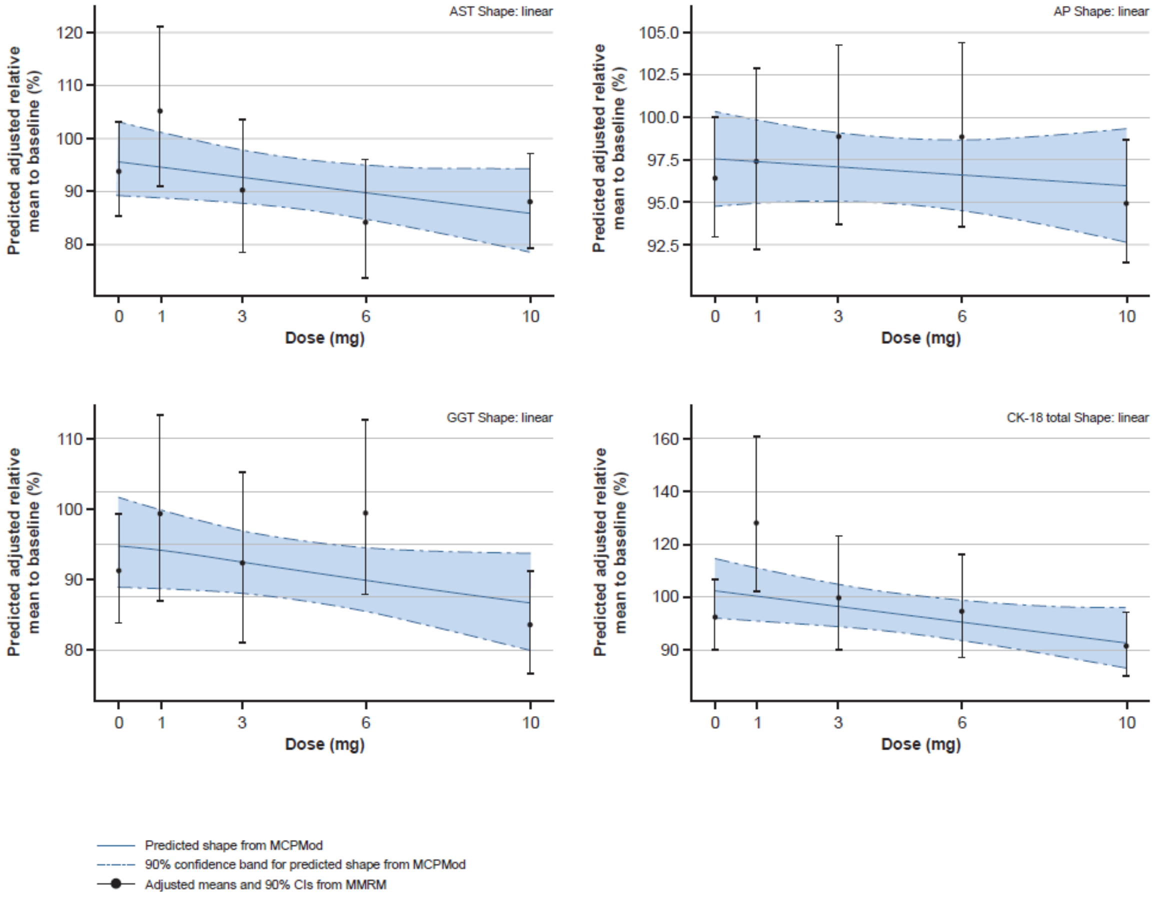

Error bars show MMRM-generated adjusted mean (least-square means) and two-sided 90% CI estimates based on the t-distribution for each dose (detailed in Supplementary Tables 2–5) relative to baseline at Week 12 for AST, AP, GGT and CK-18 total. Linear dose–response patterns (blue shapes) generated by the multiple contrast test MCPMod for each biomarker, show the predicted shape of the dose–response relationship. None of the models showed a statistically significant non-flat dose response (adjusted one-sided p-value >0.05). Linear model p-values: AST, 0.193; AP, 0.426; GGT, 0.201; CK-18 total, 0.0979 (exponential, logistic, quadratic and sigma Emax shapes are not shown, all p>0.05). Source data are provided as a Source Data file. AST, aspartate aminotransferase; AP, alkaline phosphatase; GGT, gamma-glutamyl transferase; CK-18 total, total cytokeratin 18; MCPMod; Multiple Comparison Procedure – Modelling; MMRM, mixed effects model for repeated measurements; PPS, per-protocol set.

Supplementary Figure 3. Placebo-corrected ALT, AST, AP, GGT, CK-18 caspase and CK-18 total relative to baseline MMRM results over time (PPS).

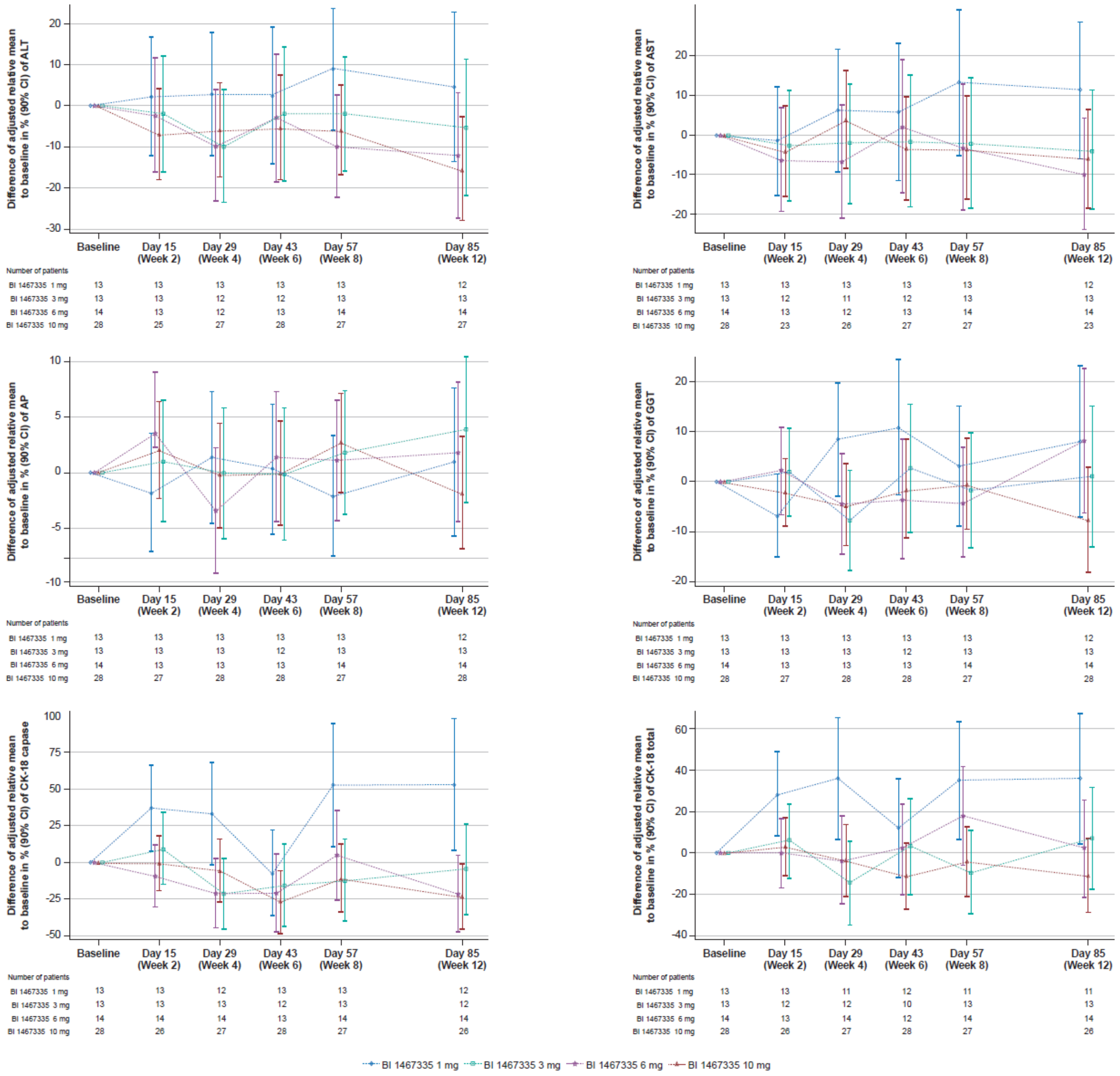

Source data are provided as a Source Data file. ALT, alanine aminotransferase; AP, alkaline phosphatase; AST, aspartate aminotransferase, CK-18 caspase, caspase-cleaved cytokeratin 18; CK-18 total, total cytokeratin 18; GGT, gamma-glutamyl transferase; MMRM, mixed effects model for repeated measurements; PPS, per-protocol set.

Supplementary Figure 4. Change of Pro-C3 from baseline versus ALT change from baseline over time (FAS).

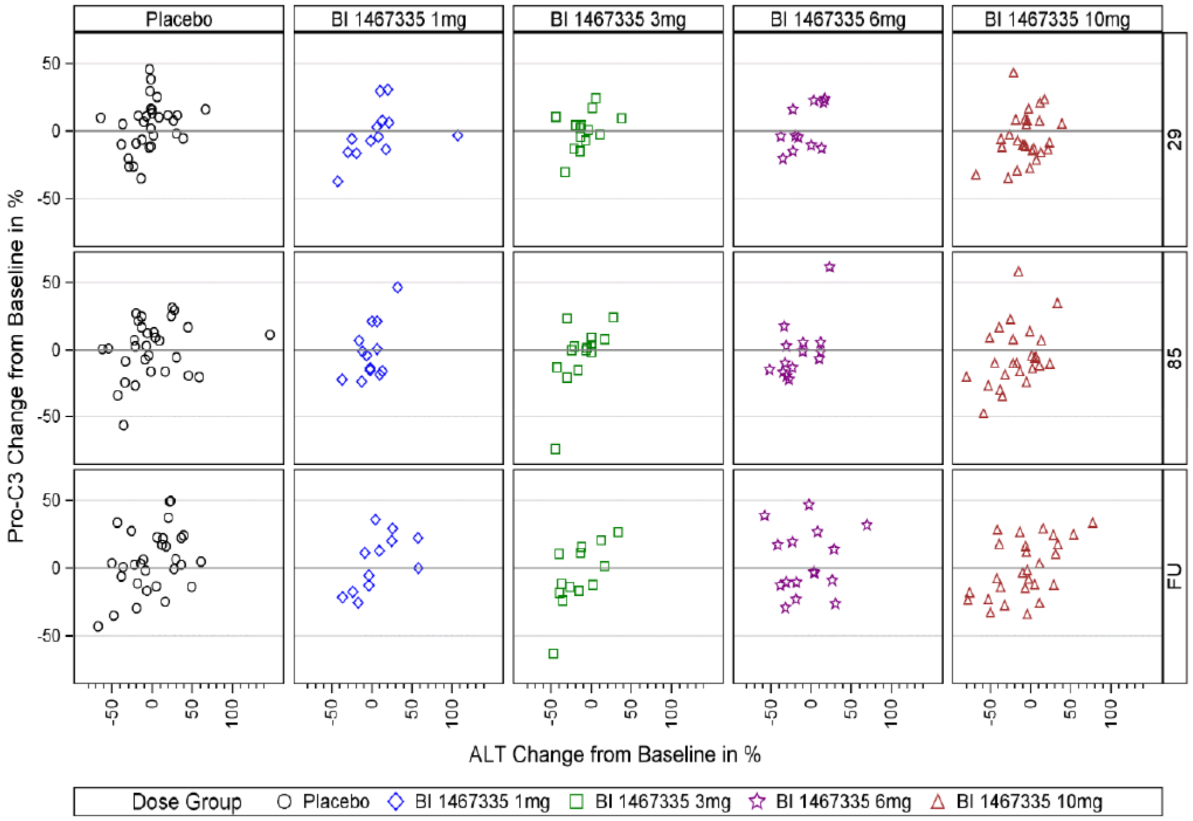

Post hoc analysis of patients with a baseline Pro-C3 value >15 ng/mL. Within the boxes each dot represents an individual patient. The x axis indicates the percentage change in ALT and the y axis represents the percentage change in Pro-C3. ALT, alanine aminotransferase; FAS, full analysis set; FU, follow-up; Pro-C3, pro-peptide of type III collagen. There was one patient with a Pro-C3 change from baseline of 232.09% and a respective ALT change from baseline of 200% at follow-up. This patient with extreme values is not shown on the figure.

Supplementary Figure 5. Change of Pro-C3 from baseline versus AST change from baseline over time (FAS).

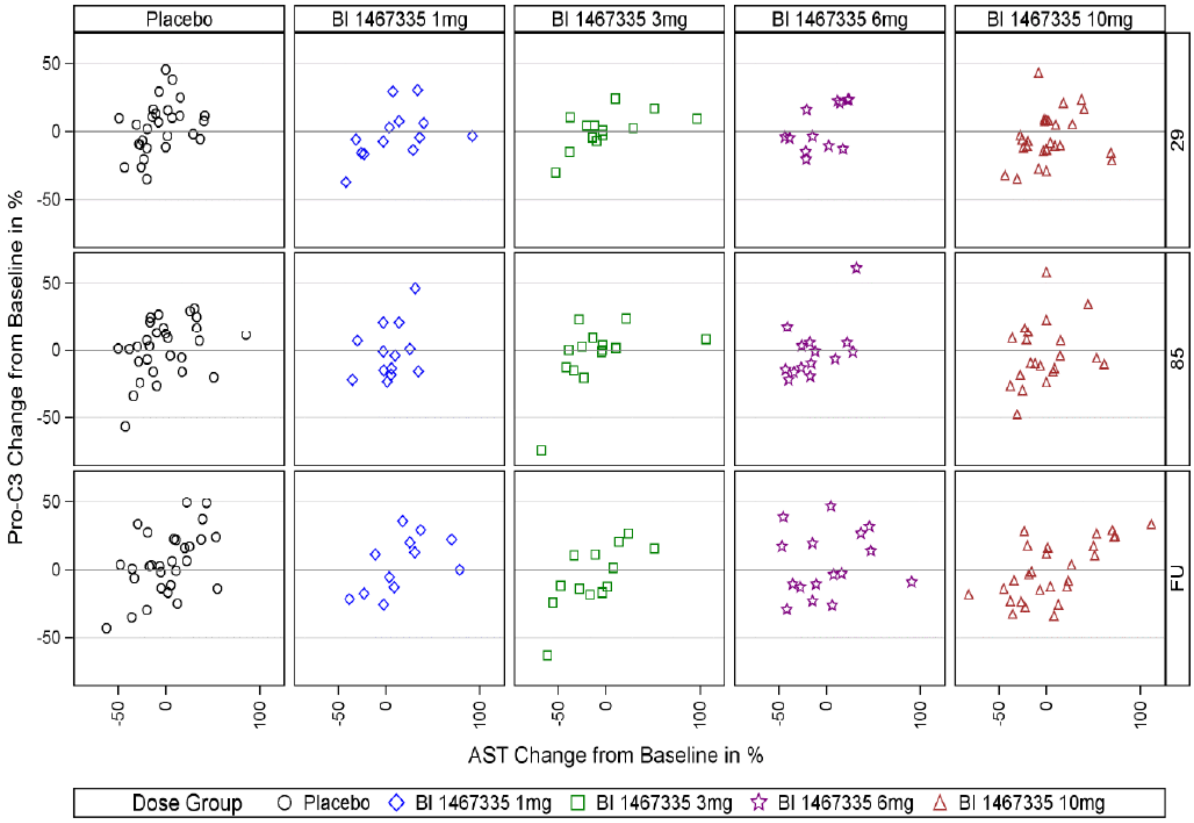

Post-hoc analysis of patients with a baseline Pro-C3 value >15 ng/mL. Within the boxes each dot represents an individual patient. The x axis indicates the percentage change in AST and the y axis represents the percentage change in Pro-C3. AST, aspartate aminotransferase; FAS, full analysis set; FU, follow-up; Pro-C3, pro-peptide of type III collagen. There was one patient with a Pro-C3 change from baseline of 232.09% and a respective AST change from baseline of 535.42% at follow-up. This patient with extreme values is not shown on the figure.

Supplementary Figure 6. Mean change (SD) of Pro-C3 from baseline over time (FAS).

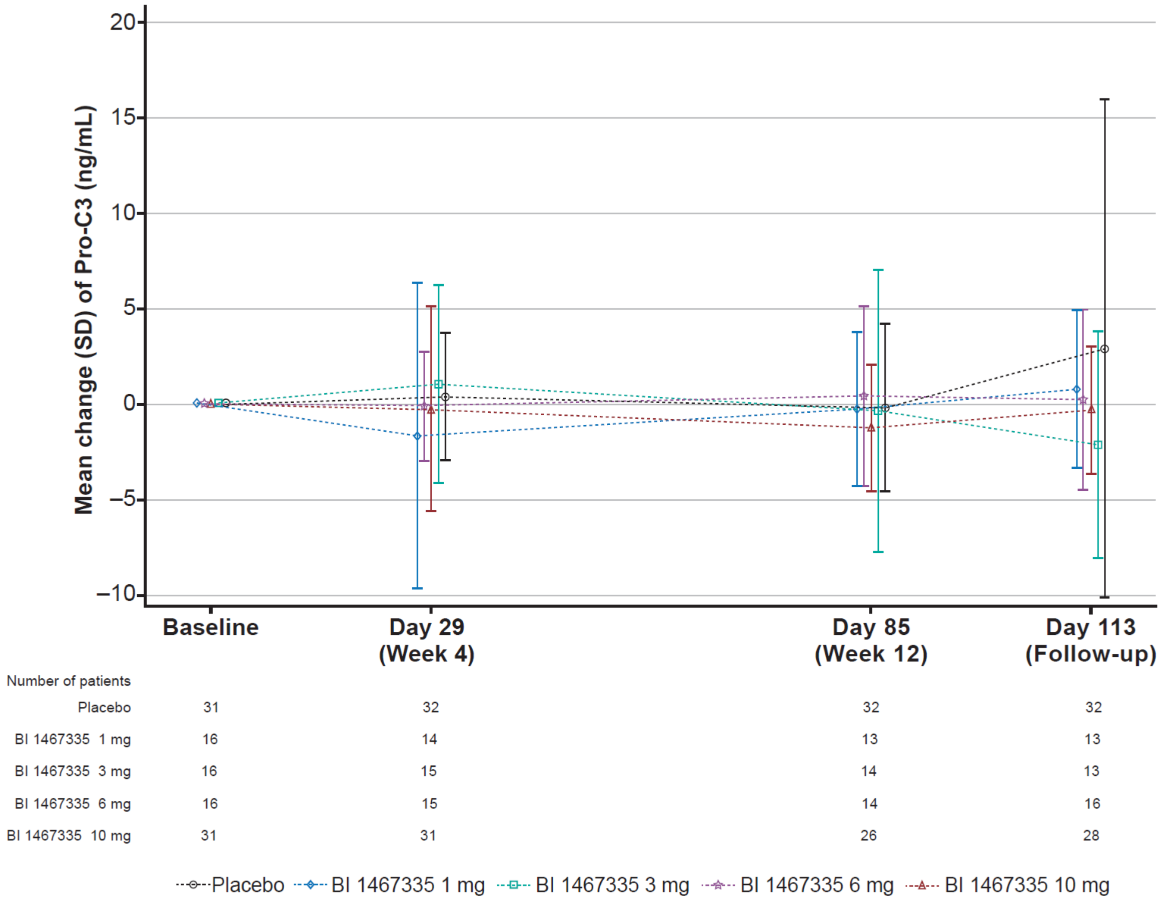

Source data are provided as a Source Data file. FAS, full analysis set; Pro-C3, pro-peptide of type III collagen.

Trial Collaborators

Full list of BI 1467335 NASH Phase IIa trial team collaborators

Manal Abdelmaelk[1], Raúl Andrade Bellido[2], Rodolphe Anty[3], Salvador Augustin[4], Alfred Barritt[5], Thomas Berg[6], David Bernstein[7], Maurizio Bonacini[8], Marc Bourlière[9], Stephen Congly[10], Lynsey Corless[11], Javier Crespo García[12], Jean Delwaide[13], Moises Diago[14], Joost Drenth[15], Magdy ElKhashab[16], Sven Francque[17], Juan Frias[18], Javier García Samaniego[19], Daniel Gaudet[20], Anja Geerts[21], Andreas Geier[22], Guido Gerken[23], Norman Gitlin[24], Shaun Greer[25], Karim Hamesch[26], Stephen Harrison[27], Tarek Hassanein[28], Jean Henrion[29], Peter Heymer[30], Gerard K. Hovingh[31], Stanley Hsia[18], Emmanuelle Huchet[32], Saumya Jayakumar[10], Kelly Kaita[33], Ger Koek[34], Michelle Lai[35], Anne Lamproye[12], Rohit Loomba[36], Kathryn Lucas[37], Paul Martin[38], Philippe Mathurin[39], Christophe Moreno[40], Frederick Nevens[41], Suzanne Norris[42], Jeffery Overcash[43], Keyur Patel[44], Alnoor Ramji[45], Dewkoemar Ramsoekh[46], Hendrik Reynaert[47], Manuel Romero[48], Julio Rosenstock[49], Ahmed Shoker[50], Nick Stern[51], Frank Tacke[26], Edward Tam[52], Christophe Van Steenkiste[53], Angel Vento[54], Philip N. Newsome[55,56], Arun J Sanyal[57], Guy Neff[58], Jörn M Schattenberg[59], Vlad Ratziu[60], Eric Lawitz[61]

Full list of collaborator affiliations

[1]Duke University Medical Centre, USA; [2]Servicio de Farmacología Clínica, Spain; [3]Hôpital de l'Archet 2, France; [4]Hospital Universitari Vall d'Hebron, Spain; [5]The University Hospital of North Carolina at Chapel Hill, USA; [6]Universitätsklinikum Leipzig AöR, Germany; [7]Northwell Health-Sandra Atlas Bass Canter for Liver Diseases, USA; [8]Quest Clinical Research, USA; [9]Hôpital Saint Joseph Service d'Hépato-Gastroentérologie, France; [10]University of Calgary, Canada; [11]Hull Royal Infirmary, Hull, UK; [12]Servicio Digestivo, Spain; [13]CHU de Liège - Domaine Universitaire du Sart Tilman, Belgium; [14]Servico de Hepatología, Spain; [15]Radboud Universitair Medisch Centrum, The Netherlands; [16]Toronto Liver Center, Canada; [17]Universitair Ziekenhuis Antwerpen – UZA, Belgium; [18]National Research Institute, USA; [19]Hospital Universitario La Paz, Spain; [20]Universitie de Montreal, Canada; [21]UZ Gent, Belgium; [22]Universitätsklinikum Würzburg, Germany; [23]Universitätsklinikum Essen AöR, Germany; [24]AGA, LLC, USA; [25]Manchester Royal Infirmary, UK; [26]Universitätsklinikum Aachen AöR, Germany; [27]Pinnacle Clinical Research, USA; [28]Southern California Research Center, USA; [29]Hopital de Jolimont-Lobbes Belgium; [30]Klinische Forschung Dresden GmbH, Germany; [31]Academisch Medisch Centrum (AMC), The Netherlands; [32]Clinique Medecine du Quartier Latin, Canada; [33]University of Manitoba, Canada; [34]Maastricht Universitair Medisch Centrum, The Netherlands; [35]Beth Israel Deaconess Medical Center, USA; [36]Altman Clinical and Translational Research Institute, USA; [37]Diabetes and Endocrinology Consultants, PC, USA; [38]University of Miami, USA; [39]Hôpital Claude Huriez, France; [40]Erasme University Hospital, Belgium; [41]Universitaire Ziekenhuizen Leuven, Belgium; [42]St James's Hospital, Ireland;

[43]eStudySite, USA; [44]Toronto General Hospital, Canada; [45]GIRI GI Research Institute, Canada; [46]VU Medisch Centrum, The Netherlands; [47]Universitair Ziekenhuis Brussels, Belgium; [48]Hospital Universitario Virgen del Rocio, Spain; [49]Dallas Diabetes and Endocrine Center, USA; [50]University of Saskatechewan, Canada; [51]Liverpool University Hospitals NHS Foundation Trust, UK; [52]Liver and Intestinal Research Centre, Canada; [53]A.Z. Maria Middelares, Belgium; [54]Genoma Research Group Inc, USA; [55]National Institute for Health Research, Birmingham Biomedical Research Centre at University Hospitals Birmingham NHS Foundation Trust, Birmingham, UK, [56]Centre for Liver & Gastrointestinal Research, Institute of Immunology and Immunotherapy, University of Birmingham, Birmingham, UK, [57]Virginia Commonwealth University, Richmond, VA, USA, [58]Covenant Research, Sarasota, FL, USA, [59]Metabolic Liver Research Program University Medical Center, Mainz, Germany, [60]Sorbonne Université, Institute of Cardiometabolism and Nutrition, Hospital Pitié-Salpêtrière, Paris, France; [61]Texas Liver Institute, University of Texas Health, San Antonio, TX, USA.

Supplementary Notes

Supplementary Note 1. The CONSORT checklist.

CONSORT 2010 checklist of information to include when reporting a randomised trial*

Section/Topic	Item No	Checklist item	Reported on page No
**Title and abstract**			
	1a	Identification as a randomised trial in the title	1
	1b	Structured summary of trial design, methods, results, and conclusions (for specific guidance see CONSORT for abstracts)	2
**Introduction**			
Background and objectives	2a	Scientific background and explanation of rationale	3–4
	2b	Specific objectives or hypotheses	4
**Methods**			
Trial design	3a	Description of trial design (such as parallel, factorial) including allocation ratio	11
	3b	Important changes to methods after trial commencement (such as eligibility criteria), with reasons	11, S6
Participants	4a	Eligibility criteria for participants	11
	4b	Settings and locations where the data were collected	11
Interventions	5	The interventions for each group with sufficient details to allow replication, including how and when they were actually administered	12
Outcomes	6a	Completely defined pre-specified primary and secondary outcome measures, including how and when they were assessed	12–13
	6b	Any changes to trial outcomes after the trial commenced, with reasons	11, S6

Sample size	7a	How sample size was determined	13
	7b	When applicable, explanation of any interim analyses and stopping guidelines	S3–S5
Randomisation:			
Sequence generation	8a	Method used to generate the random allocation sequence	11–12
	8b	Type of randomisation; details of any restriction (such as blocking and block size)	11–12
Allocation concealment mechanism	9	Mechanism used to implement the random allocation sequence (such as sequentially numbered containers), describing any steps taken to conceal the sequence until interventions were assigned	11–12
Implementation	10	Who generated the random allocation sequence, who enrolled participants, and who assigned participants to interventions	11–12
Blinding	11a	If done, who was blinded after assignment to interventions (for example, participants, care providers, those assessing outcomes) and how	11–12
	11b	If relevant, description of the similarity of interventions	N/A
Statistical methods	12a	Statistical methods used to compare groups for primary and secondary outcomes	14
	12b	Methods for additional analyses, such as subgroup analyses and adjusted analyses	14
**Results**			
Participant flow (a diagram is strongly recommended)	13a	For each group, the numbers of participants who were randomly assigned, received intended treatment, and were analysed for the primary outcome	5
	13b	For each group, losses and exclusions after randomisation, together with reasons	5
Recruitment	14a	Dates defining the periods of recruitment and follow-up	5
	14b	Why the trial ended or was stopped	N/A
Baseline data	15	A table showing baseline demographic and clinical characteristics for each group	20–21

Numbers analysed	16	For each group, number of participants (denominator) included in each analysis and whether the analysis was by original assigned groups	5
Outcomes and estimation	17a	For each primary and secondary outcome, results for each group, and the estimated effect size and its precision (such as 95% confidence interval)	5–7; 22–23; S7–S30
	17b	For binary outcomes, presentation of both absolute and relative effect sizes is recommended	N/A
Ancillary analyses	18	Results of any other analyses performed, including subgroup analyses and adjusted analyses, distinguishing pre-specified from exploratory	N/A
Harms	19	All important harms or unintended effects in each group (for specific guidance see CONSORT for harms)	7–8; 24–25
**Discussion**			
Limitations	20	Trial limitations, addressing sources of potential bias, imprecision, and, if relevant, multiplicity of analyses	9–10
Generalisability	21	Generalisability (external validity, applicability) of the trial findings	9–10
Interpretation	22	Interpretation consistent with results, balancing benefits and harms, and considering other relevant evidence	9–10
**Other information**			
Registration	23	Registration number and name of trial registry	1, 2, 11
Protocol	24	Where the full trial protocol can be accessed, if available	11
Funding	25	Sources of funding and other support (such as supply of drugs), role of funders	2,18

*We strongly recommend reading this statement in conjunction with the CONSORT 2010 Explanation and Elaboration for important clarifications on all the items. If relevant, we also recommend reading CONSORT extensions for cluster randomised trials, non-inferiority and equivalence trials, non-pharmacological treatments, herbal interventions, and pragmatic trials. Additional extensions are forthcoming: for those and for up to date references relevant to this checklist, see www.consort-statement.org

CLINICAL TRIAL PROTOCOL

**Document Number:**	**c08980589-06**
**EudraCT No.:**<br>**EU Trial No:**	2016-000499-83
**BI Trial No.:**	1386-0004
**BI Investigational Product(s):**	BI 1467335
**Title:**	A multi-centre, double-blind, parallel-group, randomised, placebo controlled phase II a study to investigate safety, tolerability, pharmacodynamics, and pharmacokinetics of different doses of orally administered BI 1467335 during a 12-week treatment period compared to placebo in patients with clinical evidence of NASH.
**Lay Title:**	Different doses of BI 1467335 compared to placebo in patients with clinical evidence of NASH
**Clinical Phase:**	IIa
**Trial Clinical Monitor:**	Phone:<br>Fax:
**Coordinating Investigator:**	Phone:<br>Fax:
**Status:**	**Final Protocol (Revised Protocol based on global amendment 5)**
**Version and Date:**	**Version 6.0** **Date: 12 Sep 2018**

Page 1 of 101

Proprietary confidential information.
© 2018 Boehringer Ingelheim International GmbH or one or more of its affiliated companies. All rights reserved.
This document may not - in full or in part - be passed on, reproduced, published or otherwise used without prior written permission.

Proprietary confidential information © 2018 Boehringer Ingelheim International GmbH or one or more of its

CLINICAL TRIAL PROTOCOL SYNOPSIS

**Name of company:**		**Boehringer Ingelheim**	
**Name of finished product:**		NA	
**Name of active ingredient:**		BI 1467335	
**Protocol date:** 02 Dec 2016	**Trial number:** 1386-0004		**Revision date:** 12 Sep 2018

**Title of trial:**	A multi-centre, double-blind, parallel-group, randomised, placebo controlled phase II a study to investigate safety, tolerability, pharmacodynamics, and pharmacokinetics of different doses of orally administered BI 1467335 during a 12-week treatment period compared to placebo in patients with clinical evidence of NASH.
**Coordinating Investigator:**	Phone:<br>Fax:
**Trial site(s):**	Multicentre Trial conducted in approximately 8 countries / 50 sites
**Clinical phase:**	IIa
**Objective(s):**	The primary objective of this study is the proof of mechanism and support of dose finding, together with the safety evaluation in patients with clinical evidence of NASH over different doses of BI 1467335 compared to placebo.
**Methodology:**	Placebo-controlled, double blind, randomised, parallel design
**No. of patients:**	comparison of 5 groups over 12 weeks of treatment.
**total entered:**	Approximately 108
**each treatment:**	Placebo n_1=28; 1mg BI 1467335 n_2=14; 3mg BI 1467335 n_3=14; 6mg BI 1467335 n_4=14; 10mg BI 1467335 n_5=28
**Diagnosis :**	Patients with clinical evidence for non-alcoholic-steato-hepatitis based on a) histological evidence or b) hepatic steatosis in combination with hepatic fibrosis.

Proprietary confidential information © 2018 Boehringer Ingelheim International GmbH or one or more of its

**Name of company:**		**Boehringer Ingelheim**	
**Name of finished product:**		NA	
**Name of active ingredient:**		BI 1467335	
**Protocol date:**<br>02 Dec 2016	**Trial number:**<br>1386-0004		**Revision date:**<br>12 Sep 2018

**Main criteria for inclusion:**	The population will include male and female patients over 18 years ofage with clinical evidence for NASH and ALT > 1.5 upper limit normal. Patients with a history of significant alcohol consumption or other forms of chronic liver disease (including liver cirrhosis) will be excluded.
**Test product(s):**	BI 1467335
**dose:**	1mg QD, 3mg QD, 6mg QD, 10mg QD
**mode of administration:**	Tablet, p.o.
**Comparator products:**	Placebo
**dose:**	Not applicable
**mode of administration:**	Tablet, p.o.
**Duration of treatment:**	12 weeks

Proprietary confidential information © 2018 Boehringer Ingelheim International GmbH or one or more of its

**Name of company:**		**Boehringer Ingelheim**	
**Name of finished product:**		NA	
**Name of active ingredient:**		BI 1467335	
**Protocol date:**<br>02 Dec 2016	**Trial number:**<br>1386-0004		**Revision date:**<br>12 Sep 2018

**Endpoints**	Primary Endpoint: Plasma AOC3 activity relative to baseline in %, at 24 h post dose, after 12 weeks of treatment<br>Secondary endpoints:<br>• number (%) of subjects with adverse reactions<br>• Relative ALT change from baseline after 12 weeks of treatment<br>• Relative AST change from baseline after 12 weeks of treatment<br>• Relative AP change from baseline after 12 weeks of treatment<br>• Relative γ-GT change from baseline after 12 weeks of treatment<br>• Relative caspase cleaved cytokeratin 18 (M30) change from baseline after 12 weeks of treatment<br>• Relative total cytokeratin 18 (M65) change from baselineafter 12 weeks of treatment
**Safety criteria:**	Adverse event reporting, vital signs and standard laboratory tests
**Statistical methods:**	For dose-response relationship analysis a non-linear 3-parametric model is used to fit the primary endpoint AOC3 activity relative to baseline, at 24 h post dose, after 12 weeks of treatment with either BI1467335 or placebo. The dose will be estimated where the fitted mean curve drops below 10%. The secondary biomarker endpoints including ALT, AST, AP, γ-GT, caspase cleaved and total CK18 are evaluated using a mixed effect model for repeated measurements (MMRM). The estimated dose effects at week 12 will then be analysed using the MCPMod approach to test if there is a non-flat dose response relationship between the different doses of BI 467335 and placebo.

Proprietary confidential information © 2018 Boehringer Ingelheim International GmbH or one or more of its

FLOW CHART 1

**Trial Period**	**Screening[2]**	**Treatment Period**								**Follow-up**
**Visit or phone call (☎)**	**1**	**2 Baseline**	**3**	**☎ 3A**	**4**	**5**	**6**	**☎ 6A**	**EOT/ ED[6]**	**FU**
**Study-Day**	-28 to -7	**1**	**15**	**27**	**29**	**43**	**57**	**83**	**85**	**EOT +28**
**Week**	**-4 to -1**		**2**		**4**	**6**	**8**		**12**	16
**Time window (in days)**			±3	-2	±3	±3	±3	-2	+3	±3
Patient information & informed consent signed (including informed consent for biobanking[1])	X									
Register Patient in IRT	X									
Randomisation (via IRT)		X								
Demographics	X									
Medical history / baselineconditions	X									
In-/exclusion criteria, incl. quantification of alcohol consumption	X	X[13]								
Clinical imaging (optional)[21]	X[21]									
Concomitant medications	X	X	X		X	X	X		X	X
Height (screening only) / weight / waist and hip circumference[17]	X	X	X		X	X	X		X	X
Vital signs	X	X	X		X	X	X		X	X
Physical examination	X	X	X[14]		X[14]	X[14]	X[14]		X	X
Resting ECG [12]	X	X	X		X	X	X		X	X
Adverse events	X	X	X	X	X	X	X	X	X	X
Pregnancy testing[10, 15]	Xs	Xu	Xu		Xu	Xu	Xu		Xu	Xu
IRT call[3]		X			X		X		X	
Dispense trial medication		X			X		X			
Collect study drug					X		X		X	
Check of medication compliance and dispense/ collect study drug diary [11]		X	X		X	X	X		X	
Telephone contact[16]				X				X		
Infection testing[19]	X									
Safety laboratory tests[15], biomarkers (except screening)	X[20]	X[9]	X		X[9]	X	X		X[9]	X[9]
Biomarker urine collection [22]		X	X		X	X	X		X	X
Sodium citrate coagulationcollection[23]		X	X		X		X		X	X
PK sampling[4]		X	X		X	X	X		X	
PG sampling[5]		X							X	
PD sampling[8]		X	X		X	X	X		X	X[18]
Blood samples Biobanking		X							X	

[1] Prior to any study related procedure, may also be done at an extra visit up to 2 weeks before V1.
[2] Screening visit to be performed -28 to -7 days before randomisation visit (V2).

Proprietary confidential information © 2018 Boehringer Ingelheim International GmbH or one or more of its affiliated companies

[3] At visits 2, 4 and 6, the respective IMP kit number has to be allocated to the patient via IRT. At Visit EOT/ED the IRT call is performed to close out the patient (Termination of medication).

or

DNA banking, blood samples will be collected at visit 2 and EOT/ED (for details please refer to section 5.5.4).

[6] Also to be completed for patients who are withdrawn or who have discontinued the trial early: in case of early termination, the EOT/ED Visit will be completed instead of the planned treatment period visit. This early discontinuation (ED) visit will include the same procedures as the normal EOT visit except PK, PG and PD sampling. Visit FU should be performed 28 days after the last study drug intake.

For patients who discontinue IMP but are willing to attend scheduled trial visits, only one PK sample and one PD blood sample for measurement of AOC3 activity should be taken and the actual time of the blood draw recorded. Please refer to section 6.2.2.

[8] PD blood samples for measurement of AOC3 activity and concentration. Please see Flow Charts 2, 3, 4 and table 10.1:1 for timing.

For patients who discontinue IMP but are willing to attend scheduled trial visits, only one PD blood sample for measurement of AOC3 activity should be taken and the actual time of the blood draw recorded. Please refer to section 6.2.2.

[10] Pregnancy Testing: Xs =serum testing; Xu= onsite urine testing; Serum pregnancy is done at screening and as a reflex when urine testing is positive.

[11] All patients will complete a study drug diary during the treatment phase to document drug intake and treatment compliance. The diary will be dispensed to the patients at Visit 2 and regularly checked by site-staff at the following visits. Please refer to section 6.2.2

[12] 12-lead ECGs will be recorded at the visits outlined in the flowchart for all patients approximately 90 min after study drug intake. At visits without drug administration ECGs should preferentially be performed prior blood sampling. For detailed timing please refer to Flow Charts 2, 3, 4. ECGs will be recorded after the patients have rested for at least 5 minutes in a supine position. The ECG at the screening visit is regarded as baseline. Please refer to section 5.3.4

[13] Confirm eligibility from Screening visit.

[14] Only symptom-derived physical examination required.

[15] At dosing visits: The respective procedure is to be performed and completed prior to study drug administration.

[17] Waist and hip circumference only at screening and EOT/ED. For detailed instruction how to measure waist and hip circumference see Section 5.3.1.1.

[18] For the timing of this blood sample please refer to table 10.1:1. Not applicable in case of early discontinuation.

[20] If the historic ALT > 1.25 ULN value is older than 3 months prior to screening, the ALT > 1.5xULN must be confirmed via two measurements at least 1 week apart within the screening period. Please refer to sections 3.3.2 and 6.2.1

[21] Clinical imaging (optional): only applicable for patients who don’t have the required clinical imaging results suggestive of NASH yet (no more than 3 years prior to screening, according to Inclusion criterion #1). **Not required if histological**

[23] One additional blood sample (sodium citrate coagulation tube) will be taken for platelet-rich plasma (PRP). Please refer to section 5.5.2.

Proprietary confidential information © 2018 Boehringer Ingelheim International GmbH or one or more of its affiliated companies

FLOW CHART 2: TIMING OF PROCEDURES*: VISIT 2 AND 4 (DAYS 1 AND 29):

Time relative to drug administration (h)[4]	-24	-0.5	0	0.25	0.5	1	1.5	2	3	6	8
Administer trial medication[1]			X								
PK blood sampling		X		X	X	X	X[3]	X	X	X	X
PK urine collection[2]	<-		->								
AOC 3 PD blood sampling[4]		X[4]			X	X		X	X	X	X

[1] To be done at the clinical site

[2] PK urine collection only to be done on days 28-29. Container should be given to the patient on day 1 (or day 15). Site to call patient on day 27 to remind of 24 h urine collection and study drug intake. Collected urine to be returned to the site on the next morning.

[3] ECG measurement to be performed directly prior to the PK blood sampling at 1.5h.

[4] AOC3 activity measurements at all indicated time points; in addition AOC3 concentration measurement at -0.5h only

FLOW CHART 3: TIMING OF PROCEDURES*: VISIT 3, 5 AND 6 (DAYS 15, 43 AND 57):

Time relative to drug administration(h)	-0.5	0	1	1.5
Administer trial medication[1]		X		
PK blood sampling	X		X	X[2]
AOC 3 PD blood sampling	X[3]			

[1] To be done at the clinical site.

[2] ECG measurement to be performed directly prior to the PK blood sampling at 1.5 h.

[3] AOC3 activity and concentration measurement.

Proprietary confidential information © 2018 Boehringer Ingelheim International GmbH or one or more of its affiliated companies

FLOW CHART 4: TIMING OF PROCEDURES*: EOT (DAY 85):

Time relative to drug administration (h) [5]	-0.5	0	0.25	0.5	1	1.5	2	3	6	8	24
Administer trial medication [1]		X									
PK blood sampling	X		X	X	X	X[3]	X	X	X	X	X
PK urine collection[2]		<-	-	-	-	-	-	-	-	-> <-	->
AOC 3 PD blood sampling[4]	X[4]			X	X		X	X	X	X	X

[1] To be done at the clinical site
[2] 24 h urine collection Collection of 0-8 h urine to be performed at the site; collection of 8-24 h urine to be performed by patients and to be returned to the site in the next morning; no overnight stay is required.
[3] ECG measurement to be performed directly prior to the PK blood sampling at 1.5h.
[4] AOC3 activity measurements at all indicated time points; in addition AOC3 concentration measurement at -0.5h only.
[5] Site to call patient on day 83 to remind of study drug intake on day 84.

Proprietary confidential information © 2018 Boehringer Ingelheim International GmbH or one or more of its affiliated companies

TABLE OF CONTENTS

CLINICAL TRIAL PROTOCOL 1
TITLE PAGE 1
CLINICAL TRIAL PROTOCOL SYNOPSIS 2
FLOW CHART 1 5
FLOW CHART 2: TIMING OF PROCEDURES*: VISIT 2 AND 4 (DAYS 1 AND 29): 7
FLOW CHART 3: TIMING OF PROCEDURES*: VISIT 3, 5 AND 6 (DAYS 15, 43 AND 57): 7
FLOW CHART 4: TIMING OF PROCEDURES*: EOT (DAY 85): 8
TABLE OF CONTENTS 9
ABBREVIATIONS 12
1. INTRODUCTION 15
1.1 MEDICAL BACKGROUND 15
1.2 DRUG PROFILE 16
2. RATIONALE, OBJECTIVES, AND BENEFIT - RISK ASSESSMENT 19
2.1 RATIONALE FOR PERFORMING THE TRIAL 19
2.2 TRIAL OBJECTIVES 19
2.3 BENEFIT - RISK ASSESSMENT 20
3. DESCRIPTION OF DESIGN AND TRIAL POPULATION 22
3.1 OVERALL TRIAL DESIGN AND PLAN 22
3.1.1 Administrative structure of the trial 23
3.2 DISCUSSION OF TRIAL DESIGN, INCLUDING THE CHOICE OF CONTROL GROUP(S) 23
3.3 SELECTION OF TRIAL POPULATION 23
3.3.1 Main diagnosis for trial entry 24
3.3.2 Inclusion criteria 24
3.3.3 Exclusion criteria 25
3.3.4 Removal of patients from therapy or assessments 27
3.3.4.1 Removal of individual patients 27
3.3.4.1.1 Removal of individual patients in case of increased liver enzymes 27
3.3.4.1.2 Further criteria for removal of individual patients 29
3.3.4.2 Discontinuation of the trial by the sponsor 29
4. TREATMENTS 30
4.1 INVESTIGATIONAL TREATMENTS 30
4.1.1 Identity of the Investigational Medicinal Products 30
4.1.3 Method of assigning patients to treatment groups 32
4.1.4 Drug assignment and administration of doses for each patient 32

Proprietary confidential information © 2018 Boehringer Ingelheim International GmbH or one or more of its affiliated companies

4.1.5 Blinding and procedures for unblinding....33
4.1.5.1 Blinding....33
4.1.5.2 Unblinding and breaking the code....33
4.1.6 Packaging, labelling, and re-supply....33
4.1.7 Storage conditions....34
4.1.8 Drug accountability....34
4.2 OTHER TREATMENTS, EMERGENCY PROCEDURES, RESTRICTIONS....35
4.2.1 Other treatments and emergency procedures....35
4.2.2 Restrictions, warnings and precautions....35

4.3 TREATMENT COMPLIANCE....37
5. VARIABLES AND THEIR ASSESSMENT....38
5.1 TRIAL ENDPOINTS....38
5.1.1 Primary Endpoint(s)....38
5.1.2 Secondary Endpoint(s)....38
....38
5.2 ASSESSMENT OF EFFICACY....38
5.3 ASSESSMENT OF SAFETY....39
5.3.1 Physical examination....39
5.3.1.1 Waist and hip circumference....39
5.3.1.2 Body weight....39
5.3.2 Vital Signs....39
5.3.3 Safety laboratory parameters.....40
5.3.4 Electrocardiogram....42
5.3.5 Assessment of adverse events....43
5.3.5.1 Definitions of AEs....43
5.3.5.2 Adverse event collection and reporting....46

5.5 ASSESSMENT OF BIOMARKER(S)....50

5.5.2 Biochemical and cellular biomarkers....51
5.5.3 Methods of sample collection....52

5.5.5 Appropriateness of Measurements....54

Proprietary confidential information © 2018 Boehringer Ingelheim International GmbH or one or more of its affiliated companies

6. INVESTIGATIONAL PLAN...55

6.1 VISIT SCHEDULE...55
6.2 DETAILS OF TRIAL PROCEDURES AT SELECTED VISITS 55
6.2.1 Screening...55
6.2.2 Treatment period 56
6.2.3 Follow Up Period and Trial Completion...58

7. STATISTICAL METHODS AND DETERMINATION OF SAMPLE SIZE 59

7.1 STATISTICAL DESIGN - MODEL 59
7.2 NULL AND ALTERNATIVE HYPOTHESES...59
7.3 PLANNED ANALYSES...60
7.3.1 Primary endpoint analyses...60
7.3.2 Secondary endpoint analyses 62
...63
7.3.4 Safety analyses...64

7.4 INTERIM ANALYSES 65
7.5 HANDLING OF MISSING DATA...65
7.6 RANDOMISATION 65
7.7 DETERMINATION OF SAMPLE SIZE 66

8. INFORMED CONSENT, TRIAL RECORDS, DATA PROTECTION, PUBLICATION POLICY...69

8.1 TRIAL APPROVAL, PATIENT INFORMATION, INFORMED CONSENT 69
8.2 DATA QUALITY ASSURANCE 70
8.3 RECORDS...70
8.3.1 Source documents 70
8.3.2 Direct access to source data and documents...71
8.3.3 Storage period of records 72
8.4 EXPEDITED REPORTING OF ADVERSE EVENTS 72
8.5 STATEMENT OF CONFIDENTIALITY AND PATIENT PRIVACY 72
8.6 TRIAL MILESTONES 72

9. REFERENCES...74

9.1 PUBLISHED REFERENCES...74

10. APPENDICES...78

10.1 PHARMACOKINETIC AND BIOMARKER SAMPLING TIME POINTS78

10.3 INCREASED LIVER ENZYMES PROCEDURE 82

Proprietary confidential information © 2018 Boehringer Ingelheim International GmbH or one or more of its affiliated companies

ABBREVIATIONS

$\%AUC_{tz-\infty}$	the percentage of $AUC_{0-\infty}$ obtained by extrapolation
λ_z	terminal rate constant in plasma
Ae_{t1-t2}	amount of analyte that is eliminated in urine from the time interval t_1 to t_2
A1At	Alpha-1 Antitrypsin
AE	Adverse Event
AESI	Adverse Event of Special Interest
AOC3	amine oxidase copper-containing 3
AUC	Area under the Curve
AUC_{0-24}	area under the concentration-time curve of the analyte in plasma over thetime interval from 0 to 24 hours after administration
$AUC_{0-\infty}$	area under the concentration-time curve of the analyte in plasma over thetime interval from 0 extrapolated to infinity
AUC_{0-tz}	area under the concentration-time curve of the analyte in plasma over thetime interval from 0 to the last quantifiable data point
AUC_{t1-t2}	area under the concentration-time curve of the analyte in plasma over thetime interval t_1 to t_2
b.i.d.	bis in die (twice daily dosing)
BIRDS	Boehringer Ingelheim Regulatory Documents for SubmissionBody
BMI	Mass Index
C_{pre}	predose concentration of the analyte in plasma immediately before administration
$C_{0.5}$	concentration of the analyte in plasma 0.5 hours after administration
C_1	concentration of the analyte in plasma 1 hour after administration
C_{max}	maximum measured concentration of the analyte in plasma after administration
CCDS	Company Core Data Sheet
CI	Confidence Interval
$CL_{R, t1-t2}$	renal clearance of the analyte in plasma from the time point $_{t1}$ to $_{t2}$
CL/F	apparent clearance of the analyte in the plasma after extravascular administration
CML	Local Clinical Monitor
CRA	Clinical Research Associate
CRF	Case Report Form
CTCAE	Common Terminology Criteria for Adverse EventsCTP Clinical Trial Protocol
CTR	Clinical Trial Report
D10	Smallest dose where 10 % AOC3 activity is reached
DILI	Drug Induced Liver Injury
DMC	Data Monitoring Committee
DNA	Deoxyribonucleic acid
ED	Early discontinuation
EDC	Electronic Data Capture
eGFR	Estimated Glomerular Filtration Rate
EOT	End of treatment
ePRO	Electronic Patient Reported Outcome

Proprietary confidential information © 2018 Boehringer Ingelheim International GmbH or one or more of its affiliated companies

EudraCT	European Clinical Trials Database
FAS	Full Analysis Set
FC	Flow Chart
fe_{t1-t2}	fraction of administered drug excreted unchanged in urine from time pointt$_1$ to t_2
FFA	Free Fatty Acids
GCP	Good Clinical Practice Homeostasis
HOMA	Model Assessment
HPC	Human Pharmacology Center
hsCRP	High sensitive C-reactive protein
IB	Investigator's Brochure
IEC	Independent Ethics Committee
IRB	Institutional Review Board
IRT	Interactive Response Technology
ISF	Investigator Site File
i.v.	intravenous
LoEE	List of Essential Element
MedDRA	Medical Dictionary for Drug Regulatory Activities
MCPMod	Multiple Comparison Procedures – Modelling
MMRM	mixed effect model for repeated measurements
MRE	Magnetic Resonance Elastography
MRI-PDFF	Magnetic Resonance Imaging of Proton Density Fat Fraction mean
MRT_{ex}	residence time of the analyte in the body after extravascular administration
MST	Medical Sub team
NASH	Non-alcoholic steatohepatitis
OPU	Operative Unit
PD	Pharmacodynamics
PK	Pharmacokinetics
p.o.	per os (oral)
PCC	Protocol Challenge Committee
PRP	platelet-rich plasma
q.d.	quaque die (once a day)
$R_{A,Cmax}$	accumulation ratio based on C_{max} after multiple doses versus C_{max} after thefirst dose
$R_{A,AUCt1-t2}$	accumulation ratio based on AUC_{t1-t2} after multiple doses versus AUC_{t1-t2}after the first dose
REP	Residual effect period, after the last dose of medication with measureabledrug levels or pharmacodynamic effects still likely to be present
SAE	Serious Adverse Event
s.c.	Subcutaneous System
SOC	Organ Class
SPC	Summary of Product Characteristics semi-
SSAO	carbazide-sensitive amine oxidase
t_{max}	time from dosing to maximum measured concentration of the analyte in plasma

Proprietary confidential information © 2018 Boehringer Ingelheim International GmbH or one or more of its affiliated companies

$t_{1/2}$	terminal half-life of the analyte in plasma
TCM	Trial Clinical Monitor
TDMAP	Trial Data Management and Analysis Plan
t.i.d.	ter in die (3 times a day)
TMF	Trial Master File
TMW	Trial Medical Writer
TSAP	Trial Statistical Analysis Plan
US	Ultrasound
V_z/F	apparent volume of distribution during the terminal phase after extravascular administration

Proprietary confidential information © 2018 Boehringer Ingelheim International GmbH or one or more of its affiliated companies

1. INTRODUCTION

1.1 MEDICAL BACKGROUND

Boehringer Ingelheim (BI) is developing BI 1467335 (formerly Pharmaxis PXS-4728A), an oral, small-molecule inhibitor of semi-carbazide-sensitive amine oxidase (SSAO) also known as vascular adhesion protein-1 (VAP-1) and amine oxidase copper-containing 3 (AOC3) for the indication non-alcoholic steatohepatitis (NASH).

NASH is characterized histologically by a high level of steatosis, ballooning of hepatocytes, and necroinflammation. AOC3 in liver sinusoidal endothelial cells is responsible for the firm adhesion and transmigration of leukocytes and for the propagation of the inflammatory environment in steatohepatitis. Fibrotic regions of NASH liver sections are strongly positive for AOC3 immune reactivity [R15-5697]. The associated generation of peroxide during the course of amine oxidation is known to activate quiescent stellate cells supporting the differentiation into myofibroblasts and fibrotic tissue generation. Recent findings with AOC3 knockout mice or animals treated with anti-AOC3 antibody displayed reduced hepatic inflammation and fibrosis development in several disease-related models of liver injury [R15 5365] and are supportive of this therapeutic concept. Therefore, targeting inhibiting AOC3 enzymatic activity might be beneficial for patients with steatohepatitis and fibrosis in order to reduce the recruitment of leukocytes into the liver and reduce cytokine and oxygen stress dependent hepatocyte damage and activation of hepatic stellate cells.

With a prevalence of about 20–30% in the general population of Western countries, Non-Alcoholic-Fatty-Liver Disease (NAFLD) is rapidly becoming the most common liver disease worldwide [R15-5365]. While simple hepatic steatosis can have a benign non-progressive course, about 40% of patients with NASH progress [R16-5301].As the disease progresses, significant fibrosis develops in 37-41% of subjects within 15 years. In the United States, NASH is believed to be the most common cause of liver cirrhosis [R15-6070], which is estimated to be the 12th leading cause of death in the US, according to the National Institute of Health [R15-6057]. Patients with NASH are also at increased risk of hepatocellular carcinoma (HCC), even in the absence of cirrhosis [R15-5365]. By 2023, about 13 million patients are projected to have NASH with advanced stages (i.e. ≥ stage 3) of fibrosis (of those, 2.9 million in the US, 3.5 million in EU5, 5 million in China). The risk of liver-related death in Western patients with NASH ranges from 10% over 13.7 years to 18% over 18.5 years [P13-02280].

To date, no approved therapy for liver fibrosis or effective disease modifying regimen for NASH is available. The current standard of care for NASH is weight loss through diet and exercise to improve insulin resistance and lower fat mass, which is a clinically challenging goal to achieve and therefore only affects a minor proportion of the patients [R15-6044]. Persistent inflammation in response to liver injury is the critical factor that drives progression to fibrosis, cirrhosis, and hepatocellular carcinoma [R15-5365]. Inflammation at any site, including the liver, is the result of an accumulation of leukocytes organized into an inflammatory infiltrate. For this to occur, leukocytes must be recruited from the circulation by interactions with the endothelium and positioned within the tissue [R15-6046]. A key protein in promoting the recruitment of leukocytes in liver tissue is AOC3, which is constitutively expressed on human hepatic endothelium. BI 1467335 is an irreversible AOC3 inhibitor with anti-inflammatory and anti-oxidative stress activities that is currently under

Proprietary confidential information © 2018 Boehringer Ingelheim International GmbH or one or more of its affiliated companies

development for the treatment of chronic liver disease secondary to Non-alcoholic Steatohepatitis (NASH).

1.2 DRUG PROFILE

BI 1467335 is an AOC3 inhibitor that is being developed for symptomatic treatment of NASH. In humans, NASH is characterized by steatohepatitis, lipotoxicity, necroinflammation, stellate cell differentiation, myofibroblast activation and fibrosis. The potential role of AOC3 in NASH warrants the development of the irreversible AOC3 inhibitor BI1467335 for the treatment of patients with NASH.

Proprietary confidential information © 2018 Boehringer Ingelheim International GmbH or one or more of its affiliated companies

Proprietary confidential information © 2018 Boehringer Ingelheim International GmbH or one or more of its affiliated companies

Proprietary confidential information © 2018 Boehringer Ingelheim International GmbH or one or more of its affiliated companies

2. RATIONALE, OBJECTIVES, AND BENEFIT - RISK ASSESSMENT

2.1 RATIONALE FOR PERFORMING THE TRIAL

Increased plasma levels of ALT in NASH patients are a consequence of the lipotoxicity-induced necroinflammation of the parenchymal liver tissue. Soluble plasma AOC3 (sAOC3) activity is positively correlated with a variety of diseases including atherosclerosis, liver and kidney fibrosis, and septic shock, thereby indicating a potential pivotal role in various forms of inflammation (R15-5801, R15-5774, R15-5773, R15-5766, R15-5767, P15-12198, R15-5771, R15-5772, R15-5802). Inhibition of AOC3, therefore, has the potential to reduce oxidative stress and hepatic inflammation in steatohepatitis, and to improve fibrosis resolution in patients with NASH and ultimately resulting in reduced liver cell damage, thereby reducing elevated plasma ALT levels. This first study in NASH patients is performed to support the proof of mechanism and to allow further insights into the dose-response relationship of BI 1467335.

2.2 TRIAL OBJECTIVES

The primary objective of this study is the proof of mechanism and support of dose finding, together with the safety evaluation in patients with clinical evidence of NASH.
This proof of mechanism study will explore the inhibition of AOC3 activity (% of baseline) after 12 weeks treatment as the primary endpoint.

. This will include the effect of BI 1467335 on reduction of secondary biomarker endpoints (ALT, AST, AP, γ-GT and CK18 fragments).
Safety will be assessed throughout the study to provide key information regarding the use of BI 1467335 in patients with NASH.

The proof of clinical mechanism will be achieved through primary endpoint comparison (inhibition of AOC3 activity (% of baseline)) of the four BI 1467335 doses (1mg QD, 3mg QD, 6mg QD, 10mg QD) and placebo. To this end, the dose-response relationship of the primary endpoint will be analysed by means of non-linear regression.

For the secondary endpoints ALT, AST, γ-GT, AP, caspase cleaved cytokeratin 18 and total cytokeratin 18 a non-flat dose response relationship between the BI doses and placebo will be tested using the multiple comparison procedures and modelling (MCPmod) approach [R10-1424].

Proprietary confidential information © 2018 Boehringer Ingelheim International GmbH or one or more of its affiliated companies

2.3 BENEFIT - RISK ASSESSMENT

Proprietary confidential information © 2018 Boehringer Ingelheim International GmbH or one or more of its affiliated companies

Proprietary confidential information © 2018 Boehringer Ingelheim International GmbH or one or more of its affiliated companies

3. DESCRIPTION OF DESIGN AND TRIAL POPULATION

3.1 OVERALL TRIAL DESIGN AND PLAN

This is a 12-week, multi-centre, randomised, double blind, placebo-controlled, parallel-group comparison in patients with NASH. In total, 108 patients who meet the entry criteria are planned to be randomised in this trial. All eligible patients will be instructed at every visit (including screening) to adhere to their normal dietary and exercise regimen from screening until safety follow-up visit (see inclusion criterion #5 for details). Eligible patients will be randomised to the 12 week double-blind treatment period at Visit 2 and will be assigned to one of the 5 treatment groups (placebo QD, BI 1467335 1 mg QD, BI 1467335 3 mg QD, BI 1467335 6 mg QD or BI 1467335 10 mg QD; each patient will receive one active treatment and/or placebos to match the alternative active treatment). The randomised treatment will be double blind. After the end of the double-blind treatment period, patients will be followed up for additional 4 weeks without study medication. Safety will be evaluated at each visit until end of the observational period which is 28 days after end of treatment or for an appropriately longer time in case of unresolved adverse events.

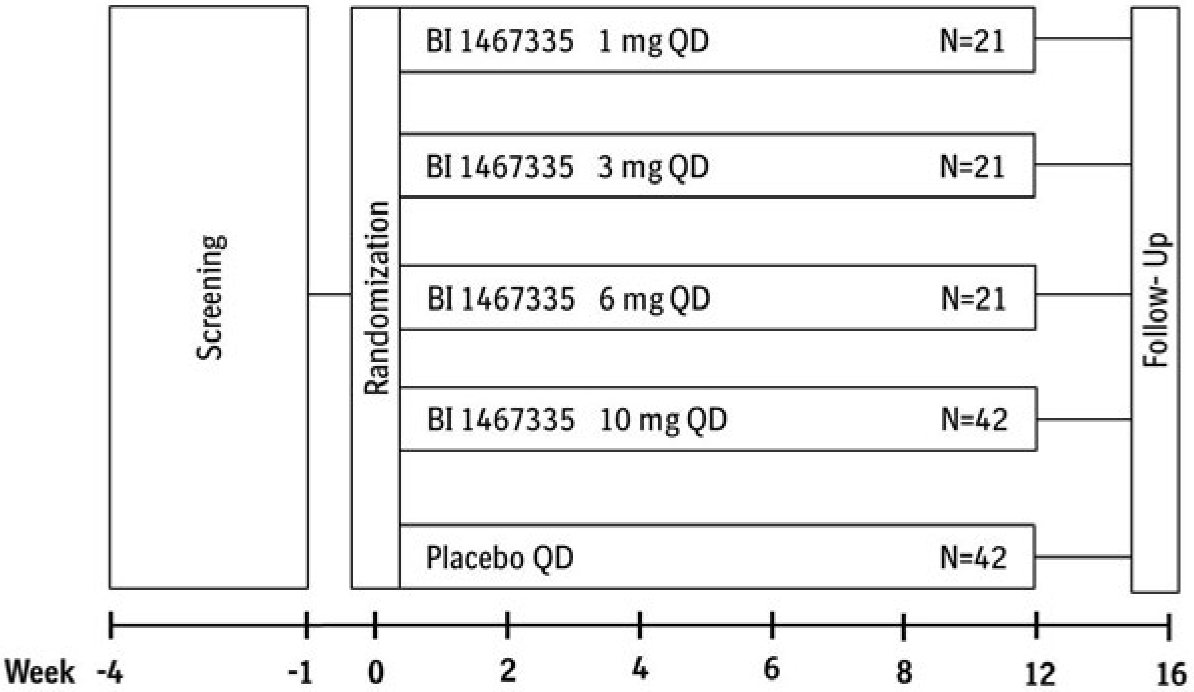

Figure 3.1: 1 Trial Design

Proprietary confidential information © 2018 Boehringer Ingelheim International GmbH or one or more of its affiliated companies

3.1.1 Administrative structure of the trial

The trial is sponsored by Boehringer Ingelheim (BI).

A Coordinating Investigator is responsible to coordinate Investigators at different centres participating in this multicentre trial. Tasks and responsibilities are defined in a contract. Relevant documentation on the participating (Principal) Investigators and other important participants, including their curricula vitae, will be filed in ISF.

Boehringer Ingelheim has appointed a Trial Clinical Monitor, responsible for coordinating all required activities, in order to

- manage the trial in accordance with applicable regulations and internal SOPs,
- direct the clinical trial team in the preparation, conduct, and reporting of the trial,
- ensure appropriate training and information of local clinical monitors (CML), Clinical Research Associates (CRAs), and Investigators of participating countries.

The organisation of the trial in the participating countries will be performed by the respective local or regional BI-organisation (Operating Unit, OPU) in accordance with applicable regulations and internal SOPs, or by a Contract Research Organisation (CRO) with which the responsibilities and tasks will have been agreed and a written contract filed before initiation of the clinical trial.
Data Management and Statistical Evaluation will be done by BI according to BI SOPs.

Tasks and functions assigned in order to organise, manage, and evaluate the trial are defined according to BI SOPs. A list of responsible persons and relevant local information can be found in the ISF.
Details of the trial supplies including responsible institutions are given in Section 4 of this protocol.
A central laboratory service and vendors for ECG and IRT (Interactive Response Technology) will be used in this trial. Details will be provided in the applicable manuals, available in the ISF.

3.2 DISCUSSION OF TRIAL DESIGN, INCLUDING THE CHOICE OF CONTROL GROUP(S)

A randomised, double blind, placebo- controlled design is chosen for this trial in order to evaluate safety, pharmacodynamics, and PK of BI 1467335 in patients with clinical evidence of NASH.
A placebo control arm was chosen to be included in order to evaluate the absolute effects of BI 1467335 on safety and tolerability. This is acceptable as no standard treatment is available.

3.3 SELECTION OF TRIAL POPULATION

In total, 108 patients with clinical evidence of NASH will be randomised to one of the five treatment arms. Approximately 50 sites in multiple countries are planned. Recruitment will be competitive. Patients who discontinue following randomisation will not be replaced and

Proprietary confidential information © 2018 Boehringer Ingelheim International GmbH or one or more of its affiliated companies

may not be re-enrolled at a later date. A record is kept of all patients failing to complete all trial visits and their reasons for discontinuation. Permission to randomise more than 20 patients per site must be obtained from the TCM at Boehringer Ingelheim. This will only be allowed after a careful review of the enrolment status.

A log of all patients enrolled into the trial (i.e. who have signed informed consent) will be maintained in the Investigator Site File (ISF) at the investigational site irrespective of whether they have been treated with investigational drug or not.

3.3.1 Main diagnosis for trial entry

The study will include patients with clinical evidence of NASH. This is defined as increase in ALT and diagnosis of NASH (histological evidence of NASH or presence of hepatic steatosis in combination with liver fibrosis (see inclusion criterion #1 for details) within 3 years prior to screening).

Please refer to section 8.3.1 (Source Documents) for the documentation requirements pertaining to the in- and exclusion criteria.

3.3.2 Inclusion criteria

1. Clinical evidence of NASH defined as
   a. histological evidence of NASH (no more than 3 years prior to screening)

   OR

   b. clinical imaging results suggestive of NASH (no more than 3 years prior to screening OR within the screening phase, imaging procedures performed as per local standard)
   i. evidence of hepatic steatosis >5% measured by the MRI-PDFF or assessed as steatosis (raised echogenicity of the liver parenchyma) by ultrasound

   AND

   ii. evidence of liver fibrosis defined as mean stiffness > 3.64 kPa as measured by the MRE protocol or mean stiffness > 7.2 kPa as measured by ultrasound based transient elastography (Fibroscan®)

2. Increased ALT defined as
   a. ALT >1.5 ULN at screening and ALT >1.25 ULN in a local lab within 1 week to 3 months prior screening
   OR
   b. Historic ALT > 1.25 ULN more than 3 months prior to screening and two consecutive ALT > 1.5xULN must be confirmed at least 1 week apart within the screening period

Proprietary confidential information © 2018 Boehringer Ingelheim International GmbH or one or more of its affiliated companies

3. Age ≥ 18 and ≤75 years at screening

4. BMI ≥25kg/m^2 and <45kg/m^2 at screening

5. Stable body weight defined as less than 5% change in body weight in the 3 months prior to screening while being treated with the standard of care and not treated with anti-obesity medication at screening.

6. Treatment with Antidiabetic concomitant medication including any insulin regimen needs to be stable for 3 months, and treatment with vitamin E needs to be stable for 6 months prior to informed consent and expected to be stable throughout the trial. All other concomitant medication has to be stable for at least 4 weeks prior screening. Concomitant medications taken to treat acute conditions (e.g. headache, sinusitis) for a short period (< 7 days) are permissible, if not otherwise prohibited. For restricted medications please refer to section 4.2.2.1.

7. For female patients: Women of childbearing potential* can be randomized after a negative pregnancy test and under adequate contraception with two methods, of which at least one is highly effective, during the trial.

   * A woman is considered of childbearing potential (WOCBP), i.e. fertile, following menarche and until becoming post-menopausal unless permanently sterile. Permanent sterilisation methods include hysterectomy, bilateral salpingectomy and bilateral oophorectomy. Tubal ligation is NOT a method of permanent sterilisation. A postmenopausal state is defined as no menses for 12 months without an alternative medical cause.

8. Signed and dated written informed consent in accordance with GCP and local legislation prior to admission to the trial.

3.3.3 Exclusion criteria

1. Current or history of significant alcohol consumption (defined as intake of >210g/week in males and >140g/week in females on average over a consecutive period of more than 3 months) or inability to reliably quantify alcohol consumption based on investigator judgement.

2. Prior participation in an interventional NASH trial 6 months before baseline or 5 times halflife of the investigational drug, whichever is longer.

3. Prior or planned bariatric surgery during study conduct, except gastric-band surgery more than 2 years prior to screening (including adjustments) with a stable body weight within the last 12 months.

4. Use of drugs historically associated with liver injury, hepatic steatosis or steatohepatitis in the 4 weeks prior to screening; please refer to section 4.2.2.

Proprietary confidential information © 2018 Boehringer Ingelheim International GmbH or one or more of its affiliated companies

5. History of liver cirrhosis (fibrosis stage 4), or hepatic decompensation (e.g. ascites, hepatic encephalopathy, variceal bleeding, etc.) or history of other forms of chronic liver disease (for example Hepatitis B, Hepatitis C, autoimmune liver disease, primary biliary sclerosis, primary sclerosing cholangitis, Wilsons disease, hemochromatosis, A1At deficiency, history of liver transplantation).

6. Active known chronic or relevant acute infections, such as HIV (Human Immunodeficiency Virus), \viral hepatitis, or tuberculosis. QuantiFERON® TB test and HBs Ag test will be performed during screening. Patients with a positive test result may participate in the study if further work up (according to local practice/guidelines) establishes conclusively that the patient has no evidence of active infection.

7. Solid liver lesions other than haemangiomas.
   a. Suspicion or diagnosis or history of hepatocellular carcinoma (HCC)

8. eGFR <60ml/min/1.73m^2 at screening (CKD-EPI formula).

9. ALT >5.0 ULN at screening.

10. Platelet count < 150.000/µL

11. Bilirubin level > ULN (except for known Gilbert´s disease with a conjugated bilirubin of < 0.3 mg/dL))

12. Uncontrolled diabetes defined as an HbA1c ≥9.5% in the 3 months prior to or at screening.

13. Diagnosis of a serious or unstable disease including hepatic (other than NASH), renal, gastroenterologic, respiratory, cardiovascular (including ischemic heart disease), endocrinologic, neurologic , psychiatric, immunologic, or hematologic disease and other conditions that, in the clinical judgment of the investigator, are likely to interfere with the analyses of safety and efficacy in this study. Patients with an expected life expectancy of less than 2 years are also excluded.

14. Major surgery (major according to the investigator’s assessment) performed within 12 weeks prior to randomisation or planned during study conduct, e.g. hip replacement.

15. Any documented active or suspected malignancy or history of malignancy within 5 years prior to screening, except appropriately treated basal cell carcinoma of the skin or in situ carcinoma of uterine cervix.

16. Patients who must or wish to continue the intake of restricted medications (see section 4.2.2.1) or any drug considered likely to interfere with the safe conduct of the trial.

17. Previous randomisation in this trial.

Proprietary confidential information © 2018 Boehringer Ingelheim International GmbH or one or more of its affiliated companies

18. Currently enrolled in another investigational device or drug study, or less than 30 days since ending another investigational device or drug study(s), or receiving other investigational treatment(s).

19. Chronic drug abuse or any condition that, in the investigator's opinion, makes them an unreliable study subject or unlikely to complete the trial.

20. Women who are pregnant, nursing, or who plan to become pregnant while in the trial.

21. Patients with Wolff-Parkinson-White Syndrome, baseline QTc > 450 ms, family history of long QT, or on medication prolonging QT time at screening or planned initiation during the trial.

22. Any other clinical condition that, in the opinion of the investigator, would jeopardize patient safety while participating in this clinical trial.

3.3.4 Removal of patients from therapy or assessments

3.3.4.1 Removal of individual patients

An excessive withdrawal rate can have a severe negative impact on the scientific value of the trial. Every effort should be made to keep patients in the trial as scheduled. This includes careful patient selection and appropriate explanation of the trial requirements and procedures prior to enrolment as well as an explanation of the consequences of premature withdrawal.

An individual patient is to be withdrawn from trial treatment if:

- The patient withdraws consent for trial treatment or trial participation, without the need to justify the decision.
- The patient needs to take concomitant drugs that interfere with the investigational product or other trial medication 4.2.2,
- The patient can no longer be treated with trial medication for other medical reasons (such as surgery, adverse events, other diseases, or pregnancy)
- The patient has repeatedly shown to be non-compliant with important trial procedures and, in the opinion of both, the investigator and sponsor representative, is not willing or able to stick to the trial requirements in the future.

3.3.4.1.1 Removal of individual patients in case of increased liver enzymes

In addition, study-specific procedures have been defined in case of increased liver enzymes (AST, ALT and total bilirubin) after randomisation as outlined below [R16-5309].
Please also refer to section 10.3.

Proprietary confidential information © 2018 Boehringer Ingelheim International GmbH or one or more of its affiliated companies

Treatment should be temporarily interrupted and the increased laboratory values need to be confirmed by a second measurement (i.e. the patient is informed to stop IMP and to come for an additional unscheduled visit within 48 hours of initial laboratory hepatic injury alert / notification or as soon as possible if timelines cannot be met). Other causes for liver injury should be excluded.

1) **If the values are confirmed or even further increased, IMP will be permanently stopped and the patient is withdrawn from treatment** and will be monitored closely on a weekly basis until resolution or stabilization in the following cases:

AST is normal at baseline and:

- If AST is more than 8x ULN
- If AST is 5-8x ULN for more than 2 weeks

ALT and/or AST are abnormal at baseline and:

- If ALT or AST is more than 8x baseline OR > 500 U/L absolute value, whichever is lower.

After resolution or stabilization the patient is encouraged to continue in the study and complete all visits. If the patient is not willing to continue in the study, the patient should complete the withdrawal- and follow-up protocol study procedures.

2) **If the values are confirmed, IMP will stay interrupted and the patient will be monitored closely** until resolution or stabilization in the following cases:

AST is normal at baseline and:

- If AST is 3x – 8x ULN AND total bilirubin is > 2 mg/dL
- If AST is 3x – 8x ULN OR total bilirubin is > 2 mg/dL
  AND the patient is presenting clinical symptoms of hepatic injury as e.g. encephalopathy, nausea, vomiting, pruritus, severe fatigue, etc.

ALT and AST are abnormal at baseline and:

- If ALT or AST is 5x – 8x baseline OR > 300 U/L absolute value (whichever is lower)
  AND total bilirubin is > 2 mg/dL
- If ALT or AST is 5x – 8x baseline OR > 300 U/L absolute value (whichever is lower) OR total bilirubin is > 2 mg/dL
  AND the patient is presenting clinical symptoms of hepatic injury as e.g. encephalopathy, nausea, vomiting, pruritus, severe fatigue, etc.

If the values return to the baseline value (Visit 2), a re-start of the IMP can be considered after discussion with the sponsor. After re-start of IMP the patient should be monitored on a weekly basis until EOT. In the case the initially elevated liver enzyme, which lead to interruption,

Proprietary confidential information © 2018 Boehringer Ingelheim International GmbH or one or more of its affiliated companies

increases again more than 2x baseline, the IMP has to be discontinued permanently.

Given the patient's agreement, the patient will continue the study, or otherwise should be encouraged to undergo the procedures for early treatment discontinuation and follow up as outlined in the Flow Chart (FC) and section 6.2.3.

3.3.4.1.2 Further criteria for removal of individual patients

If a patient becomes pregnant during the trial, the study medication needs to be discontinued, and the patient will complete EOT- and Follow-up Visit procedures. The patient will be followed up until birth or otherwise termination of the pregnancy.

If a patient develops QTc > 500 ms during the trial, the study medication needs to be discontinued, and the patient will complete EOT- and Follow-up Visit procedures.

For all patients the reason for withdrawal (e.g. adverse events) must be recorded in the CRF. These data will be included in the trial database and reported.

Patients who discontinue the trial after receiving the first dose of study medication at visit 2 will not be replaced.

3.3.4.2 Discontinuation of the trial by the sponsor

Boehringer Ingelheim reserves the right to discontinue the trial overall or at a particular trial site at any time for the following reasons:

1. Failure to meet expected enrolment goals overall or at a particular trial site
2. Emergence of any efficacy/safety information invalidating the earlier positive benefit-risk-assessment that could significantly affect the continuation of the trial
3. Violation of GCP, the CTP, or the contract disturbing the appropriate conduct of the trial

The Investigator / the trial site will be reimbursed for reasonable expenses incurred in case of trial termination (except in case of the third reason).

Proprietary confidential information © 2018 Boehringer Ingelheim International GmbH or one or more of its affiliated companies

4. TREATMENTS

4.1 INVESTIGATIONAL TREATMENTS

Multiple doses of BI 1467335 and/or Placebo to match BI 1467335 will be administered. All products will be supplied by Boehringer Ingelheim.

4.1.1 Identity of the Investigational Medicinal Products

Table 4.1.1: 1 BI 1467335, 1 mg:

Substance:	BI 1467335
Pharmaceutical formulation:	Tablet
Source:	Boehringer Ingelheim Pharma GmbH & Co KG<br>Birkendorfer Strasse 65<br>D-88397 Biberach a.d. Riss
Unit strength:	1 mg
Posology	QD
Route of administration:	Per os

Table 4.1.1: 2 BI 1467335, 5 mg:

Substance:	BI 1467335
Pharmaceutical formulation:	Tablet
Source:	Boehringer Ingelheim Pharma GmbH & Co KG<br>Birkendorfer Strasse 65<br>D-88397 Biberach a.d. Riss
Unit strength:	5 mg
Posology	QD
Route of administration:	Per os

Proprietary confidential information © 2018 Boehringer Ingelheim International GmbH or one or more of its affiliated companies

Table 4.1.1: 3 Placebo matching BI 1467335, 1 mg:

Substance:	BI 1467335
Pharmaceutical formulation:	Tablet
Source:	Boehringer Ingelheim Pharma GmbH & Co KG<br>Birkendorfer Strasse 65<br>D-88397 Biberach a.d. Riss
Unit strength:	n.a.
Posology	n.a.
Route of administration:	Per os

Table 4.1.1: 4 Placebo matching BI 1467335, 5 mg:

Substance:	BI 1467335
Pharmaceutical formulation:	Tablet
Source:	Boehringer Ingelheim Pharma GmbH & Co KG<br>Birkendorfer Strasse 65<br>D-88397 Biberach a.d. Riss
Unit strength:	n.a.
Posology	n.a.
Route of administration:	Per os

Proprietary confidential information © 2018 Boehringer Ingelheim International GmbH or one or more of its affiliated companies

4.1.3 Method of assigning patients to treatment groups

All treatments will be double-blind as to be indistinguishable for the patient as well as for the investigator. Patients are randomised to treatment groups at Visit 2. Note that the medication number is different from the patient number (the latter is assigned directly after informed consent was obtained). Site personnel will enter the medication number on the case report form/in the CRF.
During visit 2 eligible patients will be randomised to receive a 2:1:1:1:2 ratio according to a randomisation plan (see figure 3.1: 1 for details). The assignment will occur in a blinded fashion via Interactive Response Technology (IRT).

4.1.4 Drug assignment and administration of doses for each patient

IRT will allocate medication kit numbers at Visit 2, 4, and 6. The amount of trial medication dispensed and returned will be recorded on drug accountability forms.
For blinding reasons all treatments will consist of five tablets verum or placebo to be taken within 10-15 minutes in the morning depending on the treatment arm. This will not be changed during the entire study treatment period (V2-EOT).
For days without site visits, patients should be instructed to take the tablets orally with water in the morning before breakfast at approximately the same time every day without food (defined as no food intake until 60 mins after dosing). If a dose is missed by more than 8hrs, that dose should be skipped and the next dose should be taken as scheduled. No double doses should be taken and dose reductions are not permitted. Patients should be instructed to bring all unused drug and empty study blister to the study site.
Patients should be instructed NOT to take their study medication on the morning of scheduled trial visits. The first dose of study medication will be taken at the visit 2 under supervision of the investigator or site staff. At all site visits the morning dose of the investigational drug will be taken during the visit under supervision of the investigator or relevant site staff. The actual visit date and time of study drug administration at the trial visit will be recorded in the eCRF at each visit. Patients should be fasted at the beginning of the study visit. Patients who erroneously take the morning dose of study medication before coming to the clinic at a visit with scheduled PK samples should have the visit rescheduled as soon as possible, ideally on the following day. Patients will record the administration dates and times of all doses with the help of a diary as outlined in the Flow Chart and in Section 6.2.2.

Proprietary confidential information © 2018 Boehringer Ingelheim International GmbH or one or more of its affiliated companies

4.1.5 Blinding and procedures for unblinding

4.1.5.1 Blinding

Patients, investigators and everyone involved in trial conduct or analysis or with any other interest in this double-blinded trial will remain blinded with regard to the randomized treatment assignments until after database lock. Please refer to Section 4.1.5.2 for rules of breaking the code for patients in emergency situations.

The randomization code will be kept secret by Clinical Trial Support up to database lock. The randomization codes will be provided to bioanalytics prior to last patient out to allow for the exclusion from the analyses of PK samples taken from placebo patients and to a member of the unblinded team in preparation of a possible interim PK/PD analysis. Bioanalytics will not disclose the randomization code or the results of their measurements until the trial is officially unblinded.

A possible interim PK/PD analysis will be performed by an unblinded team independent from the trial and project teams to maintain the blinded conduct of the study. Results of the interim analysis will only be presented as aggregated data and not on subject level to maintain blinding. The logistical aspects of conducting the interim PK / PD analysis, and the plan to access of interim results will be described in the Interim Analysis Logistics Plan and Results Access Plan.

4.1.5.2 Unblinding and breaking the code

Emergency unblinding will be available to the Investigator / Pharmacist / investigational drug storage manager via IRT. It must only be used in an emergency situation when the identity of the trial drug must be known to the Investigator in order to provide appropriate medical treatment or otherwise assure safety of trial participants. The reason for unblinding must be documented in the source documents and/or appropriate CRF page along with the date and the initials of the person who broke the code.

Due to the requirements to report Suspected Unexpected Serious Adverse Reactions (SUSARs), it may be necessary for a representative from Boehringer Ingelheim’ s Pharmacovigilance group to access the randomisation code for individual patients during trial conduct. The access to the code will only be given to authorised Pharmacovigilance representatives and not be shared further.

4.1.6 Packaging, labelling, and re-supply

The investigational products will be provided by BI or a designated CRO. They will be packaged and labelled in accordance with the principles of Good Manufacturing Practice (GMP). Re-supply to the sites will be managed via an IRT system, which will also monitor expiry dates of supplies available at the sites.
For details of packaging and the description of the label, refer to the ISF.

Proprietary confidential information © 2018 Boehringer Ingelheim International GmbH or one or more of its affiliated companies

4.1.7 Storage conditions

Drug supplies will be kept in their original packaging and in a secure limited access storage area according to the recommended storage conditions on the medication label. IMP needs to be shipped and stored under refrigerated conditions (2-8°C / 36-46°F). A temperature log must be maintained for documentation.

If the storage conditions are found to be outside the specified range, the local clinical monitor (as provided in the list of contacts) must be contacted immediately.

4.1.8 Drug accountability

The Investigator, Pharmacist or investigational drug storage manager will receive the investigational drugs delivered by the sponsor when the following requirements are fulfilled:

- Approval of the trial protocol by the IRB / ethics committee
- Availability of a signed and dated clinical trial contract between the sponsor and the head of the investigational site,
- Approval/notification of the regulatory authority, e.g. competent authority,
- Availability of the curriculum vitae of the principal Investigator,
- Availability of a signed and dated clinical trial protocol
- Availability of the proof of a medical license for the principal Investigator
- Availability of Form 1572

The Investigator, Pharmacist or investigational drug storage manager must maintain records of the product’s delivery to the trial site, the inventory at the site, the use by each patient, and the return to the sponsor or warehouse / drug distribution centre or alternative disposal of unused products. If applicable, the sponsor or warehouse / drug distribution centre will maintain records of the disposal.

These records will include dates, quantities, batch / serial numbers, expiry (‘use- by’) dates, and the unique code numbers assigned to the investigational product and trial patients. The Investigator / Pharmacist / investigational drug storage manager will maintain records that document adequately that the patients were provided the doses specified by the CTP and reconcile all investigational products received from the sponsor. At the time of return to the sponsor< and/or >appointed CRO, the Investigator / Pharmacist / investigational drug storage manager must verify that all unused or partially used drug supplies have been returned by the clinical trial patient and that no remaining supplies are in the Investigator’s possession.

Proprietary confidential information © 2018 Boehringer Ingelheim International GmbH or one or more of its affiliated companies

4.2 OTHER TREATMENTS, EMERGENCY PROCEDURES, RESTRICTIONS

4.2.1 Other treatments and emergency procedures

There are no special emergency procedures to be followed.

4.2.2 Restrictions, warnings and precautions

Proprietary confidential information © 2018 Boehringer Ingelheim International GmbH or one or more of its affiliated companies

Proprietary confidential information © 2018 Boehringer Ingelheim International GmbH or one or more of its affiliated companies

4.3 TREATMENT COMPLIANCE

Patients are requested to bring all remaining trial medication including empty package material with them when attending visits.
Based on tablet counts, treatment compliance will be calculated as the number of tablets taken, divided by the number of tablets which should have been taken according to the scheduled period, multiplied by 100. Compliance will be verified by the on-site monitor authorised by the sponsor.

$$\text{Treatment compliance (\%)} = \frac{\text{Number of tablets actually taken} \times 100}{\text{Number of tablets which should have been taken}}$$

If the number of doses taken is not between 80-120%, site staff will explain the patient the importance of treatment compliance.

Proprietary confidential information © 2018 Boehringer Ingelheim International GmbH or one or more of its affiliated companies

5. VARIABLES AND THEIR ASSESSMENT

5.1 TRIAL ENDPOINTS

5.1.1 Primary Endpoint(s)

The primary endpoint is the plasma amine oxidase copper-containing 3 (AOC3) activity relative to baseline in %, 24 h post dose, after 12 weeks of treatment. The baseline is defined as the last AOC3 activity measurement prior to administration of any randomised study medication.

5.1.2 Secondary Endpoint(s)

Safety and tolerability will be assessed based on the number (%) of subjects with adverse reactions.

The secondary biomarker endpoints will be assessed based on the
relative ALT change from baseline after 12 weeks of treatment
relative AST change from baseline after 12 weeks of treatment
relative AP change from baseline after 12 weeks of treatment
relative γ-GT change from baseline after 12 weeks of treatment
relative caspase cleaved cytokeratin 18 (M30) change from baseline after 12 weeks of treatment
relative total cytokeratin 18 (M65) change from baseline after 12 weeks of treatment

5.2 ASSESSMENT OF EFFICACY

Not Applicable

Proprietary confidential information © 2018 Boehringer Ingelheim International GmbH or one or more of its affiliated companies

5.3 ASSESSMENT OF SAFETY

5.3.1 Physical examination

A full physical examination will be performed at the visits indicated in the Flow Chart. This should be performed according to medical standards and usually includes (but is not necessarily limited to) a review of the following organ systems: General appearance (including Skin), Head-Eyes-Ears-Nose-Throat (HEENT), Chest (including Pulmonary and Heart), Abdomen, Extremities, Urogenital and neurological assessment (basic mental status, cranial nerves, motor system, sensation, cerebellum/coordination). Clinically relevant abnormal findings noticed after randomisation will be reported as (S)AEs. At all other visits according to the Flow Chart a symptom-derived physical examination should be performed. This includes vital sign assessment as well as an evaluation of the organ systems associated with AE(s) symptoms or laboratory abnormalities.
Clinically significant abnormal findings will be reported as baseline conditions or AE's.

5.3.1.1 Waist and hip circumference

Waist and hip circumference will be measured at screening and EOT/ED visit.
Waist circumference measurements should be made around a patient's bare midriff, after the patient exhales while standing without shoes and with both feet touching and arms hanging freely. The measuring tape should be placed perpendicular to the long axis of the body and horizontal to the floor and applied with sufficient tension to conform to the measurement surface. Waist circumference should be determined by measuring the midpoint between the lowest rib and the iliac crest.
Hip circumference measurement should be taken around the widest portion of the buttocks.

5.3.1.2 Body weight

The scale used to capture body weight for each patient should remain consistent during the trial. In order to get comparable body weight values, it should be performed in the following way:
- Fasting (except for the screening visit);
- After the urine sampling (body weight after bladder voiding);
- Shoes and coat/jackets should be taken off; and
- Pockets should be emptied of heavy objects (i.e. keys, coins etc.)

5.3.2 Vital Signs

Systolic and diastolic blood pressure (BP), respiratory frequency (RF) and pulse rate (PR) will be measured after the patient has rested for at least 5 min in the sitting position.

Proprietary confidential information © 2018 Boehringer Ingelheim International GmbH or one or more of its affiliated companies

Measurement of vital signs should precede blood sampling to avoid the impact of blood sampling on the vital measurements. The measured vital signs will be documented in the source documents and recorded in the eCRF.

5.3.3 Safety laboratory parameters.

Parameters that will be determined during the trial conduct are listed in Table 5.3.3: 1 and will be collected by the trial site at the time points indicated in the Flow Chart. All analyses will be performed by a central laboratory. Patients have to be fasted (except for the screening visit) for at least 8 h before the blood sampling for the safety laboratory. The respective reference ranges and details about sample handling shipment and results reporting will be provided in the ISF (Lab Manual).
Clinically significant abnormal findings will be reported as baseline conditions or AE's.

Proprietary confidential information © 2018 Boehringer Ingelheim International GmbH or one or more of its affiliated companies

Table 5.3.3: 1 Safety laboratory parameters – whole blood, serum or plasma

Hematology	
Haematocrit	
Haemoglobin	WBC / Leukocytes
MCV, MCH, RDW, MCHC	Platelet Count / Thrombocytes
Red Blood Cells (RBC) / Erythrocytes	Differential Automatic (relative and absolute count):<br>Neutrophils, Eosinophils, Basophils, Monocytes, Lymphocytes
Coagulation	
Activated Partial Thromboplastin Time (aPTT)	
Prothrombin Time (INR)	
Fibrinogen	
Clinical chemistry	
Albumin	
Alkaline phosphatase	Creatine kinase (CK)
γ-GT (gamma-glutamyl transferase)<br>ALT (alanine aminotransaminase, SGPT)	CK-MB, troponin I (reflex tests if CK is elevated)
AST (aspartate aminotransaminase, SGOT)	eGFR*
	Fasting Plasma Glucose
Bicarbonate	Ferritin
Bilirubin Total	Plasma Insulin
Bilirubin Direct	Serum Free fatty acids
Bilirubin Indirect	Lactate dehydrogenase (LDH)
Calcium	Lipase
Chloride	Magnesium
Creatinine	Phosphate
hsC-reactive protein	Potassium
	Protein total
	Sodium
	Urea (BUN)
	LDL/HDL and total cholesterol
	Triglycerides
	TSH

* Estimated Glomerular filtration rate as assessed by the CKD-EPI formula (2009).

Proprietary confidential information © 2018 Boehringer Ingelheim International GmbH or one or more of its affiliated companies

Urine chemistry

Urine creatinine (for urine biomarker assessment, please see section 5.5.2)

Pregnancy Test

- Urine Pregnancy test[1]
- Serum Pregnancy test[2]

[1] Urine pregnancy test performed on-site at all dosing visits (pre-dose) as well as EOT and FU (only for female patients of childbearing potential).

[2] Serum pregnancy test at screening as well as confirmation of positive urine pregnancy test (only for female patients of child bearing potential at screening as well as reflex for positive urine pregnancy test).

5.3.4 Electrocardiogram

Printed paper traces from 12-lead ECGs in triplicate will be recorded at the visits outlined in the flowchart for all patients approximately 90 min after study drug intake. At visits without drug administration ECGs should preferentially be performed prior blood sampling. Please refer to the Flow Charts 1, 2, 3 and 4 for exact timing. ECGs will be recorded after the patients have rested for at least 5 minutes in a supine position. Six limb leads, as specified by Einthoven (I, II and III) and Goldberger (aVR, aVL, aVF), and six pre-cordial leads (V1–V6), according to Wilson, will be used. ECGs may be repeated for quality reasons and the repeated recording used for analysis.
In the event of any clinical cardiac symptoms (e.g. suspicion of heart rhythm disorders or cardiac ischaemia), an additional ECG will be recorded at the investigator´s discretion.
At screening visit all triplet ECGs will be printed and evaluated (signed, dated and commented upon) by the treating physician/ investigator and will be filed in the patient's source documents. This will be used as baseline before the first drug administration.
At all other visits, only the first of the three replicate ECGs at a single assessment time will be evaluated by the treating physician/ investigator and stored locally. The remaining second and third replicate ECGs will be printed and kept together with other medical records of the patient for additional analyses if required at a later time point.

All ECGs recorded during trial conduct including the baseline ECG will also be transmitted to a vendor for central evaluation and stored at an external central ECG database. Only the first of the three replicate ECGs at a single assessment time will be centrally evaluated and transferred to the sponsor. The results of the centralized evaluation will be sent from the ECG core lab to the sponsor according to a pre-specified data transmission agreement. The remaining second and third replicate ECGs will only be stored at the ECG vendor for additional analyses if required at a later time point. The central reader's evaluation of the tracing is considered the official reading for the trial.

Proprietary confidential information © 2018 Boehringer Ingelheim International GmbH or one or more of its affiliated companies

In case of discrepancies between investigator and central reading, the central evaluation will be valid.
Clinically significant abnormal findings will be reported either as baseline condition (if identified at the screening visit) or otherwise as adverse events and will be followed up and/or treated locally until normal or stable condition.

Information about the details of ECG collection will be provided in the ISF.

5.3.5 Assessment of adverse events

5.3.5.1 Definitions of AEs

Adverse event

An adverse event (AE) is defined as any untoward medical occurrence in a patient or clinical investigation subject administered a medicinal product and which does not necessarily have to have a causal relationship with this treatment.

An AE can therefore be any unfavourable and unintended sign (including an abnormal laboratory finding), symptom, or disease temporally associated with the use of a medicinal product, whether or not considered related to the medicinal product.

Serious adverse event

A serious adverse event (SAE) is defined as any AE which:

- results in death,
- is life-threatening, this refers to an event in which the patient was at risk of death at the time of the event; it does not refer to an event that hypothetically might have caused death if more severe.
- requires inpatient hospitalisation or
- prolongation of existing hospitalisation,
- results in persistent or significant disability or incapacity, or
- is a congenital anomaly / birth defect,
  or
- is to be deemed serious for any other reason if it is an important medical event when based upon appropriate medical judgment which may jeopardise the patient and may require medical or surgical intervention to prevent one of the other outcomes listed in the above definitions.

Medical and scientific judgement should be exercised in deciding whether other situations should be considered serious reactions, such as important medical events that might not be immediately life threatening or result in death or hospitalisation but might jeopardise the patient or might require intervention to prevent one of the other outcomes listed above. Examples of such events are intensive treatment in an emergency room or at home for allergic bronchospasm, blood dyscrasias or convulsions that do not result in hospitalisation or

Proprietary confidential information © 2018 Boehringer Ingelheim International GmbH or one or more of its affiliated companies

development of dependency or abuse. Any suspected transmission via a medicinal product of an infectious agent is also considered a serious adverse reaction.

AEs considered "Always Serious"

Cancers of new histology and exacerbations of existing cancer must be classified as a serious event regardless of the duration between discontinuation of the drug and must be reported as described in 5.3.5.2, subsections "AE Collection" and **AE reporting to sponsor and timelines"**

In accordance with the European Medicines Agency initiative on Important Medical Events, Boehringer Ingelheim has set up a list of further AEs, which by their nature, can always be considered to be "serious" even though they may not have met the criteria of an SAE as defined above.

The latest list of "Always Serious AEs" can be found in the EDC system. A copy of the latest list of "Always Serious AEs" will be provided upon request. These events should always be reported as SAEs as described above.

Adverse events of special interest (AESIs)

The term AESI relates to any specific AE that has been identified at the project level as being of particular concern for prospective safety monitoring and safety assessment within this trial, e.g. the potential for AEs based on knowledge from other compounds in the same class. AESI need to be reported to the sponsor's Pharmacovigilance Department within the same timeframe that applies to SAE, please see above.

The following are considered as AESIs:

Hepatic injury

A hepatic injury is defined by the following alterations of hepatic laboratory and clinical parameters as defined by the removal and stopping criteria in section 3.3.4.1.1 and section 10.3.

These lab findings constitute a hepatic injury alert and the patients showing these lab abnormalities need to be followed up according to the "DILI checklist" provided in the ISF. In case of clinical symptoms of hepatic injury (e.g. encephalopathy, nausea, vomiting, pruritus, severe fatigue, icterus, etc.) without lab results (ALT, AST, total bilirubin) available, the Investigator should make sure these parameters are analysed, if necessary in an unscheduled blood test. Should the results meet the criteria of hepatic injury alert, the procedures described in the DILI checklist should be followed.

Proprietary confidential information © 2018 Boehringer Ingelheim International GmbH or one or more of its affiliated companies

Intensity of AEs

The intensity (severity) of adverse events should be classified and recorded in the (e)CRF according to the Common Terminology Criteria for Adverse Events (CTCAE) Version 4.03 dated 14 June 2010 [R10-4848].

Causal relationship of AEs

The definition of an adverse reaction implies at least a reasonable possibility of a causal relationship between a suspected medicinal product and an adverse event. An adverse reaction, in contrast to an adverse event, is characterised by the fact that a causal relationship between a medicinal product and an occurrence is suspected.
Medical judgment should be used to determine the relationship, considering all relevant factors, including pattern of reaction, temporal relationship, de-challenge or re-challenge, confounding factors such as concomitant medication, concomitant diseases and relevant history.

Arguments that may suggest that there is a reasonable possibility of a causal relationship could be:

1. The event is consistent with the known pharmacology of the drug
2. The event is known to be caused by or attributed to the drug class.
3. A plausible time to onset of the event relative to the time of drug exposure.
4. Evidence that the event is reproducible when the drug is re-introduced
5. No medically sound alternative aetiologies that could explain the event (e.g. pre-existing or concomitant diseases, or co-medications).
6. The event is typically drug-related and infrequent in the general population not exposed to drugs (e.g. Stevens-Johnson syndrome).
7. An indication of dose-response (i.e. greater effect size if the dose is increased, smaller effect size if dose is diminished).

Arguments that may suggest that there is no reasonable possibility of a causal relationship could be:

1. No plausible time to onset of the event relative to the time of drug exposure is evident (e.g. pre-treatment cases, diagnosis of cancer or chronic disease within days / weeks of drug administration; an allergic reaction weeks after discontinuation of the drug concerned)
2. Continuation of the event despite the withdrawal of the medication, taking into account the pharmacological properties of the compound (e.g. after 5 half-lives).

    Of note, this criterion may not be applicable to events whose time course is prolonged despite removing the original trigger.
3. Additional arguments amongst those stated before, like alternative explanation (e.g. situations where other drugs or underlying diseases appear to provide a more likely explanation for the observed event than the drug concerned).
4. Disappearance of the event even though the study drug treatment continues or remains unchanged.

Proprietary confidential information © 2018 Boehringer Ingelheim International GmbH or one or more of its affiliated companies

5.3.5.2 Adverse event collection and reporting

AE Collection

The Investigator shall maintain and keep detailed records of all AEs in their patient files. The following must be collected and documented on the appropriate CRF(s) by the Investigator:

- From signing the informed consent onwards through the Residual Effect Period (REP), until individual patient's end of trial:
  - all AEs (serious and non-serious) and all AESIs.

  However, if an individual patient discontinues trial medication prematurely but stays in the trial (i.e. if further visits incl. telephone visits, or vital status assessments are planned) from then on and until the individual patient's end of the trial the Investigator must report related SAEs and related AESIs.
- After the individual patient's end of trial:
  the Investigator does not need to actively monitor the patient for AEs but should only report relevant SAEs and relevant AESIs of which the Investigator may become aware of.

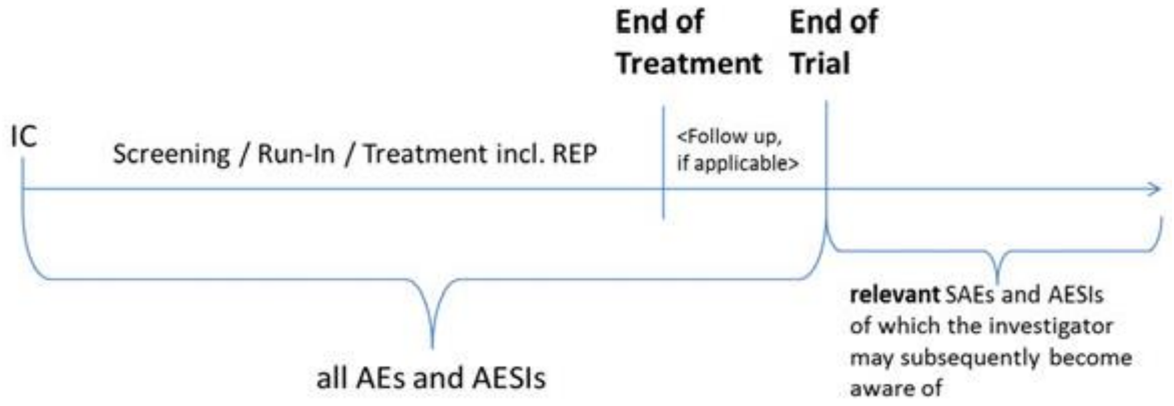

As the REP is to be explored in this trial, it is considered as the entire FU period from last dose administration of trial medication until individual patient's end of trial.
All AEs which occurred through the treatment phase until entire Follow-up period will be considered as on treatment, please see section 7.3.4.

AE reporting to sponsor and timelines

The Investigator must report SAEs, AESIs, and non-serious AEs which are relevant for the reported SAE or AESI, on the BI SAE form via fax immediately (within 24 hours) to the sponsor's unique entry point (country specific contact details will be provided in the ISF). The same timeline applies if follow-up information becomes available. In specific occasions the Investigator could inform the sponsor upfront via telephone. This does not replace the requirement to complete and fax the BI SAE form.

With receipt of any further information to these events, a follow-up SAE form has to be provided. For follow-up information the same rules and timeline apply as for initial information.

Proprietary confidential information © 2018 Boehringer Ingelheim International GmbH or one or more of its affiliated companies

Information required
For each AE, the Investigator should provide the information requested on the appropriate CRF pages and the BI SAE form. The Investigator should determine the causal relationship to the trial medication.
The following should also be recorded as an (S)AE in the CRF and SAE form (if applicable):

- Worsening of the underlying disease or of other pre-existing conditions
- Changes in vital signs, ECG, physical examination and laboratory test results, if they are judged clinically relevant by the Investigator.

If such abnormalities already pre-exist prior trial inclusion they will be considered as baseline conditions.

All (S)AEs, including those persisting after individual patient's end of trial must be followed up until they have resolved, have been sufficiently characterised, or no further information can be obtained.

Pregnancy
In rare cases pregnancy may occur in a clinical trial. Once a patient has been enrolled into this clinical trial and has taken trial medication, the Investigator must report immediately (within 24 hours) a potential drug exposure during pregnancy (DEDP) to the sponsor's unique entry point (country-specific contact details will be provided in the ISF). The Pregnancy Monitoring Form for Clinical Trials (Part A) should be used.

The outcome of the pregnancy associated with the drug exposure during pregnancy must be followed up and reported to the sponsor's unique entry point on the Pregnancy Monitoring Form for Clinical Trials (Part B).
The ISF will contain the Pregnancy Monitoring Form for Clinical Trials (Part A and B).

As pregnancy itself is not to be reported as an AE, in the absence of an accompanying SAE and/or AESI, only the Pregnancy Monitoring Form for Clinical Trials and not the SAE form is to be completed. If there is an SAE and/or AESI associated with the pregnancy an SAE form must be completed in addition.

Proprietary confidential information © 2018 Boehringer Ingelheim International GmbH or one or more of its affiliated companies

Proprietary confidential information © 2018 Boehringer Ingelheim International GmbH or one or more of its affiliated companies

Proprietary confidential information © 2018 Boehringer Ingelheim International GmbH or one or more of its affiliated companies

5.5 ASSESSMENT OF BIOMARKER(S)

Proprietary confidential information © 2018 Boehringer Ingelheim International GmbH or one or more of its affiliated companies

5.5.2 Biochemical and cellular biomarkers

Concentration and activity of AOC3 in plasma will be measured at the time points indicated in the Flow Charts 1, 2, 3, 4 and in Section 10.1. The values determined prior to the first dosing of BI 1467335 will be defined as baseline.
AOC3 activity per time point will be presented relative to baseline. AOC3 concentration will be presented as absolute values and relative to baseline.

The following parameters will be determined for plasma AOC3 activity relative to baseline, if feasible; after multiple dosing the parameters will be denoted with the index 'N', where N is the number of doses administered.

E_{max} (maximum plasma AOC3 activity relative to baseline within the dosing interval)

E_{min} (minimum plasma AOC3 activity relative to baseline within the dosing interval)

t_{max} (time from dosing to reach E_{max})

t_{min} (time from dosing to reach E_{min})

$E_{pre,N}$ (plasma AOC3 activity relative to baseline prior to the N_{th} dose of BI 1467335)

.

For the secondary endpoints ALT, AST, ALP, γ-GT, caspase cleaved cytokeratin 18 (M30) and total cytokeratin 18 (M65) samples will be collected as described in the Flow Chart 1.

Relative changes from baseline in ALT, AST, AP,γ-GT and Cytokeratine18 fragments after 2, 4, 8, 12 weeks of treatment and 4 weeks after last dose will be determined.

For the endpoints and exploratory biomarkers clear endpoint definitions and further details on evaluation will be provided in the TSAP.

Proprietary confidential information © 2018 Boehringer Ingelheim International GmbH or one or more of its affiliated companies

5.5.3 Methods of sample collection

For the measurement of the activity and the concentrations of AOC3 4.0 ml of blood will be taken from an antecubital or forearm vein into a K2-EDTA anticoagulant blood drawing tube at the time points indicated in the Flow Chart. 2 x 4.0 ml will be taken at Visit 2 to have a back-up sample for the pre-dose value.
Blood will be withdrawn by means of either an indwelling venous catheter or by venepuncture with a metal needle.

For the secondary endpoints and the exploratory biomarkers evaluation approximately 23 ml of blood (10 ml for K2-EDTA blood and 10 ml for serum and 3ml for sodium citrate coagulation tube) will be taken at the time points indicated in the Flow Chart 1. Blood will be withdrawn by means of either an indwelling venous catheter or by venepuncture with a metal needle.
Further details on collection, handling, storage and processing of the blood sample will be provided in a separate laboratory instruction manual.

All left over samples will be stored and used for not yet specified explorative investigations. These samples will be stored for up to three years after the end of the clinical trial and may be used for not yet specified biomarker analyses to enable further characterization of metabolic diseases and their progress, as well as method development and evaluation. Results of these assessments will not be part of the Clinical Trial Report.

Proprietary confidential information © 2018 Boehringer Ingelheim International GmbH or one or more of its affiliated companies

Proprietary confidential information © 2018 Boehringer Ingelheim International GmbH or one or more of its affiliated companies

5.5.5 Appropriateness of Measurements

All measurements performed during this trial are standard measurements and will be performed in order to monitor subjects' safety and to determine pharmacokinetic and pharmacodynamic parameters in an appropriate way. The scheduled measurements will allow monitoring of changes in vital signs, standard laboratory values, and ECG parameters that might occur as a result of administration of trial medication. The safety assessments are standard, are accepted for evaluation of safety and tolerability of an orally administered drug, and are widely used in clinical trials. The pharmacokinetic parameters and measurements outlined in Section 5.4 are generally used assessments of drug exposure.

Proprietary confidential information © 2018 Boehringer Ingelheim International GmbH or one or more of its affiliated companies

6. INVESTIGATIONAL PLAN

6.1 VISIT SCHEDULE

All patient visits should be scheduled according to the Flow Chart. Each visit date (with its window) is to be counted from Day 1. If any visit has to be rescheduled, subsequent visits should follow the original visit schedule. The trial medication packs contain sufficient medication to allow for these time windows.
All trial visits should be initiated preferentially in the morning starting before 9:00 AM. Patients should be instructed to avoid intake of the morning dose of the study medication at home at scheduled visit days as they will be dosed whilst at the study site.
Study measurements and assessments according to the Flow Chart scheduled to occur before study drug administration on Day 1, 15, 29, 43, 57 and 85 are to be performed and completed within a 2 h-period prior to the study drug administration.
Unscheduled visits will be possible at the discretion of the investigator at any time in order to check the safety of the patient.
If the reason for removal of a patient from the treatment is an adverse event or an abnormal laboratory test result, the patient must be followed until complete resolution or stabilization of the event or until follow-up is agreed adequate by the Investigator and BI Clinical Monitor.

6.2 DETAILS OF TRIAL PROCEDURES AT SELECTED VISITS

6.2.1 Screening

Screening Period
All patients must sign an Informed Consent consistent with ICH-GCP guidelines and the local legislation prior to any study specific procedures. Once they have consented, the patient is considered to be enrolled in the trial and have started screening. The patient should be recorded on the enrolment log and be registered in IRT as a screened patient. Patients will be assigned a patient number and enrolment must be recorded in eCRF pages. The Screening period is defined as the period from the Screening visit to Randomisation (first study drug administration). The screening period should be no longer than 28 days and no less than 7 days and will be used to assess eligibility of the patients. Thus patients will not be randomized until all screening procedures are completed and results are reviewed to verify study eligibility. Please refer to Section 8.1.
For the comprehensive list of the trial procedures required at the Screening Visit (Visit 1) please refer to the Flow Chart.
Patients are allowed to repeat lab testing once during the screening period.
For those patients with historic ALT > 1.25 ULN value more than 3 months prior to screening, the ALT > 1.5x ULN must be confirmed via two measurements at least 1 week apart within the screening period.

Proprietary confidential information © 2018 Boehringer Ingelheim International GmbH or one or more of its affiliated companies

Patients without required clinical imaging results suggestive of NASH (no more than 3 years prior to screening, according to inclusion criterion #1), clinical imaging can be performed as optional procedure during the screening period. The imaging procedures will be performed according to local standards. Please refer to Flow Chart 1.
No imaging is required if histological evidence of NASH (no more than 3 years prior to screening) is available. Please refer to section 3.3.2

Demographics
Information on race will be collected because this is required for the calculation of eGFR (CKD-EPI formula).

Re-screening
Patients who screen-fail the study after Visit 1 should be registered in the IRT as a screen failure within the protocol defined screening period.
Re-screening of not yet randomised patients can be allowed in exceptional cases but should be discussed on a case-by-case basis between the study site, Monitor staff and with the TCM.

IRT
All patients that are screened must be registered with IRT. If the patient results in a screen failure, IRT should be notified as soon as possible and within the 28- day screening period. Details of IRT procedures can be found in the IRT manual located in the ISF

6.2.2 Treatment period

Visits 2, 3, 4, 5, 6 and EOT
The treatment period consists of a maximum of 6 visits (visits 2 - EOT). Visit 2 is the randomisation visit and visit EOT is the end of treatment visit, where the last dose of medication will be administered. Procedures are to be performed according to the flowcharts 1, 2, 3, 4, section 10.1 and the respective protocol sections.

Safety laboratory testing
Patients should be fasted (no food and only water) for at least 8 hours prior to the start of procedures until 60 mins after drug intake. If a patient comes in non-fasted where a fasting condition is required, the visit should be performed, the non-fasted condition documented on the laboratory requisition, and the patient reminded about the expected condition.

Pregnancy testing
Urine pregnancy testing for all women of childbearing potential will be conducted on-site prior to every dosing and must be negative to further treat the patient. A positive urine test must be confirmed with a serum pregnancy test.

Randomisation (Visit 2)
Randomisation via IRT and administration of the first dose of study medication will occur at Visit 2.

Proprietary confidential information © 2018 Boehringer Ingelheim International GmbH or one or more of its affiliated companies

Study drug diary

The study patients will be instructed to complete a study drug diary during the treatment phase to document drug intake (date and time) and number of the tablet row indicating the day of intake between the on-site visits.
The diary will be dispensed to the patients at Visit 2 and the patient´s entries will be checked by site-staff during the consecutive treatment visits. Selected entries (drug administration date and time 3 days prior to each visit) will be entered by site-staff into the CRF.

Pharmacokinetic and biomarker blood sampling

The blood sampling times given in Section 10.1 are the recommended sampling times relative to the drug administration and should be adhered to as closely as possible. The actual times of drug administration and blood samplings will be recorded in the CRF.
Please refer to the Flow Charts 1, 2, 3 and 4 for exact timing.

Telephone contacts

Day 27/ Phone Call 3A:
The site staff should call the patients on day 27 to remind them of the urine collection starting directly after the drug administration at day 28 (24h prior to planned visit 4/ day 29 drug administration) and study drug intake.
Day 83/ Phone Call 6A:
The site staff should call the patients on day 83 to remind them of study drug intake on day 84. Please refer to section PK urine collection above and Flow Charts 1, 4 and section 10.1.

End of Treatment (EOT) Visit/ Early Discontinuation (ED):

If the regular end of the treatment period at week 12 is reached, the EOT Visit will be completed. The time interval for the regularly scheduled EOT Visit is 4 days, i.e. Day 85+3. The overall duration of the treatment period (randomisation to EOT) should not be less than 85 days.
If the patient refuses to continue the treatment in the trial or must stop treatment the early discontinuation (ED) Visit will be completed instead of the planned treatment period visit. This early discontinuation visit will include the same procedures as the normal EOT visit except drug administration, PK, PG and PD sampling. Visit FU should be performed no later than four weeks after the last study drug intake.

Proprietary confidential information © 2018 Boehringer Ingelheim International GmbH or one or more of its affiliated companies

It is important to distinguish between premature study drug discontinuation and complete withdrawal of consent to participate in further study procedures. Patients not willing to continue study drug intake should be asked to further attend scheduled trial visits, follow-up visits and assessments until the end of the trial unless they withdraw consent to participate in the study. For patients who discontinue IMP but are willing to attend scheduled trial visits, the early discontinuation (ED) Visit will be completed according to FlowChart instead of the next planned treatment period visit. All following visits should be performed according to the initial study schedule (without study drug administration). In addition to the safety sampling, only one PK sample and one PD blood sample for measurement of AOC3 activity should be taken at each visit on which PK and/or PD sampling was originally planned and the actual time of the blood draw recorded. One of these remaining visits should be performed no later than four weeks after the last study drug intake.
Should it not be possible to attend all visits, at least phone contacts should occur at the scheduled visits time points. It is vital to explain to these patients the importance to continue trial participation.

All unused study medication will be collected and the study drug diary will be checked by the site. Procedures are to be performed according to the Flow Chart.

6.2.3 Follow Up Period and Trial Completion

Follow-up (FU):
This visit will be performed 28±3 days after EOT (see Flow Chart 1 and Section 10.1 for procedures to be performed).
In case of early discontinuation this visit will include the same procedures as the normal FU visit except PG and PD sampling.
All abnormal values (including laboratory parameters) that are judged clinically relevant by the investigator will be monitored using the appropriate tests until a return to a medically acceptable level is achieved. Adverse events persisting after trial completion must be monitored until they have normalised or have been sufficiently characterised.

Trial completion:
Trial completion is defined as patients completing the FU visit within the specified window per the flow chart and who have not discontinued drug prematurely.

Proprietary confidential information © 2018 Boehringer Ingelheim International GmbH or one or more of its affiliated companies

7. STATISTICAL METHODS AND DETERMINATION OF SAMPLE SIZE

7.1 STATISTICAL DESIGN - MODEL

See Section 3.1 for details on the design of the study.
See Section 2.2 for details on the objective of the study.

Primary endpoint is the plasma AOC3 activity relative to baseline in %, 24 hours post dose, after 12 weeks of treatment. Baseline is defined as the last measured plasma AOC3 activity prior to administration of any randomised study medication.

The dose-response relationship of the primary endpoint will be analysed using a nonlinear regression model applied to the AOC3 activity at week 12. The fitted regression model will be used to derive the smallest dose where the mean plasma AOC3 activity curve drops below 10% (D10). This corresponds to:

$$\mathrm{D10} = \min\{d \in [0,10] \,|\, \widehat{f(d)} \leq 10\%\}$$

where d is the dose and $\hat{f}()$ is the fitted dose-response curve described in Section 7.3.1.

The analyses of the secondary biomarker endpoints ALT AST, AP, γ-GT and CK18 fragments will follow the MCPmod approach allowing for the simultaneous evaluation of different potential dose response patterns, whilst protecting the overall probability of type I error (one-sided alpha of 0.05).

7.2 NULL AND ALTERNATIVE HYPOTHESES

In this trial it is not planned to test any statistical hypotheses in a confirmatory sense. Where statistical testing is used it will have to be interpreted in the perspective of the exploratory character of the study.

No hypothesis testing will be used for the primary analysis of the primary endpoint.
The analyses of the secondary biomarker endpoints ALT, AST, AP, γ-GT and CK18 fragments contain hypotheses testing. The null hypothesis is that there is a flat dose response pattern across placebo and any dose of BI 1467335 within the tested dose range (0-10mg). The alternative hypothesis is that there is a non-flat dose response pattern indicating a benefit of BI 1467335 compared to placebo.

Proprietary confidential information © 2018 Boehringer Ingelheim International GmbH or one or more of its affiliated companies

7.3 PLANNED ANALYSES

The following patient analysis sets are defined for this trial:

- Treated Set (TS): includes all patients who signed informed consent and were treated with at least one dose of the trial medication. Patients in TS are analyzed under the actual trial medication received at randomisation. The TS is used for safety analyses as well as demographics and baseline characteristics.

- Full Analysis Set (FAS): includes all patients in treated set who had non-missing baseline and at least one non-missing post-baseline and on-treatment measurement on any biomarker endpoint. Patients in FAS are analyzed according to the intent-to-treat principle, i.e., patients in FAS are analyzed under the randomized trial medication.

- Per protocol set (PPS): includes all patients from the FAS without important protocol violations (IPV) leading to exclusion. This includes:

Compliance to randomised treatment within 80% -120% (inclusive)
At least 80% treatment compliance within 4 weeks before last visit

Data from subjects who are screened but not randomised will be listed but not included in any summary statistics or inferential statistics. Specifications of important protocol violations leading to exclusion will be provided in the TSAP.

Further Analysis sets will be defined in the TSAP.

7.3.1 Primary endpoint analyses

The primary analysis of the primary endpoint will be based on the PPS.

The subject specific plasma AOC3 activity at time t relative to baseline in % is calculated via

$$\%AOC3a_t := [(AOC3a_t - AOC3a_{t,back})/(AOC3a_{base} - AOC3a_{base,back})] * 100$$

Where $AOC3a_t$ is the AOC3 activity measured at time t, $AOC3a_{t,back}$ is the background noise at time t, $AOC3a_{base}$ is the AOC3 activity measured at baseline and $AOC3a_{base,back}$ is the background noise at baseline.

As the primary endpoint should show a reduction in AOC3 activity with higher doses, a decreasing 3-Parameter regression curve will be used of the form

$$\%AOC3a_{12,i} = \frac{TOP * D_i^N}{ED50^N + D_i^N} + s_i$$

Proprietary confidential information © 2018 Boehringer Ingelheim International GmbH or one or more of its affiliated companies

where

$\%AOC3a_{12,i}$	is the response value of patient i, 24h after drug administration at week 12 (EOT visit),
TOP	is the highest AOC3 activity,
D_i	is the administered drug amount to patient i
$ED50$	is the dose, which produces half of TOP
N	is the slope factor determining the steepness of the dose-response curve,
s_i	is the random error term of patient i.

Note that the dose-response relationship is assumed to asymptotically approach 0% at high doses.

As it is expected to see a higher variability in AOC3 activity measurements in placebo treated patients then in patients treated with BI 1467335 the model fit will use power of mean variance estimates (POM) to account for heterogeneity.

In case of the 24h value after drug administration at week 12 is missed, the -0:30 values at week 12 can be used instead. In case of other missing AOC3 activity values, except of missing baseline where no imputation is done, multiple imputation will be used. After exploring the missing data mechanism and observed measurements on the blinded data the variable AOC3 activity relative to baseline in % will be included in the imputation model.
If the data is monotone missing, a regression model will be used for imputation including baseline AOC3 activity, and AOC3 activity relative to baseline measurements predose (-0:30h relative to drug administration) at Visit 4 and 6 and 24h relative to drug administration at visit 3,5 and EOT by treatment.
In the case of non-monotone missing, a Markov chain Monte Carlo (MCMC) step will be used to create monotone missing data in multiple datasets. The regression method will then be used to complete the imputation in each dataset. For each imputed complete dataset, the decreasing 3-parameter curve model will be used for the analysis. The results will be pooled following the standard multiple imputation procedure.

The dose where the model fit drops below 10% and the 95% confidence interval will be determined (D10). The fitted curve will be graphically displayed.

Proprietary confidential information © 2018 Boehringer Ingelheim International GmbH or one or more of its affiliated companies

7.3.2 Secondary endpoint analyses

The number (%) subjects with adverse reactions will be evaluated descriptively on the TS. For more details see Section 7.3.4.

The primary analysis of the secondary biomarker endpoints will be first analysed using a repeated measurement analysis (MMRM) on the PPS. The relative ALT (AST, AP, γ-GT and CK18 fragments respectively) changes from baseline at visit 3,4,5,6 and EOT will be evaluated on log scale using an MMRM accounting for the following sources of variation: ‘baseline’, ‘visit’, ‘treatment’, ‘visit*treatment’ and ‘visit*baseline’ interaction as fixed effect as well as the random ‘subject’ effect. The unstructured covariance structure will be used to model the within patient measurements. To estimate denominator degrees of freedom the Kenward-Roger approximation will be used.
The dose-response relationship of the week 12 estimates from MMRM will then be analysed using MCPMod. A set of plausible dose response patterns is considered with pre-specified parameters define:

The following model assumptions have been selected to cover a plausible and diverse range of dose response patterns when having matching Placebo and active BI 1467335 doses of 1 mg, 3 mg, 6 mg and 10 mg:

- Linear: no assumptions needed
- Linear logistic no assumptions needed
- Quadratic: 90 % of the maximum effect is achieved at 7 mg
- Exponential: 90 % of the maximum effect is achieved at 7 mg
- Emax: 90 % of the maximum effect is achieved at 7 mg
- Sigmoidal Emax: 30 % of the maximum effect is achieved at 3 mg and 90 % of the maximum effect is achieved at 7 mg
- Logistic: 30 % of the maximum effect is achieved at 3 mg and 90 % of the maximum effect is achieved at 7 mg
- Betamod: 90 % of the maximum effect is achieved at 5 mg, maximum effect is achieved at 4 mg and maximal dose is 10 mg

Proprietary confidential information © 2018 Boehringer Ingelheim International GmbH or one or more of its affiliated companies

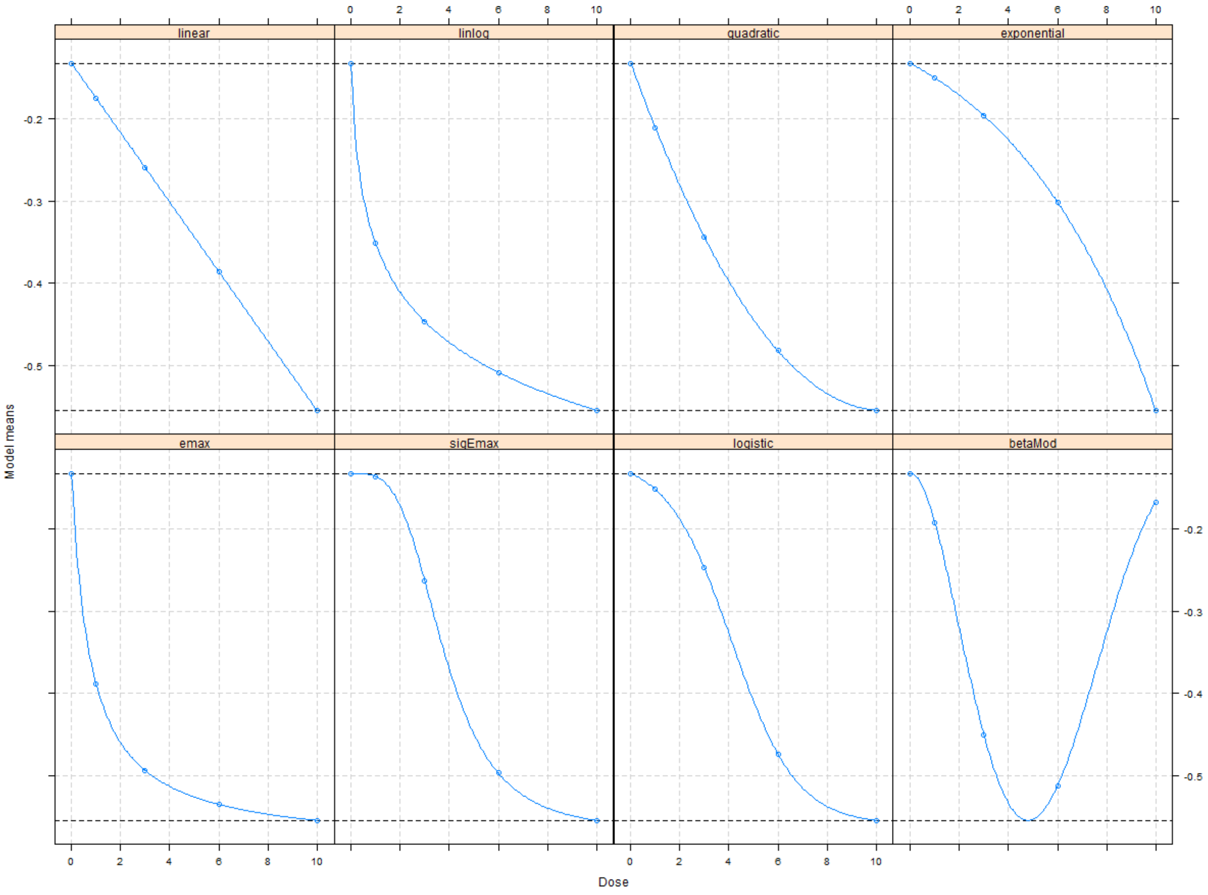

Figure 7.3.2: 1 Defined MCPMod shapes to be used.

If there is a at least one significant model, the null hypothesis of a flat dose-response relationship for the secondary endpoint ALT (for AST, AP, γ-GT and CK18 fragments respectively) jointly for each of the candidate dose-response models with a contrast test controlled for the family-wise error rate ($\alpha = 0.05$ 1-sided) will be rejected.
When the null hypothesis is rechecked, the best-fitting model from the above set of eight models can be refitted to the data without any parameter assumptions to generate new estimates of the model parameters from the data. The target dose will be obtained via model averaging across the significant models based on Akaike Information Criterion (AIC) (the smaller the AIC value the better the model fit). The target dose(s) can then be determined from that model by incorporating information on the minimum clinically relevant effect as well as safety information.

Further details on the primary analysis, will be provided in the TSAP.

Proprietary confidential information © 2018 Boehringer Ingelheim International GmbH or one or more of its affiliated companies

7.3.4 Safety analyses

Adverse events will be coded using the Medical Dictionary for Regulatory Activities (MedDRA) coding dictionary. Standard BI summary tables and listings will be produced. All adverse events with an onset between start of treatment and individual patient's end of trial will be assigned to the treatment period for evaluation.
The analysis will be done by 'treatment at onset'.
All treated patients will be included in the safety analysis (analysis on TS). In general, safety analyses will be descriptive in nature and will be based on BI standards. No hypothesis testing is planned.

Statistical analysis and reporting of adverse events will concentrate on treatment-emergent adverse events. To this end, all adverse events occurring between start of treatment and individual patient's end of trial will be considered 'treatment-emergent'. Adverse events that start before first drug intake and deteriorate under treatment will also be considered as 'treatment-emergent'.

Frequency, severity, and causal relationship of adverse events will be tabulated by system organ class and preferred term after coding according to the current version of the Medical Dictionary for Drug Regulatory Activities (MedDRA). Frequency tables for all adverse events, protocol-specified AESI, serious adverse event (SAE), adverse event leading to death, adverse event leading to discontinuation, investigator assessed drug-related adverse event and serious adverse event will be generated for treatment-emergent adverse events.

Laboratory data will be analysed both quantitatively as well as qualitatively. The latter will be done via comparison of laboratory data to their reference ranges. Values outside the reference range as well as values defined as clinically relevant will be highlighted in the listings. Treatment groups will be compared descriptively with regard to distribution parameters as well as with regard to frequency and percentage of patients with abnormal values or clinically relevant abnormal values.

Vital signs, physical examinations, or other safety-relevant data observed at screening, baseline, during the course of the trial and at the end-of-trial evaluation will be assessed with regard to possible changes compared to findings before start of treatment.

Proprietary confidential information © 2018 Boehringer Ingelheim International GmbH or one or more of its affiliated companies

7.4 INTERIM ANALYSES

An interim PK/PD analysis may be performed for model development purposes of the population PK and PK/PD analysis and to support dose decision for future trials. The interim analysis will focus on PK and AOC3 inhibition; other secondary biomarker endpoints may be evaluated in addition. The exact time point for data cut-off will be aligned with other studies investigating BI 1467335 and general project planning. Details of this analysis will be defined in the interim PK/PD analysis plan.

The result of the interim analysis will not be used to make any adaptions of the trial like sample size adjustments.

For steps to maintain blinding during the interim analysis see Section 4.1.5.1.

7.5 HANDLING OF MISSING DATA

Primary and secondary biomarker endpoints

Every effort will be made to collect complete data at the specified time points.

If missing values still occur they will be handled as described in the corresponding section.

Safety

With respect to safety evaluations, it is not planned to impute missing values.

7.6 RANDOMISATION

Patients will be randomised in blocks to double-blind treatment in a 2:1:1:1:2 ratio (Placebo, 1 mg, 3 mg, 6mg and 10 mg respectively). The randomisation of patients to the treatment groups will be performed via an interactive response technology (IRT).
BI will arrange for the randomisation and the packaging and labelling of trial medication. The randomisation list will be generated using a validated system, which involves a pseudo-random number generator so that the resulting treatment will be both reproducible and non-predictable. The block size will be documented in the CTR. Access to the codes will be controlled and documented.

Proprietary confidential information © 2018 Boehringer Ingelheim International GmbH or one or more of its affiliated companies

7.7 DETERMINATION OF SAMPLE SIZE

The sample size calculation is based on both, plasma AOC3 activity relative to baseline and ALT change from baseline, the latter being the biomarker with the least favorable ratio of anticipated effect size to variance.

Under the assumptions given in detail below, a sample size of 98 with an allocation ratio of 2:1:1:1:2 for Placebo, 1 mg, 3 mg, 6mg and 10 mg respectively is appropriate for both the estimation of D10 of plasma AOC3 activity relative to baseline and the MCPMod analysis of biomarkers. This assumes 98 evaluable patients for the primary and secondary endpoints and thus around 108 will need to be randomized. For the dose assessment the lowest (Placebo) and highest (10 mg BI 1467335) dose groups will have the highest impact on the analysis. Due to the nature of the dose- response-relationship, the biggest difference is anticipated between Placebo and the 10 mg group. The power analysis and simulation via MCPMod supported the 2:1:1:1:2 allocation ratio instead of equal allocations (see also Table 7.7:1).

ALT

Sample size is based on four assumptions:

- an assumed maximum effect size of BI 1467335 vs. placebo of 30% for relative change from baseline in ALT after 12 weeks of treatment
- a target dose of 20% difference to placebo can be estimated
- the target dose lies within the tested dose range
- MCPMod shapes and guesstimates as described below

The defined response patterns and model assumptions from section 7.3.2 are used.

Further assumptions are a mean baseline ALT of 80 U/L, a placebo effect at week 12 of -10 U/L and an effect of active treatment at week 12 of -34 U/L, i.e. the true difference between the relative change from baseline between placebo and active treatment at week 12 is assumed to be 30%. Further on, it is assumed to have a constant coefficient of variation over the whole ALT range of 40/80 = 0.5. This assumption is based on external data from CENTAUER, GOLDEN and ARAMCHOL (R16-5489; R18-2779; R18-2781; mean baseline ALT of 63-78 and respective SD of 31-44) and on blinded estimated SD for baseline ALT from 61 patients from this study.

On log scale this corresponds to a change from baseline in the placebo group of -0.14 and in the active treatment group of -0.56, i.e. a difference of 0.42.

An autoregressive correlation structure is assumed with constant variability of ALT on log scale regardless of visit and exponentially declining correlation with distance. The correlation between measurements at visits four weeks apart are assumed to be 0.7. Consequently, correlation between baseline and week 12 is 0.34

Proprietary confidential information © 2018 Boehringer Ingelheim International GmbH or one or more of its affiliated companies

MCMod will be applied to change from baseline of ALT at week 12 on log scale. It is investigated if (i) the MCPMod testing step rejects the null hypothesis of no dose-response relationship at one-sided alpha = 0.05, and (ii) a target dose of 20% difference to placebo, corresponding to a Delta on log-scale of 0.26, can be estimated and lies within the tested dose range.

Simulations show that under the assumptions given above and assuming a true effect of 30% (Situation 3), the probability to fulfil the two conditions is approx. 84% with a sample size of 98 using an allocation ratio of 2:1:1:1:2 for doses Placebo, 1 mg, 3 mg, 6mg and 10 mg respectively..

Table 7.7:1 Possible scenarios to fulfil the two conditions using the assumptions define above.

SD	N per arm (total)	True assumed effect	probability to fulfil the two conditions
40	28 : 14 : 14 : 14 : 28 (98)	Situation 1 with true assumed effect of 0.001%	5.3%
		Situation 2 with true assumed effect of 20%	50.4%
		Situation 3 with true assumed effect of 30%	84.8%

Using these parameter estimates, datasets with 98 patients in a 2:1:1:1:2 ratio were simulated (1000 repeats). The D10 of the fitted nonlinear curves using simulated data was between 3.95 mg and 5.1 mg, the 5% and 95% quantiles of the distribution being 4.2 mg and 4.8 mg, respectively. This precision is considered to be sufficient for the purpose of the trial.

Proprietary confidential information © 2018 Boehringer Ingelheim International GmbH or one or more of its affiliated companies

Figure 7.7:1: Boxplot of estimated D10 using 1000 simulated datasets.

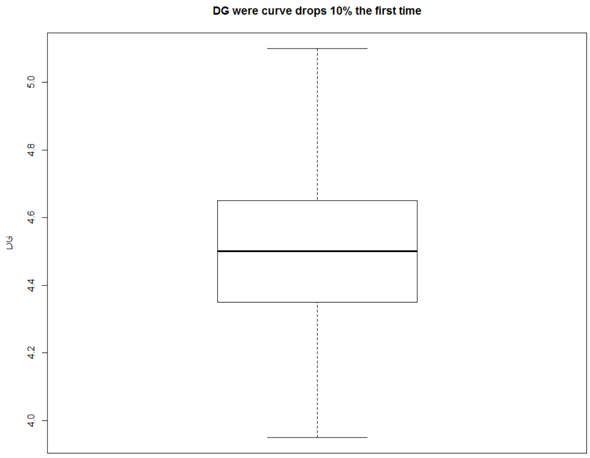

Simulation shows that a 10% drop out rate does not decrease the precision of D10 estimation to an unacceptable degree.

The calculations have been performed using R version 3.3.2 as well as SAS Version 9.4. The R and SAS codes for the sample size calculations will be documented in a separate file and stored in the TMF.

Proprietary confidential information © 2018 Boehringer Ingelheim International GmbH or one or more of its affiliated companies

8. INFORMED CONSENT, TRIAL RECORDS, DATA PROTECTION, PUBLICATION POLICY

The trial will be carried out in compliance with the protocol, the ethical principles laid down in the Declaration of Helsinki, in accordance with the ICH Harmonized Tripartite Guideline for Good Clinical Practice (GCP), relevant BI Standard Operating Procedures (SOPs), the EU regulation 536/2014 and other relevant regulations.

Standard medical care (prophylactic, diagnostic and therapeutic procedures) remains in the responsibility of the treating physician of the patient.

The Investigator will inform the sponsor immediately of any urgent safety measures taken to protect the trial subjects against any immediate hazard, and also of any serious breaches of the protocol or of ICH GCP*.

The Boehringer Ingelheim transparency and publication policy can be found on the following web page: trials.boehringer-ingelheim.com. The rights of the Investigator and of the sponsor with regard to publication of the results of this trial are described in the Investigator contract. As a rule, no trial results should be published prior to finalization of the Clinical Trial Report.

8.1 TRIAL APPROVAL, PATIENT INFORMATION, INFORMED CONSENT

This trial will be initiated only after all required legal documentation has been reviewed and approved by the respective Institutional Review Board (IRB) / Independent Ethics Committee (IEC) and competent authority (CA) according to national and international regulations. The same applies for the implementation of changes introduced by amendments.
Prior to patient participation in the trial, written informed consent must be obtained from each patient (or the patient's legally accepted representative) according to ICH / GCP and to the regulatory and legal requirements of the participating country. Each signature must be personally dated by each signatory and the informed consent and any additional patient-information form retained by the Investigator as part of the trial records. A signed copy of the informed consent and any additional patient information must be given to each patient or the patient's legally accepted representative.
The Investigator must give a full explanation to trial patients based on the patient information form. A language understandable to the patient should be chosen, technical terms and expressions avoided, if possible. The patient must be given sufficient time to consider participation in the trial. The Investigator obtains written consent of the patient's own free will with the informed consent form after confirming that the patient understands the contents. The Investigator must sign (or place a seal on) and date the informed consent form. If a trial collaborator has given a supplementary explanation, the trial collaborator also signs (or places a seal on) and dates the informed consent.
Re-consenting may become necessary when new relevant information becomes available and should be conducted according to the sponsor's instructions.

Proprietary confidential information © 2018 Boehringer Ingelheim International GmbH or one or more of its affiliated companies

The consent and re-consenting process should be properly documented in the source documentation.

8.2 DATA QUALITY ASSURANCE

A quality assurance audit/inspection of this trial may be conducted by the sponsor, sponsor's designees, or by IRB / IEC or by regulatory authorities. The quality assurance auditor will have access to all medical records, the Investigator's trial-related files and correspondence, and the informed consent documentation of this clinical trial.

8.3 RECORDS

Case Report Forms (CRF) for individual patients will be provided by the sponsor. See Section 4.1.5.2 for rules about emergency code breaks. For drug accountability, refer to Section 4.1.8.

8.3.1 Source documents

In accordance with regulatory requirements the Investigator should prepare and maintain adequate and accurate source documents and trial records that include all observations and other data pertinent to the investigation on each trial subject. Source data as well as reported data should follow good documentation practices and be attributable, legible, contemporaneous, original and accurate. Changes to the data should be traceable (audit trail). Data reported on the CRF must be consistent with the source data or the discrepancies must be explained.
The current medical history of the subject may not be sufficient to confirm eligibility for the trial and the Investigator may need to request previous medical histories and evidence of any diagnostic tests. In this case the Investigator must make three documented attempts to retrieve previous medical records. If this fails a verbal history from the patient, documented in their medical records, would be acceptable.
Before providing any copy of patients' source documents to the sponsor the investigator must ensure that all patient identifiers (e.g. patient's name, initials, address, phone number, social security number) have properly been removed or redacted to ensure patient confidentiality.

If the patient is not compliant with the protocol, any corrective action e.g. re-training must be documented in the patient file.
For the CRF, data must be derived from source documents, for example:

- Patient identification: gender, year of birth (in accordance with local laws and regulations)
- Patient participation in the trial (substance, trial number, patient number, date patient was informed)
- Dates of Patient's visits, including dispensing of trial medication
- Medical history (including trial indication and concomitant diseases, if applicable)
- Medication history

Proprietary confidential information © 2018 Boehringer Ingelheim International GmbH or one or more of its affiliated companies

- Adverse events and outcome events (onset date (mandatory), and end date (if available))
- Serious adverse events (onset date (mandatory), and end date (if available))
- Concomitant therapy (start date, changes)
- Originals or copies of laboratory results and other imaging or testing results, with proper documented medical evaluation (in validated electronic format, if available)
- Completion of Patient's Participation in the trial (end date; in case of premature discontinuation document the reason for it).
- Prior to allocation of a patient to a treatment into a clinical trial, there must be documented evidence in the source data (e.g. medical records) that the trial participant meets all inclusion criteria and does not meet any exclusion criteria. The absence of records (either medical records, verbal documented feedback of the patient or testing conducted specific for a protocol) to support inclusion/exclusion criteria does not make the patient eligible for the clinical trial.

8.3.2 Direct access to source data and documents

The sponsor will monitor the conduct of the trial by regular on-site monitoring visits and in-house data quality review. The frequency of on-site monitoring will be determined by assessing all characteristics of the trial, including its nature, objective, methodology and the degree of any deviations of the intervention from normal clinical practice.
The Investigator /institution will allow on-site trial-related monitoring, audits, IRB / IEC review and regulatory inspections. Direct access must be provided to the CRF and all source documents/data, including progress notes, copies of laboratory and medical test results, which must be available at all times for review by the CRA, auditor and regulatory inspector (e.g. FDA). The CRA and auditor may review all CRFs and informed consents. The accuracy of the data will be verified by direct comparison with the source documents described in section 8.3.1. The sponsor will also monitor compliance with the protocol and ICH GCP.
An adaptive approach to clinical trial monitoring will be utilised. The sponsor will perform a risk assessment of the trial to determine the extent and nature of monitoring required in order to ensure the reliability and robustness of the results. Regular review of risk reports will provide sponsor oversight during trial conduct and direct monitoring activities to the areas of greatest risk which have the most potential impact to subject safety and data quality.
The Investigator /institution will allow on-site trial-related monitoring, audits, IRB / IEC review and regulatory inspections. Direct access should be granted to all source documents (paper and e-records) including progress notes, copies of laboratory and medical test results. The CRA and auditor may review all CRFs and informed consents. The accuracy of the data will be verified by direct comparison with the source documents described in section 8.3.1. The sponsor will also monitor compliance with the protocol and ICH GCP.

Proprietary confidential information © 2018 Boehringer Ingelheim International GmbH or one or more of its affiliated companies

8.3.3 Storage period of records

Trial site(s):
The trial site(s) must retain the source and essential documents (including ISF) according to the national or local requirements (whatever is longer) valid at the time of the end of the trial.
Sponsor:
The sponsor must retain the essential documents according to the sponsor's SOPs.

8.4 EXPEDITED REPORTING OF ADVERSE EVENTS

BI is responsible to fulfil their legal regulatory reporting obligation and in accordance to the requirements defined in this CTP.

8.5 STATEMENT OF CONFIDENTIALITY AND PATIENT PRIVACY

Individual patient data obtained as a result of this trial is considered confidential and disclosure to third parties is prohibited with the exceptions noted below and in 5.5.4. Patient privacy will be ensured by using patient identification code numbers.
Data protection and data security measures are implemented for the collection, storage and processing of patient data in accordance with the principles 6 and 12 of the WHO GCP handbook.
Treatment data may be given to the patient's personal physician or to other appropriate medical personnel responsible for the patient's welfare. Data generated as a result of the trial need to be available for inspection on request by the participating physicians, the sponsor's representatives, by the IRB / IEC and the regulatory authorities.

8.6 TRIAL MILESTONES

The **start of the trial** is defined as the date when the first patient in the whole trial signs informed consent. **The end of the trial** is defined as the date of the last visit of the last patient in the whole trial ("Last Patient Out"). The "**Last Patient Drug Discontinuation"** (LPDD) date is defined as the date on which the last patient at an individual trial site ends trial medication (as scheduled per protocol or prematurely). Individual Investigators will be notified of SUSARs occurring with the trial medication until 30 days after LPDD at their site.
Early termination of the trial is defined as the premature termination of the trial due to any reason before the end of the trial as specified in this protocol.
Temporary halt of the trial is defined as any unplanned interruption of the trial by the sponsor with the intention to resume it.
Suspension of the trial is defined as an interruption of the trial based on a Health Authority request.
The IEC / competent authority in each participating EU member state will be notified about the trial milestones according to the respective laws.
A final report of the clinical trial data will be written only after all patients have completed the trial in all countries (EU or non-EU) to incorporate and consider all data in the report.

Proprietary confidential information © 2018 Boehringer Ingelheim International GmbH or one or more of its affiliated companies

The sponsor will submit to the EU database a summary of the final trial results within one year from the end of a clinical trial as a whole, regardless of the country of the last patient (EU or non-EU).

Proprietary confidential information © 2018 Boehringer Ingelheim International GmbH or one or more of its affiliated companies

9. REFERENCES

9.1 PUBLISHED REFERENCES

R10-1424 Pinheiro J, Bornkamp B, Bretz F. Design and analysis of dose-finding studies combining multiple companrisons and modelling procedures. J Biopharm Stat. 2006. 16(5): 639–656.

P13-02280 Torres DM, Williams CD, Harrison SA. Features, diagnosis, and treatment of nonalcoholic fatty liver disease. Clin Gastroenterol Hepatol. 2012. 10(8): 837-858.

R10-4848 Common terminology criteria for adverse events (CTCAE): version 4.0 (NIH publication no. 09-5410, published: May 28, 2009 (v4.03: June 14, 2010), revised June 2010, reprinted June 2010). http://evs.nci.nih.gov/ftp1/CTCAE/CTCAE_4.03_2010-06-14_QuickReference_8.5x11.pdf. 2010.

R15-1863 Inoue T, Morita M, Tojo T, Nagashima A, Moritomo A, Miyake H. Novel1H-imidazol-2-amine derivatives as potent and orally active vascular adhesion protein-1 (VAP-1) inhibitors for diabetic macular edema treatment. Bioorg Med Chem. 2013. 21(13): 3873-3881.

R15-1868 Foot JS, Deodhar M, Turner CI, et al. The discovery and development of selective 3-fluoro-4-aryloxyallylamine inhibitors of the amine oxidase activity of semicarbazide-sensitive amine oxidase/vascular adhesion protein-1 (SSAO/VAP-1). Bioorg Med Chem Lett. 2012. 22(12): 3935-3940.

R15-2803 Stolen CM, Marttila-Ichihara F, Koskinen K, et al. Absence of the endothelial oxidase AOC3 leads to abnormal leukocyte traffic in vivo. Immunity. 2005. 22(1): 105-115.

R15-5365 Weston CJ, Shepherd EL, Claridge LC et al. Vascular adhesion protein-1 promotes liver inflammation and drives hepatic fibrosis. J Clin Invest. 2015. 125(2): 501-520.

R15-5652 Bonder CS, Norman MU, Swain MG, et al. Rules of recruitment for Th1 and Th2 lymphocytes in inflamed liver: a role for alpha-4 integrin and vascular adhesion protein-1. Immunity. 2005. 23: 153-163.

R15-5654 Koskinen K, Vainio PJ, Smith DJ, et al. Granulocyte transmigration through the endothelium is regulated by the oxidase activity of vascular adhesion protein-1 (VAP-1). Blood. 2004. 103(9): 3388-3395.

Proprietary confidential information © 2018 Boehringer Ingelheim International GmbH or one or more of its affiliated companies

R15-5697 Karim S, Liaskou E, Fear J, Garg A, Reynolds G, Claridge L, et al. Dysregulated hepatic expression of glucose transporters in chronic disease: contribution of semicarbazide-sensitive amine oxidase to hepatic glucose uptake. Am J Physiol. 2014. 307(12): G1180-G1190.

R15-6056 O'Rourke AM, Wang EY, Miller A, Podar EM, et al. Anti-inflammatory effects of LJP 1586 [Z-3-fluoro-2-(4-methoxybenzyl)allylamine hydrochloride], an amine-based inhibitor of semicarbazide-sensitive amine oxidase activity. J Pharmacol Exp Ther. 2008. 324(2): 867-875.

R15-6057 Murphy SL, Xu J, Kochanek KD. Deaths: final data for 2010. Nat Vital Stat Rep. 2013. 61(4): 1-117.

R15-6058 Merinen M, Irjala H, Salmi M, Jaakkola I, Hanninen A, Jalkanen S. Vascular adhesion protein-1 is involved in both acute and chronic inflammation in the mouse. Am J Pathol. 2005. 166(3): 793-800.

R15-6059 Lee WY, Salmi M, Kelly MM, Jalkanen S, Kubes P. Therapeutic advantage of anti-VAP-1over anti-α4 integrin antibody in concanavalin A-induced hepatitis. Hepatology. 2013. 58(4): 1413-1423.

R15-6044 Chalasani N, Younossi Z, Lavine JE, et al. The diagnosis and management of non-alcoholic fatty liver disease: practice guideline by the American Association for the Study of Liver Diseases, American College of Gastroenterology, and the American Gastroenterological Association. Hepatology. 2012. 55(6): 2005-2023.

R15-6045 Marttila-Ichihara F, Smith DJ, Stolen C, et al. Vascular amine oxidases are needed for leukocyte extravasation into inflamed joints in vivo. Arthritis Rheum. 2006. 54(9): 2852-2862.

R15-6046 Lalor PF, Edwards S, McNab G, et al. Vascular adhesion protein-1 mediates adhesion and transmigration of lymphocytes on human hepatic endothelial cells. J Immunol. 2002. 169(2): 983-992.

R15-6047 Salter-Cid LM, Wang E, O'Rourke AM, et al. Anti-inflammatory effects of inhibiting the amine oxidase activity of semicarbazide-sensitive amine oxidase. J Pharmacol Exp Ther. 2005. 315(2): 553-562.

R15-6048 Xu HL, Salter-Cid L, Linnik MD, Wang EY, Paisansathan C, Pelligrino DA. Vascular adhesion protein-1 plays an important role in postischemic inflammation and neuropathology in diabetic, estrogen-treated ovariectomized female rats subjected to transient forebrain ischemia. J Pharmacol Exp Ther. 2006. 317(1):19-29.

Proprietary confidential information © 2018 Boehringer Ingelheim International GmbH or one or more of its affiliated companies

R15-6070 Dharel N, Fuchs M. Nonalcoholic fatty liver disease - a major public health challenge for the 21st century. JSM Gastroenterol Hepatol. 2014. 2(2): 1018.

R15-5801 Aalto K, Maksimow M, Juonala M, et al. Soluble vascular adhesion protein- 1 correlates with cardiovascular risk factors and early atherosclerotic manifestations. Arterioscler Thromb Vasc. Biol. 2012. 32: 523-532.

R15-5774 Li HY, Jiang YD, Chang TJ, et al. Serum vascular adhesion protein-1 predicts 10-year cardiovascular and cancer mortality in individuals with type 2 diabetes. Diabetes. 2011. 60(3): 993-999.

R15-5773 Li HY, Wei JN, Lin MS, et al. Serum vascular adhesion protein-1 is increased in acute and chronic hyperglycemia. Clinica Chimica Acta. 2009. 404: 149-153.

R15-5766 Lin MS, Li HY, Wei JN, et al. Serum vascular adhesion protein-1 is higher in subjects with early stages of chronic kidney disease. Clin Biochem. 2008. 41: 1362-1367.

R15-5767 Madej A, Reich A, Orda A, Szepietowski JC. Expression of Vascular Adhesion Protein-1 in Atopic Eczema. Int Arch Allergy Immunol. 2006. 139(2): 114-121.

P15-12198 Kurkijärvi R, Yegutkin GG, Gunson BK, Jalkanen S, Salmi M, Adams DH. Circulating soluble vascular adhesion protein-1 accounts for the increased serum monoamine oxidase activity in chronic liver disease. Gastroenterology. 2000. 119(4): 1096-1103.

R15-5771 Kurkijärvi R, Adams DH, Leino R, Mottonen T, Jalkanen S, Salmi M. Circulating form of human vascular adhesion protein-1 (VAP-1): increased serum levels in inflammatory liver diseases. J. Immunol. 1998. 161: 1549-1557.

R15-5772 Kurkijärvi R, Jalkanen S, Isoniemi H, Salmi M. Vascular adhesion protein-1 (VAP-1) mediates lymphocyte-endothelial interactions in chronic kidney rejection. Eur J Immunol. 2001. 31: 2876-2884.

R15-5802 Boomsma F, van den Meiracker AH, Winkel S, et al. Circulating semicarbazide-sensitive amine oxidase is raised both in type I (insulindependent), in type II (non-insulin-dependent) diabetes mellitus and even in childhood type I diabetes at first clinical diagnosis. Diabetologia. 1999. 42: 233-237.

Proprietary confidential information © 2018 Boehringer Ingelheim International GmbH or one or more of its affiliated companies

R16-5301	McPherson S, Hardy T, Henderson E, Burt AD, Day CP, Anstee QM. Evidence of NAFLD progression from steatosis to fibrosing-steatohepatitis using paired biopsies: implications for prognosis and clinical management. J Hepatol. 2015. 62: 1148-1155.
R16-5309	Chalasani N, Regev A. Drug induced liver injury in patients with preexisting chronic liver disease in drug development – how to identify and manage? Gastroenterology. 2016.
R16-5489	Ratziu V, Harrison SA, Francque S, Bedossa P, Lehert P, Serfaty L, et al, GOLDEN-505 Investigator Study Group<br>Elafibranor, an agonist of the peroxisome proliferator-activated receptor-alpha and -delta, induces resolution of nonalcoholic steatohepatitis without fibrosis worsening.<br>Gastroenterology 150 (5), 1147 - 1159 (2016)
R18-2779	Safadi R, Konikoff FM, Mahamid M, Zelber-Sagi S, Halpern M, Gilat T, et al, FLORA Group<br>The fatty acid-bile acid conjugate Aramchol reduces liver fat content in patients with nonalcoholic fatty liver disease.<br>Clin Gastroenterol Hepatol 12 (12), 2085 - 2091 (2014)
R18-2781	Friedman SL, Ratziu V, Harrison SA, Abdelmalek MF, Aithal GP, Caballeria J, et al<br>A randomized, placebo-controlled trial of cenicriviroc for treatment of nonalcoholic steatohepatitis with fibrosis.<br>Hepatology 67 (5), 1754 - 1767 (2018)

Proprietary confidential information © 2018 Boehringer Ingelheim International GmbH or one or more of its affiliated companies

10. APPENDICES

10.1 PHARMACOKINETIC AND BIOMARKER SAMPLING TIME POINTS

Table 10.1:1 Blood sampling scheme for pharmacokinetic and pharmacodynamic/biomarker blood samples

Visit No.	Day	Time point	Time rel. to drug admin. [h]	CRF Time / planned time [h:min]	PK samples	AOC3 activity / concentration
2	1	Prior to first BI 1467335 dose (baseline)	-0.5	-0:30	X	X
		BI 1467335 administration	0	0:00		
			0.25	0:15	X	
			0.5	0:30	X	X
			1	1:00	X	X
			1.5	1:30	X	
			2	2:00	X	X
			3	3:00	X	X
			6	6:00	X	X
			8	8:00	X	X
3	15		-0.5	335:30	X	X
		BI 1467335 administration	0	336:00		
			1	337:00	X	
			1.5	337:30	X	

Proprietary confidential information © 2018 Boehringer Ingelheim International GmbH or one or more of its affiliated companies

Table 10.1:1 continued Blood sampling scheme for pharmacokinetic and pharmacodynamic/biomarker blood samples

Visit No.	Day	Time point	Time rel. to drug admin. [h]	CRF Time / planned time [h:min]	PK samples	AOC3 activity / concentration
4	29		-0.5	671:30	X	X
		BI 1467335 administration	0	672:00		
			0.25	672:15	X	
			0.5	672:30	X	X
			1	673:00	X	X
			1.5	673:30	X	
			2	674:00	X	X
			3	675:00	X	X
			6	678:00	X	X
			8	680:00	X	X
5	43		-0.5	1007:30	X	X
		BI 1467335 administration	0	1008:00		
			1	1009:00	X	
			1.5	1009:30	X	
6	57		-0.5	1343:30	X	X
		BI 1467335 administration	0	1344:00		
			1	1345:00	X	
			1.5	1345:30	X	

Proprietary confidential information © 2018 Boehringer Ingelheim International GmbH or one or more of its affiliated companies

Table 10.1:1 continued Blood sampling scheme for pharmacokinetic and pharmacodynamic/biomarker blood samples

Visit No.	Day	Time point	Time rel. to drug admin. [h]	CRF Time / planned time [h:min]	PK samples	AOC3 activity / concentrati on
EOT	85		-0.5	2015:30	X	X
		BI 1467335 administration	0	2016:00		
			0.25	2016:15	X	
			0.5	2016:30	X	X
			1	2017:00	X	X
			1.5	2017:30	X	
			2	2018:00	X	X
			3	2019:00	X	X
			6	2022:00	X	X
			8	2024:00	X	X
			24	2040:00	X	X
EOT + 28	113		672	2688:00		X

Proprietary confidential information © 2018 Boehringer Ingelheim International GmbH or one or more of its affiliated companies

Proprietary confidential information © 2018 Boehringer Ingelheim International GmbH or one or more of its affiliated companies

10.3 INCREASED LIVER ENZYMES PROCEDURE

Please also refer to section 3.3.4.1.1.

Figure 10.3: 1 study- specific procedures in case of further increased liver enzymes after randomisation.

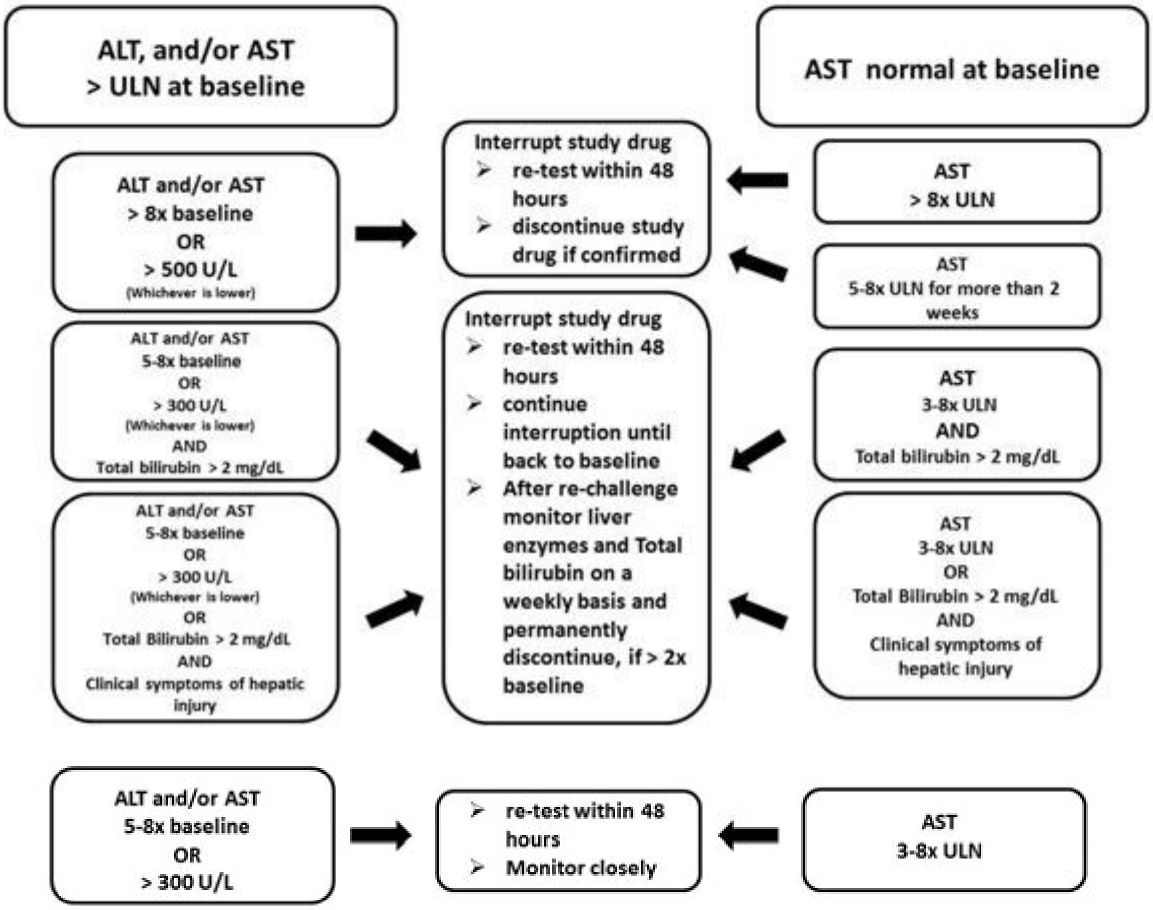

Proprietary confidential information © 2018 Boehringer Ingelheim International GmbH or one or more of its affiliated companies

Proprietary confidential information © 2018 Boehringer Ingelheim International GmbH or one or more of its affiliated companies

Proprietary confidential information © 2018 Boehringer Ingelheim International GmbH or one or more of its affiliated companies

Proprietary confidential information © 2018 Boehringer Ingelheim International GmbH or one or more of its affiliated companies

Proprietary confidential information © 2018 Boehringer Ingelheim International GmbH or one or more of its affiliated companies

Proprietary confidential information © 2018 Boehringer Ingelheim International GmbH or one or more of its affiliated companies

Proprietary confidential information © 2018 Boehringer Ingelheim International GmbH or one or more of its affiliated companies

Proprietary confidential information © 2018 Boehringer Ingelheim International GmbH or one or more of its affiliated companies

Proprietary confidential information © 2018 Boehringer Ingelheim International GmbH or one or more of its affiliated companies

Proprietary confidential information © 2018 Boehringer Ingelheim International GmbH or one or more of its affiliated companies

Proprietary confidential information © 2018 Boehringer Ingelheim International GmbH or one or more of its affiliated companies

Proprietary confidential information © 2018 Boehringer Ingelheim International GmbH or one or more of its affiliated companies

Proprietary confidential information © 2018 Boehringer Ingelheim International GmbH or one or more of its affiliated companies

Proprietary confidential information © 2018 Boehringer Ingelheim International GmbH or one or more of its affiliated companies

Proprietary confidential information © 2018 Boehringer Ingelheim International GmbH or one or more of its affiliated companies

Proprietary confidential information © 2018 Boehringer Ingelheim International GmbH or one or more of its affiliated companies

Proprietary confidential information © 2018 Boehringer Ingelheim International GmbH or one or more of its affiliated companies

Supplementary Note 3. Statistical analysis plan.

Boehringer Ingelheim

TRIAL STATISTICAL ANALYSIS PLAN **c15128276-02**

**BI Trial No.:**	1386-0004
**Title:**	A multi-centre, double-blind, parallel-group, randomised, placebo controlled phase II a study to investigate safety, tolerability, pharmacodynamics, and pharmacokinetics of different doses of orally administered BI 1467335 during a 12-week treatment period compared to placebo in patients with clinical evidence of NASH.<br>Including Protocol Amendment 1 [c08980589-01], amendment 2 [c08980589-03-02], amendment 3 [c08980589-03], amendment 4 [c08980589-04]
**Investigational Product(s):**	BI 1467335
**Responsible trial statistician(s):**	Phone:<br>Fax:
**Date of statistical analysis plan:**	10 AUG SIGNED
**Version:**	FINAL
	**Page 1 of 50**

Proprietary confidential information
© 2018 Boehringer Ingelheim International GmbH or one or more of its affiliated companies. All rights reserved.
This document may not - in full or in part - be passed on, reproduced, published or otherwise used without prior written permission.

Proprietary confidential information © 2018 Boehringer Ingelheim International GmbH or one or more of its affiliated companies

1. TABLE OF CONTENTS

TITLE PAGE ... 1
1. TABLE OF CONTENTS ... 2
LIST OF TABLES ... 4
2. LIST OF ABBREVIATIONS ... 5
3. INTRODUCTION ... 8
4. CHANGES IN THE PLANNED ANALYSIS OF THE STUDY ... 9
5. ENDPOINTS ... 10
5.1 PRIMARY ENDPOINT ... 10
5.2 SECONDARY ENDPOINTS ... 10
5.2.1 Key secondary endpoint ... 10
5.2.2 Secondary endpoints ... 10
5.3.2 Safety endpoints ... 11
5.4 OTHER VARIABLES ... 15
6. GENERAL ANALYSIS DEFINITIONS ... 16
6.1 TREATMENTS ... 16
6.2 IMPORTANT PROTOCOL VIOLATIONS ... 17
6.3 PATIENT SETS ANALYSED ... 20
... 22
6.5 POOLING OF CENTRES ... 23
6.6 HANDLING OF MISSING DATA AND OUTLIERS ... 23
6.6.1 Definition of criteria for censoring ... 23
6.6.2 Endpoint specific handling of missing data ... 23
6.7 BASELINE, TIME WINDOWS AND CALCULATED VISITS ... 24
7. PLANNED ANALYSIS ... 29
7.1 DEMOGRAPHIC AND OTHER BASELINE CHARACTERISTICS ... 30
7.2 CONCOMITANT DISEASES AND MEDICATION ... 30
7.3 TREATMENT COMPLIANCE ... 30
7.4 PRIMARY ENDPOINT ... 31
7.4.1 Primary analysis ... 31
7.5 SECONDARY ENDPOINTS ... 32
7.5.1 Key secondary endpoint ... 32
7.5.2 Secondary endpoints ... 32
... 34
... 34
... 36

Proprietary confidential information © 2018 Boehringer Ingelheim International GmbH or one or more of its affiliated companies
.... 36
.... 37
.... 37
7.7 EXTENT OF EXPOSURE 38
7.8 SAFETY ANALYSIS 38
7.8.1 Adverse events 38
7.8.2 Laboratory data 39
7.8.3 Vital signs 40
7.8.4 ECG 40

8. REFERENCES 43
.... 45
.... 45
.... 45
.... 46
.... 46
.... 48
.... 48
.... 48
.... 48
.... 48
.... 49
10. HISTORY TABLE 50

Proprietary confidential information © 2018 Boehringer Ingelheim International GmbH or one or more of its affiliated companies

LIST OF TABLES

Table 6.1: 1 Treatment descriptions........16
Table 6.1: 2 Analysing periods (same for all treatment groups) 16
Table 6.2: 1 Important protocol violations 17
Table 6.3: 1 Patient sets analysed........21
Table 6.7: 1 Time schedule of 12-lead ECG recordings........25
Table 6.7: 2 Visit window for centralized ECG endpoints........25
Table 6.7: 3 Visit window for AOC3........26
Table 6.7: 4 Time window for AOC3........26
Table 6.7: 5 Visit window for secondary and further biomarker endpoints* 27
Table 6.7: 6 Visit window for safety endpoints........27
Table 7.5.2: 1 Contrast coefficients 33
Table 10: 1 History table 50

Proprietary confidential information © 2018 Boehringer Ingelheim International GmbH or one or more of its affiliated companies

2. LIST OF ABBREVIATIONS

Term	Definition / description
γ-GT	Gamma-Glutamyltransferase
AE	Adverse event
ALT	Alanine Aminotransferase
AOC3	Amine oxidase copper-containing 3
AP	Alkaline Phosphatase
AST	Aspartate Aminotransferase
BP	Blood pressure
BRPM	Blinded report planning meeting
CTCAE	Common Terminology Criteria for Adverse Events
CTP	Clinical Trial Protocol
CTR	Clinical Trial Report
CV [%]	Arithmetic coefficient of variation in %
DBLM	Data base lock meeting
DBP	Diastolic blood pressure
DM&SM	Boehringer Ingelheim Data Management and Statistics Manual
DRA	Drug Regulatory Affairs
DMG	Dictionary Maintenance Group
ECG	Electrocardiogram
ECGS	ECG analysis set
ELF	Enhanced Liver Fibrosis
EMEA	European Agency for the Evaluation of Medicinal Products
FAS	Full Analysis Set
HDL	High Density Lipoprotein
HR [beats/min]	Heart rate in beats per minute

Proprietary confidential information © 2018 Boehringer Ingelheim International GmbH or one or more of its affiliated companies

Term	Definition / description
HV	Healthy volunteer
ICH	International Conference on Harmonisation
IPV	Important Protocol Violation
LDL	Low Density Lipoprotein
MCPMod	Multiple Comparison Procedure Modelling
MedDRA	Medical Dictionary for Regulatory Activities
MMRM	Mixed Effect Model Repeated Measurement
MQRM	Medical Quality Review Meeting
NAFLD	Non-alcoholic fatty liver disease
NASH	Non-alcoholic steatohepatitis
PK	Pharmacokinetics
PKECGS	PK ECG set
PPS	Per protocol set
PR interval	ECG interval from the onset of P wave to the beginning of the QRS
PSTAT	Project Statistician
PT	Preferred term
PV	Protocol violation
QD	quaque die (once a day)
QRS complex	Combination of the Q, R, and S waves
QT interval	ECG interval from the beginning of the QRS complex to the end of the T wave
QTcB [msec]	QT interval, heart rate corrected according to Bazetts formula
QTcF [msec]	QT interval, heart rate corrected according to Fridericias formula
QTcN [msec]	QT interval, heart rate corrected according to study population formula
REP	Residual effect period
RR interval	ECG interval from the peak of the R wave to the peak of the subsequent R wave
RPM	Report Planning Meeting

Proprietary confidential information © 2018 Boehringer Ingelheim International GmbH or one or more of its affiliated companies

Term	Definition / description
RS	Randomised set
SA	Statistical analysis
SBP	Systolic blood pressure
SD	Standard deviation
SMQ	Standardised MedDRA query
SOC	System organ class
TCM	Trial Clinical Monitor
ToC	Table of contents
TMW	Trial Medical Writer
TS	Treated set
TSAP	Trial statistical analysis plan

Proprietary confidential information © 2018 Boehringer Ingelheim International GmbH or one or more of its affiliated companies

3. INTRODUCTION

As per ICH E9 (1), the purpose of this document is to provide a more technical and detailed elaboration of the principal features of the analysis described in the protocol, and to include detailed procedures for executing the statistical analysis of the primary and secondary variables and other data.

This TSAP assumes familiarity with the Clinical Trial Protocol (CTP), including Protocol Amendments. In particular, the TSAP is based on the planned analysis specification as written in CTP Section 7 “Statistical Methods and Determination of Sample Size”. Therefore, TSAP readers may consult the CTP for more background information on the study, e.g., on study objectives, study design and population, treatments, definition of measurements and variables, planning of sample size, randomisation.

Study data will be stored in a trial database within the medidata RAVE (BRAVE) system.

The statistical analyses will be performed within the validated working environment CARE (Clinical data Analysis and Reporting Environment), including SAS_{TM} (current Version 9.4, by SAS Institute Inc., Cary, NC, USA), and a number of SAS_{TM}-based tools (e.g., macros for the analyses of adverse event (AE) data or laboratory data; Report Appendix Generator system (RAGe) for compilation/formatting of the clinical trial report (CTR) appendices). R version 3.3.2 or later with “DoseFinding” package [R15-2001] will be used for analysis based on MCPMod.

Proprietary confidential information © 2018 Boehringer Ingelheim International GmbH or one or more of its affiliated companies

4. CHANGES IN THE PLANNED ANALYSIS OF THE STUDY

No changes to the planned analysis according to the CTP have been made.

Proprietary confidential information © 2018 Boehringer Ingelheim International GmbH or one or more of its affiliated companies

5. ENDPOINTS

General remarks with regard to naming convention and its derivation:

- Absolute value: $X_{on\text{-}treatment}$ or $X_{baseline}$
- Absolute change from baseline: $X_{on\text{-}treatment} - X_{baseline}$
- Change from baseline in %: $\frac{Xon\text{-}treatment\text{-}\ Xbaseline}{X_{baseline}} * 100$
- Relative to baseline in %: $\frac{Xon\text{-}treatment}{X_{baseline}} * 100$

5.1 PRIMARY ENDPOINT

CTP Section 5.1.1:
The primary endpoint is the plasma amine oxidase copper-containing 3 (AOC3) activity relative to baseline in %, 24 h post dose, after 12 weeks of treatment. The baseline is defined as the last AOC3 activity measurement prior to administration of any randomised study medication.

5.2 SECONDARY ENDPOINTS

5.2.1 Key secondary endpoint

There are no key secondary endpoints in this trial.

5.2.2 Secondary endpoints

CTP Section 5.1.2:
Safety and tolerability will be assessed based on the number (%) of subjects with adverse reactions[displayed as drug-related adverse events].

The secondary biomarker endpoints will be assessed based on the

- *relative ALT change from baseline after 12 weeks of treatment*
- *relative AST change from baseline after 12 weeks of treatment*
- *relative AP change from baseline after 12 weeks of treatment*
- *relative γ-GT change from baseline after 12 weeks of treatment (GGT)*
- *relative caspase cleaved cytokeratin 18 (M30) change from baseline after 12 weeks of treatment (CK-18 Caspase)*
- *relative total cytokeratin 18 (M65) change from baseline after 12 weeks of treatment (CK-18 Total)*

The relative change from baseline in % will be described as “relative to baseline in %” or in short as “R2Base %”.

Proprietary confidential information © 2018 Boehringer Ingelheim International GmbH or one or more of its affiliated companies

5.3.2 Safety endpoints

12-lead ECG endpoints

For the definition of baseline and a summary of time points please refer to Section 6.7.

Quantitative ECG endpoints:

The following quantitative ECG endpoints will be determined for the ECG variables QTcF, QT, HR, PR, QRS, RR and QTcB derived as described in Additional Section 9.1:

- absolute values (per time point)
- changes from baseline (per time point)
- percent changes from baseline (per time point; for HR, PR, QRS)

Categorical ECG endpoints

The following categorical ECG endpoints will be determined based on the quantitative ECG endpoints:

New onset (meaning that this or a higher category was not present any time at baseline) of maximum QTcF interval > 450 to 480 msec, > 480 to 500 msec, or > 500 msec on treatment. For assignment of a particular patient to one of the above categories, all time points on-treatment (refer to Table 6.7: 1) will be considered.

- Maximum change from baseline in QT interval ≤ 60 msec, or > 60 msec on treatment
- Maximum change from baseline in QTcF interval ≤ 30 msec, > 30 to ≤ 60 msec, or > 60 msec on treatment

The occurrence of any of the following will be viewed as “notable findings”:

- New onset (not present any time at baseline) of uncorrected QT interval > 500 msec at any time on treatment

Proprietary confidential information © 2018 Boehringer Ingelheim International GmbH or one or more of its affiliated companies

If baseline is missing, any occurrence of QT interval > 500 msec at any time on treatment will be a notable finding

- New onset of QTcF interval > 500 msec at any time on treatment
  If baseline is missing, any occurrence of QTcF interval > 500 msec at any time on treatment will be a notable finding
- Increase in QTcF interval from baseline by > 60 msec at any time on treatment
- Increase in HR from baseline by ≥ 25%, when corresponding on-treatment value of HR is > 100 bpm, or decrease in HR by ≥ 25%, when corresponding on-treatment value of HR is < 50 bpm, at any time on treatment
- Increase in the PR interval from baseline by ≥ 25%, when corresponding on-treatment value of PR interval is > 200 msec, at any time on treatment
- Increase in the QRS complex from baseline by ≥ 10%, when corresponding on-treatment value of QRS complex is > 110 msec, at any time on treatment

Categorical endpoints will also include morphological (i.e. qualitative) findings that might be attributable to treatment. In particular, new onset of findings not present at baseline with regard to e.g. rhythm, conduction, ST segment changes, T and U wave abnormalities and myocardial infarction will be explored.

Vital signs and body weight

For the Vital Signs variables:

- Systolic blood pressure
- Diastolic blood pressure
- Pulse rate
- Respiratory rate

And the body weight variables:

- BMI
- Body weight
- Waist circumference
- Hip circumference
- Hip/Waist ratio

The following quantitative vital signs and body weight endpoints will be determined

- absolute values (per time point)
- changes from baseline (per time point)

Proprietary confidential information © 2018 Boehringer Ingelheim International GmbH or one or more of its affiliated companies

Proprietary confidential information © 2018 Boehringer Ingelheim International GmbH or one or more of its affiliated companies

Proprietary confidential information © 2018 Boehringer Ingelheim International GmbH or one or more of its affiliated companies

Proprietary confidential information © 2018 Boehringer Ingelheim International GmbH or one or more of its affiliated companies

6. GENERAL ANALYSIS DEFINITIONS

6.1 TREATMENTS

For basic trial information on treatments to be administered, assignment of a dose group, and selection of doses, see CTP Section 4.

Table 6.1: 1 Treatment descriptions

Long Name	Short Name
Placebo	Placebo
BI 1467335 1 mg QD	1 mg QD
BI 1467335 3 mg QD	3 mg QD
BI 1467335 6 mg QD	6 mg QD
BI 1467335 10 mg QD	10 mg QD

Patients will be analysed as randomised for safety and efficacy analyses.

Table 6.1: 2 Analysing periods (same for all treatment groups)

Analysing Treatment Period	Start time	Stop time
Screening	0:00 h on day of informed consent	first treatment administration
On-treatment	first treatment administration	Individual trial termination date

Displays of AEs, laboratory tests and vital signs will be presented separately for the following treatments during the “On-treatment” phase:

- Placebo
- BI 1467335 1 mg QD
- BI 1467335 3 mg QD
- BI 1467335 6 mg QD
- BI 1467335 10 mg QD

AEs:
Two types of AE displays will be provided in the report:

A) Section 15.3 and Appendix 16.1.13.1.8 (for ClinicalTrials.gov and EudraCT only) of the CTR displays:

In these displays, the on-treatment phase will be analysed (labelled with the name of the study treatment (short name)). Screening period will not be included in this analysis.

Proprietary confidential information © 2018 Boehringer Ingelheim International GmbH or one or more of its affiliated companies

B) Section 15.4 and Appendix 16.1.13.1.8 (except for ClinicalTrials.gov and EudraCT) of the CTR displays:

- Screening (labelled "**Screening**")
- On-treatment (labelled with the name of the study treatment (label with short name))

In Section 16.1.13.1.8 AE tables, the following totals will be provided in addition:

- a total over all active treatments during on-treatment ("**Total BI**")

Laboratory tests:
Laboratory values are displayed for

- Baseline
- On-treatment

Vital signs:
Vital signs and 12-lead ECG values are displayed for

- Baseline
- On-treatment

For more details refer to Section 6.7.

More details on the technical implementation of these analyses are provided in the Analysis Data Set Plan (ADS Plan) of this TSAP.

6.2 IMPORTANT PROTOCOL VIOLATIONS

A protocol violation (PV) is important if it affects the rights or safety of the study patients, or if it can potentially influence the primary outcome measurement(s) in a non-negligible way.

A list of important PVs (IPVs) is given in Table 6.2: 1. Important PVs will be reviewed at Medical Quality Review Meetings (MQRMs) conducted periodically during the trial. A list of protocol deviations will be discussed at the Blinded Report Planning Meetings (BRPMs).

If the data show other important PVs, this table will be supplemented accordingly at MQRMs or BRPMs or through team review of the manual PV log. The decision whether a patient will be excluded from the analysis will be made at the final BRPM prior to Database Lock (DBL).

Table 6.2: 1 Important protocol violations

Category / Code		Description	Comment	Excluded from
**A**	**Entrance criteria not met**			
**A1**	**Target indication not met**			
	A1.01	Clinical evidence of NASH	Inclusion criterion checked	PPS
**A2**	**Inclusion criteria not met**			

Proprietary confidential information © 2018 Boehringer Ingelheim International GmbH or one or more of its affiliated companies

Table 6.2: 1 Important protocol violations (continued)

Category / Code		Description	Comment	Excluded from
	A2.01	ALT >1.5 ULN at screening and ALT >1.25 ULN in a local lab within 1 week to 3 months prior screening	Inclusion criterion checked	PPS
	A2.02	Age ≥ 18 and ≤75 years at screening	Inclusion criterion checked	None
	A2.03	BMI ≥25kg/m^2 and <45kg/m^2 at screening	Inclusion criterion checked	None
	A2.04	Stable body weight	Inclusion criterion checked	None
	A2.05	Stable concomitant medication (Antidiabetic, anti-obesity, Vitamine E)	Inclusion criterion checked	PPS
	A2.06	Stable concomitant medication (All other)	Inclusion criterion checked	None
	A2.07	For females: use of double barrier contraception	Inclusion criterion checked	None
**A3**	**Exclusion criteria met**			
	A3.01	Current or history of significant alcohol consumption	Exclusion criterion checked	PPS
	A3.02	Prior participation in an interventional NASH trial	Exclusion criterion checked	PPS
	A3.03	Prior or planned bariatric surgery	Exclusion criterion checked	PPS
	A3.04	Drugs associated with liver injury, hepatic steatosis or steatohepatitis prior screening	Exclusion criterion checked	PPS
	A3.05	History of liver cirrhosis or other forms of liver disease	Exclusion criterion checked	PPS
	A3.06	active infection	Exclusion criterion checked	None
	A3.07	Solid liver lesions other than haemangiomas	Exclusion criterion checked	PPS
	A3.08	eGFR <60ml/min/1.73m^2 at screening (or Renal insufficiency or renal impairment (assessed by eGFR))	Exclusion criterion checked	None
	A3.09	ALT >5.0 ULN at screening	Exclusion criterion checked	PPS
	A3.10	Platelet count < 150.000/µL	Exclusion criterion checked	PPS
	A3.11	Bilirubin level > 1.25xULN	Exclusion criterion checked	PPS
	A3.12	Uncontrolled diabetes	Exclusion criterion checked	PPS
	A3.13	Diagnosis of a serious or unstable disease	Exclusion criterion checked	PPS
	A3.14	Prior or planned major surgery	Exclusion criterion checked	PPS
	A3.15	Active, suspected or history of Malignancy	Exclusion criterion checked	None
	A3.16	Use of restricted medication	Exclusion criterion checked	PPS
	A3.17	Previous randomisation in this trial	Exclusion criterion checked	PPS
	A3.18	Currently enrolled in another investigational study	Exclusion criterion checked	PPS
	A3.19	Chronic drug abuse or any condition unsuitable for trial participation	Exclusion criterion checked	PPS
	A3.20	Pregnant, nursing or who plan to become pregnant women	Exclusion criterion checked	None
	A3.21	baseline QT issue or WPW syndrome	Exclusion criterion checked	None
	A3.22	Use of MAO-B inhibitors at screening or planned during study*	Exclusion criterion checked	PPS
	A3.23	Safety concerns by investigator opinion	Exclusion criterion checked	None
**B**	**Informed consent**			
	B1	Informed consent not available	Date or signatory missing	All

Proprietary confidential information © 2018 Boehringer Ingelheim International GmbH or one or more of its affiliated companies

Table 6.2: 1 Important protocol violations (continued)

**Category / Code**		**Description**	**Comment**	**Excluded from**
	B2	Informed consent too late	Date of informed consent not obtained prior to any study related procedure	None
	B5	Informed consent obtained with error		None
**C**	**Trial medication and randomisation**			
**C1**	**Incorrect trial medication**			
	C1.01	No study medication taken	Patient randomised but no study medication taken	TS, FAS, PPS,
	C1.02	Incorrect trial medication taken	Wrong medication taken for more than 20% of the overall treatment duration or for more than 20% of the last visit interval before the primary endpoint assessment (different medication than the patient was randomised to taken i.e. drug kit recorded in eCRF from different treatment group than drug kit assigned by IVRS [automated check PV])	PPS
**C3**	**Non-compliance**			
	C3.01	Non-compliance with study drug intake	Overall study treatment compliance outside 80% and 120% (exclusive) or study treatment compliance below 80% in the last visit interval before primary endpoint assessment.	PPS
**C4**	**Medication code broken**			
	C4.01	Medication code broken at site without just cause	Medication code was broken for no valid reason. Final decision at the DBL meeting based on medical judgement	None Final decision at the DBL meeting.
**D1**	**Concomitant medication**			
	D1.01	Non stable concomitant medication during treatment (Amiodarone, methotrexate, systemic glucocorticoids, tetracyclines, tamoxifen, estrogens (excluding oral contraception), anabolic steroids, valproic acid, Pioglitazone and GLP1-Agonists as well as the initiation of insulin)	Review of eCRF for concomitant medication use. Final decision at the DBL meeting based on medical judgement.	PPS Final decision at the DBL meeting

Proprietary confidential information © 2018 Boehringer Ingelheim International GmbH or one or more of its affiliated companies

Table 6.2: 1 Important protocol violations (continued)

Category / Code		Description	Comment	Excluded from
**D2**	**Prohibited medication use**			
	D2.01	Use of prohibited medication during treatment period	Review of eCRF for prohibited medication. Final decision at the DBL meeting based on medical judgement.	PPS Final decision at the DBL meeting.
**E**	**Missing data****			
	E1.01	No baseline biomarker value (for all of AOC3 activity, ALT, AST, AP, γ-GT, CK-18 caspase and CK-18 total)	No valid baseline biomarker value	FAS, PPS
	E1.02	No on-treatment biomarker value (for all of AOC3 activity, ALT, AST, AP, γ-GT, CK-18 caspase and CK-18 total)	No valid on-treatment biomarker value	FAS, PPS
**G**	**Trial specific**			
	G1.01	Too short treatment duration	at least 8 weeks on treatment	PPS
	G1.02	Pregnant female during trial		None

* Exclusion criteria was deleted in CTP Version 3 (16 March 2018).
**Further missing data beyond the explicitly described in E will not be considered as IPV.

The IPV category B5, D1 and D2 will be a manual IPV and need to be identified at a site level on the manual PV log. All remaining IPVs are programmed automatically.

6.3 PATIENT SETS ANALYSED

The following patient analysis sets are defined for this trial:

- Screened Set (SCR): includes all patients who signed informed consent.
- Randomised Set (RS): includes all patients who were screened for the trial and were randomised to trial treatment, regardless of whether any trial treatment was administered.
- Treated Set (TS): includes all patients who signed informed consent and were treated with at least one dose of the trial medication. The TS is used for safety analyses as well as demographics and baseline characteristics.
- Full Analysis Set (FAS): includes all patients in treated set who had non-missing baseline and at least one non-missing post-baseline and on-treatment measurement on any primary, secondary or further biomarker endpoint. Patients in FAS are analysed according to the intent-to-treat principle.
- Per Protocol Set (PPS): includes all patients from the FAS without IPVs leading to exclusion.
-

Proprietary confidential information © 2018 Boehringer Ingelheim International GmbH or one or more of its affiliated companies

- ECG Set (ECGS): This patient set includes all patients in the TS who do not have artificial cardiac pacemakers and have at least one on-treatment value for at least one ECG endpoint, which is not excluded due to ECG relevant protocol violations. Relevant protocol violations may be e.g. the use of pro-arrhythmic medications. Exclusion of single ECG values due to relevant PVs is to be decided no later than in the BRPM before data base lock and will be documented in the CTR.
- ECG Pharmacokinetic Concentration Set (ECGPCS): This patient set includes all patients from the ECGS who provide at least one pair of a valid drug plasma concentration and a corresponding (i.e. time-matched) ECG endpoint on-treatment to be used in the exposure-response analysis. Since placebo patients will be included in the exposure response analyses with plasma concentrations set to 0, a plasma concentration BLQ is considered a valid drug plasma concentration for placebo patients. The decision about concentration value validity needs to be assessed within the Clinical Pharmacology group. The decision whether a time deviation between PK blood sampling and ECG recording is acceptable (and thus whether the pair of values will be used) is to be made no later than at the BRPM before data base lock.

Table 6.3: 1 Patient sets analysed

	Patient set						
Class of endpoint	SCR	TS	FAS	PPS		ECGS	ECGPCS
Primary and secondary endpoints				x			
Safety endpoints		x					
ECG endpoints						x	x**
Demographic/baseline endpoints		x					
Important protocol violations	x						
Disposition	x						

; ** only for exposure response analysis

Proprietary confidential information © 2018 Boehringer Ingelheim International GmbH or one or more of its affiliated companies

Proprietary confidential information © 2018 Boehringer Ingelheim International GmbH or one or more of its affiliated companies

6.5 POOLING OF CENTRES

This section is not applicable because centre/country is not included in the statistical model.

6.6 HANDLING OF MISSING DATA AND OUTLIERS

Based on the different reasons of patients' data missing for different endpoints, various methods will be used to assess the impact of missing data on the efficacy endpoints of this trial, depending upon the type of the endpoint:

6.6.1 Definition of criteria for censoring

The new introduction or change of concomitant therapies can influence certain study endpoints of interest. To allow the assessment of the impact of such changes, specific efficacy endpoint values after changes of concomitant medication will be set to missing. I.e. there may be the case that there is a an IPV with regard to D1 or D2 which only leads to exclusion of certain measurements within a certain time frame, but not to exclusion of the patient from the analysis set. This will be decided and documented before DBL.

6.6.2 Endpoint specific handling of missing data

Primary endpoint:
Handling of missing AOC3 activity values is covered by using multiple imputation described in more detail in Section 7.3.1 of the CTP. Sensitivity analyses with other methods how to handle missing values are described in Section 7.4.2.

Secondary biomarker endpoints:
Missing values of the secondary biomarker endpoints are directly handled within the applied MMRM model based on the likelihood method under the "missing at random" assumption.

Safety endpoints and other variables:
As already described in the CTP Section 7.5 it is not planned to impute missing values. The only exceptions where imputation might be necessary for safety evaluation are AE dates.

Proprietary confidential information © 2018 Boehringer Ingelheim International GmbH or one or more of its affiliated companies

Missing or incomplete AE dates are imputed according to BI standards (see 001-MCG-156_RD-01 (3)).

ECG variables:
If single cardiac cycles of an ECG (out of the three) are missing, the arithmetic mean for this single ECG will be computed with the reduced (1 or 2) number of cardiac cycles.

For the classification of the on-treatment QTc/QT intervals into 'no new onset' / 'new onset' categories, a missing value is obtained only in case that

i. all on-treatment values are missing and
ii. the baseline value is less than or equal to 500 msec, or missing.

If condition (i) is fulfilled but the baseline value is greater than 500 msec, this case will be categorized as 'no new onset'. If baseline is missing and the maximum on-treatment QTc interval is greater than 450 msec (or 500 msec for QT interval, respectively), this is classified as a 'new onset' in the respective category. If baseline is missing and the maximum QTc interval is less than or equal to 450 msec (or 500 msec for QT interval, respectively), this will be categorized as 'no new onset'. If baseline is missing, a QTc/QT interval > 500 msec at any time on treatment will be a notable finding.

For patients on active drug, missing plasma concentration values with 'BLQ' in the comment field will be replaced by ½ LLOQ.

6.7 BASELINE, TIME WINDOWS AND CALCULATED VISITS

Unless a different definition is provided below, baseline values are the last measurements taken prior to the first administration of trial treatment. If this value is not available, the measurement at the screening visit is used.

Centralized 12-lead ECG
There will be a centralised evaluation of all 12-lead ECG recordings at the time points specified in the Table 6.7: 1 below for the first of the three replicate ECGs at a single assessment time, except for baseline where triplicates are used. The baseline value of an ECG variable is then defined as the mean of the triple ECG measurements prior to drug administration:

Proprietary confidential information © 2018 Boehringer Ingelheim International GmbH or one or more of its affiliated companies

Table 6.7: 1 Time schedule of 12-lead ECG recordings

**Visit**	**Planned Day**	**Planned time relative to drug administration [hh:mm]**	**Study phase**
1	Within -28 to-7 days before drug administration		Baseline
2	1	1.5	On-treatment
3	15	1.5	
4	29	1.5	
5	43	1.5	
6	57	1.5	
EOT/ ED	85	1.5	
FU	EOT+28	-	

For the exposure response analyses, pair of ECG variables and corresponding plasma concentrations will be built using the same planned time points, e.g. HR change from baseline and plasma sample taken at planned time 0:30 will build one pair. Whether a time deviation between PK sample and corresponding ECG extraction is too big and the pair has to be excluded or matched to another time point will be decided no later than at the BRPM/DBLM.

For quantitative analysis except exposure response analysis, data will be presented according to visit window described below.

Table 6.7: 2 Visit window for centralized ECG endpoints

		**Interval Definition**		
**Visit**	**Planned day**	**From day**	**To day**	**Visit description**
1	-28 to -7	-28	-1	Baseline
2	1	1	1	Day 1
3	15	2	22	Day 15 (week 2)
4	29	23	36	Day 29 (week 4)
5	43	37	50	Day 43 (week 6)
6	57	51	71	Day 57 (week 8)
EOT	85	72	Date of drug intake in EOT visit+1	Day 85 (week 12)
FU	113	Date of drug intake in EOT visit+2	Last assessment date	Day 113 (FU)

Proprietary confidential information © 2018 Boehringer Ingelheim International GmbH or one or more of its affiliated companies

Primary endpoint:

Table 6.7: 3 Visit window for AOC3

		**Interval Definition**		
**Visit**	**Planned day**	**From day**	**To day**	**Visit description**
2	1	1	1	Baseline*
	1	1	7	Day 1*
3	15	8	22	Day 15 (week 2)
4	29	23	36	Day 29 (week 4)
5	43	37	50	Day 43 (week 6)
6	57	51	71	Day 57 (week 8)
EOT	85	72	Date of drug intake in EOT visit+1	Day 85 (week 12)
FU	113	Date of drug intake in EOT visit+2	Last assessment date	Day 113 (FU)

*before drug admin its baseline, afterwards its Day 1

In addition to the visit window, a time window for planned time should be used for AOC3 as defined in the table below.

Table 6.7: 4 Time window for AOC3

	**Planned time**		**Interval definition (in minutes after treatment administration in that visit)**	
**Planned Day**	**hour**	**minute**	**from**	**to**
1, 29	-0.5	-30	-150	0
	0.5	30	1	45
	1	60	46	90
	2	120	91	150
	3	180	151	270
	6	360	271	420
	8	480	421	540
15, 43, 57	-0.5	-30	-150	0
85	-0.5	-30	-150	0
	0.5	30	1	45
	1	60	46	90
	2	120	91	150
	3	180	151	270
	6	360	271	420
	8	480	421	960
	24	1440	961	1620
113*	672	40320	Sample taken on follow up visit	

* For follow-up visit time from treatment administration in EOT visit is considered.

Proprietary confidential information © 2018 Boehringer Ingelheim International GmbH or one or more of its affiliated companies

Secondary biomarker endpoints:

Table 6.7: 5 Visit window for secondary and further biomarker endpoints*

		Interval Definition		
**Visit**	**Planned day**	**From day**	**To day**	**Visit description**
1	-28 to -7	NA	0	Screening
2	1	1	1	Baseline**
3	15	8	22	Day 15 (week 2)
4	29	23	36	Day 29 (week 4)
5	43	37	50	Day 43 (week 6)
6	57	51	71	Day 57 (week 8)
EOT	85	72	Date of drug intake in EOT visit +1	Day 85 (week 12)
FU	113	Date of drug intake in EOT visit +2	Last assessment date	Day 113 (FU)

*Measurements that are within some definition gaps will be assigned to the later window but will not be valid for the analysis of the window
**last value before drug admin is baseline, all previous measurements are called screening, when the measurement before first drug administration was done more than 8 weeks before first drug admin, this value will not be used as baseline.

For parameters which are not measured at each visit, still the defined windowing from Table 6.7:5 for the respective visit applies.

Safety endpoints including vital signs and lab parameters

Table 6.7: 6 Visit window for safety endpoints

		Interval Definition		
**Visit**	**Planned day**	**from**	**to**	**Visit description**
1	-28- -7	NA	0	Screening
2	1	1	1	Baseline*
3	15	2	22	Day 15 (week 2)
4	29	23	36	Day 29 (week 4)
5	43	37	50	Day 43 (week 6)
6	57	51	71	Day 57 (week 8)
EOT	85	72	Date of drug intake in EOT visit +14	Day 85 (week 12)
FU	113	Date of drug intake in EOT visit +15	Last assessment date	Day 113 (FU)

*last value before drug admin is baseline, all previous measurements are called screening, when the baseline measurement was done more than 8 weeks before first drug admin, this value will not be used anymore.

Proprietary confidential information © 2018 Boehringer Ingelheim International GmbH or one or more of its affiliated companies

Screening visits will only be shown in listings. If there is no measurement at the baseline visit, the screening measurement which is closest to the drug administration will be taken.

Repeated and unscheduled efficacy measurements will be listed in SDL according to visit window described below. Only one observation per time window will be selected for analysis at an on-treatment visit.

For efficacy measurements, the value will be selected which is closest to the protocol planned visit day.

For safety analysis except vital signs, worst value will be selected for analysis in case of multiple values within one visit window (see guidance for Handling, Display and Analysis of Laboratory Data (6)). For vital signs measurements, the value will be selected which is closest to the protocol planned visit day.

Proprietary confidential information © 2018 Boehringer Ingelheim International GmbH or one or more of its affiliated companies

7. PLANNED ANALYSIS

In general the display format of the analysis results follows BI guideline and standards as much as possible.

The following standard descriptive statistical parameters will be displayed in summary tables of continuous variables:

N	number of non-missing observations
Mean	arithmetic mean
SD	standard deviation
Min	minimum
Median	median
Max	maximum

Biomarker and safety lab values will show in addition Q1 (25th percentile) and Q3 (75th percentile).

For plasma concentrations as well as for all PK parameters, the following descriptive statistics will additionally be calculated:

CV	arithmetic coefficient of variation
gMean	geometric mean
gCV	geometric coefficient of variation
P10	10th percentile
Q1	25th percentile
Q3	75th percentile
P90	90th percentile

The data format for descriptive statistics of plasma concentrations will be identical with the data format of the respective concentrations. The descriptive statistics of PK parameters will be calculated using the individual values with the number of decimal places as provided by the evaluation program. Then the individual values as well as the descriptive statistics will be reported with three significant digits in the CTR.

For tables that are provided for endpoints with some extreme data, median, quartiles and percentiles should be preferred to mean, standard deviation, minimum and maximum.

Tabulations of frequencies for categorical data will include all possible categories and will display the number of observations in a category as well as the percentage (%) relative to the respective treatment group (unless otherwise specified, all patients in the respective patient set whether they have non-missing values or not).

The precision for percentages should be one decimal point, unless the denominator is smaller than 100 (in all treatment columns), in which case percentages are given in integer numbers. The category missing will be displayed only if there are actually missing values.

Proprietary confidential information © 2018 Boehringer Ingelheim International GmbH or one or more of its affiliated companies

The individual values of all patients will be listed, sorted by treatment group, centre (investigator site), patient number and visit, with ascending doses starting with placebo. The source data listings (SDL) will be contained in Appendix 16.2 of the CTR.

The tables and graphs will be contained in CTR in-text and Section 15.1-7 or in Appendix 16.1.9.13.

Analysis of biomarkers will be presented in 15.7 of the CTR and in Appendix 16.1.9.13.6

7.1 DEMOGRAPHIC AND OTHER BASELINE CHARACTERISTICS

Only descriptive statistics are planned for this section of the report. The evaluation of demographics and baseline characteristics will be based on the TS. The data will be summarized separately for each treatment and in total. Additionally the demographics and baseline biomarker parameters will be summarized for the defined subgroups in Section 6.4.

7.2 CONCOMITANT DISEASES AND MEDICATION

Only descriptive statistics are planned for this section of the report using the treated set.

Concomitant therapies (CTs) are coded according to WHO DD. CTs will be classified according to the Anatomical, Therapeutic, Chemical (ATC) classification system. The third ATC level will be used to categorise CTs by therapy type. In situations where a medical product may be used for more than one equally important indication, there are often several classification alternatives. As appropriate, patients receiving CTs with more than one possible ATC level-three category will be counted more than once; a footnote will clarify this possible double counting in tables. Summaries will be presented for new concomitant therapies added during randomised treatment phase and those taken at baseline. Therapies will be considered new if the added therapy is coded to a preferred name, where the patient did not report any medication that was coded to the same preferred name at baseline, except if the medication was stopped for more than one week.

Concomitant diseases are coded similarly as AEs based on the most current MedDRA® version. A summary of concomitant diseases will be provided by treatment group, system organ class (SOC), and preferred term (PT) and sorted alphabetically.

The coding version number will be displayed as a footnote in the respective output.

7.3 TREATMENT COMPLIANCE

Only descriptive statistics are planned for this section of the report. The number and percentage of patients with overall compliance will be reported. Overall compliance will be calculated as a weighted average of reported compliance. The sum of all reported compliance

Proprietary confidential information © 2018 Boehringer Ingelheim International GmbH or one or more of its affiliated companies

over the observed visits 2 to EOT visit will be multiplied by their duration and then divided by 100.

Example: 70% compliance between visit 2-3 (2 weeks)
90% compliance between visit 3-4 (2 weeks)
95% compliance between visit 4-5 (2 weeks)
100% compliance between visit 5-6 (2 weeks)
75% compliance between visit 6-EOT (4 weeks)
➔ Overall compliance = (70*2+90*2+95*2+100*2+75*4)/12= 84%

This patient would be overall compliant, but he didn’t reach 80% compliance within the last 4 weeks of treatment, so he will be excluded from PPS.

7.4 PRIMARY ENDPOINT

7.4.1 Primary analysis

The primary analysis of the primary endpoint will be performed as described in the CTP Section 7.3.1.
Relevant time points are:
predose (-0:30h relative to drug administration) at visit 2,3,4,5,6 and 24h at EOT. In case of missing 24h value at EOT, predose value planned -0.5h relative to drug administration can be used.

To account for heterogeneity between active and placebo treatment the power of mean variance estimates (POM) according to the paper (R17-1924) will be used.

Proprietary confidential information © 2018 Boehringer Ingelheim International GmbH or one or more of its affiliated companies

7.5 SECONDARY ENDPOINTS

7.5.1 Key secondary endpoint

Not applicable.

7.5.2 Secondary endpoints

<u>Primary analysis</u>

The number (%) patients with adverse reactions will be evaluated descriptively based on the TS. For more details see Section 7.8.1.

The primary analysis of the secondary biomarker endpoints defined in Section 5.2.2 is a MCPMod analysis with a previous applied MMRM model.

First a Mixed effects Model for Repeated Measurements (MMRM) over time is applied to the log transformed data (natural logarithms). The model will include the fixed effects 'base', 'treatment', 'time', 'base*time' interaction, and 'treatment*time' interaction. The covariance model for the repeated effect 'time' will be unstructured (i.e. TYPE=UN *or* UNR). For each dose group and each time point, the contrast of the means for ‘treatment-placebo’ will be estimated by the difference in the corresponding adjusted means (Least Squares Means); two-sided 90% CIs based on the t-distribution will also be computed. Baseline and all on-treatment time points will be used within the analysis. In case of convergence problem step 1-4 from Section 9.1.3 can be applied. The time profiles of the mean differences to placebo of the relative ALT (AST, AP, γ-GT, CK-18 caspase and CK-18 total respectively) change from baseline and the corresponding 90% CIs will be presented in a figure (back transformed on original scale using delta-delta method to calculate approximate SE and CIs). The dose-response relationship of the week 12 estimates from MMRM will then be analysed using MCPMod.
The Multiple Comparison Procedures and Modelling (MCP-Mod) approach (R10-1424, R15-4293) is implemented in two main steps: (1) trial design stage; (2) trial analysis stage. The procedures for the trial design stage, including the selection of candidate models covering a suitable range of dose-response shapes and sample size and power calculations, are provided in the CTP Section 7.3.2 and 7.7. The procedures for the trial analysis stage are specified below.

The treatment difference estimates over placebo for each active dose group, as well as their estimated variance-covariance matrix estimate from the MMRM are used in the trial analysis stage. Multiple comparison procedure will be implemented using optimal contrast tests which control the family-wise type I error rate at one-sided $\alpha = 0.05$. The optimal contrasts

Proprietary confidential information © 2018 Boehringer Ingelheim International GmbH or one or more of its affiliated companies

corresponding to the candidate models are calculated as in the trial design stage and are shown in Table 7.5.2: 1. They will be updated using the expected model means from candidate set and the estimated variance-covariance matrix from the data.

Table 7.5.2: 1 Contrast coefficients

	Contrast coefficients for dose				
Model	Dose 0 mg	Dose 1 mg	Dose 3 mg	Dose 6 mg	Dose 10 mg
Linear	0.492	0.369	0.123	-0.246	-0.739
Linear in log	0.797	0.141	-0.143	-0.328	-0.466
Quadratic	0.598	0.377	0.003	-0.388	-0.591
Exponential	0.387	0.336	0.204	-0.099	-0.828
Emax	0.831	0.094	-0.210	-0.329	-0.385
Sigmoid Emax	0.462	0.453	0.135	-0.453	-0.597
Logistic	0.468	0.418	0.168	-0.422	-0.632
Beta model	0.449	0.280	-0.452	-0.627	0.350

If at least one dose-response model is statistically significant, rejecting the null hypothesis of a flat dose-response curve is indicating a benefit of BI 1467335 over placebo.

When the null hypothesis is rechecked, the best-fitting model from the above set of eight models can be refitted to the data without any parameter assumptions to generate new estimates of the model parameters from the data. The target dose will be obtained via model averaging across the significant models based on Akaike Information Criterion (AIC) (the smaller the AIC value the better the model fit).

Proprietary confidential information © 2018 Boehringer Ingelheim International GmbH or one or more of its affiliated companies

Proprietary confidential information © 2018 Boehringer Ingelheim International GmbH or one or more of its affiliated companies

Proprietary confidential information © 2018 Boehringer Ingelheim International GmbH or one or more of its affiliated companies

Proprietary confidential information © 2018 Boehringer Ingelheim International GmbH or one or more of its affiliated companies

Proprietary confidential information © 2018 Boehringer Ingelheim International GmbH or one or more of its affiliated companies

7.7 EXTENT OF EXPOSURE

Basis for the assessment of the treatment exposure will be the amount of trial medication intake and the duration of exposure counted in days. Standard statistical parameters will be displayed by treatment.

7.8 SAFETY ANALYSIS

All safety analyses will be performed on the treated set.

7.8.1 Adverse events

Unless otherwise specified, the analyses of AEs will be descriptive in nature. All analyses of AEs will be based on the number of patients with AEs and NOT on the number of AEs. The reporting and analyses of AEs will follow the BI guideline (4).

Proprietary confidential information © 2018 Boehringer Ingelheim International GmbH or one or more of its affiliated companies

For analysis multiple AE occurrence data on the CRF will be collapsed into an AE provided that all of the following applies:

- All AE attributes are identical (LLT, intensity according to CTCAE Version 4.03, action taken, therapy required, seriousness, reason for seriousness, relationship, outcome, AE of special interest).
- The occurrences were time-overlapping or time-adjacent (time-adjacency of 2 occurrences is given if the second occurrence started on the same day or on the day after the end of the first occurrence)

For further details on summarization of AE data, please refer to guidelines (3, 4).
The analysis of adverse events will be based on the concept of treatment emergent adverse events. That means that all adverse events occurring between first drug intake until trial termination date will be assigned to the treatment they were randomised to. All adverse events occurring before first drug intake will be assigned to 'screening'. For details on the treatment definition, see Section 6.1.

According to ICH E3 (5), AEs classified as 'other significant' needs to be reported and will include those non-serious and non-significant adverse events with
(i) 'action taken = discontinuation' or 'action taken = reduced', or
(ii) marked haematological and other lab abnormalities or lead to significant concomitant therapy as identified by the Clinical Monitor/Investigator at a Medical Quality Review Meeting.

An overall summary of adverse events for BI 1467335 will be presented. For further details on which summaries will be provided see Section 6.1.

The frequency of patients with adverse events will be summarised by treatment, primary system organ class and preferred term. Separate tables will be provided for patients with other significant adverse events according to ICH E3 (5), for patients with adverse events of special interest and for patients with serious adverse events.

The system organ classes will be sorted alphabetically; preferred terms will be sorted alphabetically as well (within system organ class).

7.8.2 Laboratory data

The analyses of safety laboratory data will be descriptive in nature and will be based on BI standards (6).

For continuous safety laboratory parameters standardised and normalised values will be derived as well as the differences to baseline. The process of standardisation and normalisation as well as standard analyses for safety laboratory data are described in the BI guidance for the Display and Analysis of Laboratory Data (6).

Laboratory values will be compared to their reference ranges and frequency tables will be provided for the number of patients within and outside the reference range at baseline and with 12 weeks of treatment and with the follow up visit 4 weeks after last drug administration. Descriptive statistics will be provided by treatment group for baseline, on-

Proprietary confidential information © 2018 Boehringer Ingelheim International GmbH or one or more of its affiliated companies

treatment values and for changes from baseline. Frequency tables will summarise the number of patients with potentially clinically significant abnormalities as defined for the new XLAB macro.

Clinically relevant findings in laboratory data will be reported as AEs and will be analysed as part of AE analysis.

The Estimated Glomerular filtration rate as assessed by the CKD-EPI formula:

$$\text{eGFR (ml/min/1.73 m}^2\text{)} = 141 * \min(S_{Cr}/\kappa, 1)^{\alpha} * \max(S_{Cr}/\kappa, 1)^{-1.209} * 0.993^{Age} * 1.018 \text{ [if female]} * 1.159 \text{ [if black]}$$

where S_{Cr} is serum creatinine in mg/dL, κ is 0.7 for females and 0.9 for males, α is -0.329 for females and -0.411 for males, min indicates the minimum of S_{Cr}/κ or 1, and max indicates the maximum of S_{Cr}/κ or 1. The process of standardisation and normalisation as described in the guidance document (6) does not apply. Additionally the shift tables for eGFR will use the following categories (similar to the staging of renal impairment): eGFR ≥ 90; 60-<90; 30-<60; and<30.

7.8.3 Vital signs

Descriptive statistics of absolute values, changes from baseline and compared to placebo of the exploratory vital signs endpoints defined in Section 5.3.2 over time will be provided.

Clinically relevant findings in vital signs data will be reported as AEs and will be analysed as part of AE analysis.

7.8.4 ECG

All evaluation of ECG data except of exposure response analysis will be based on the ECGS. The exposure-response analysis will then be done on the ECGPCS.

<u>Listing of individual data</u>

For all quantitative endpoints, listings of individual data will be shown in Appendix 16.2. Occurrences of notable findings will be flagged.

For all patients with any notable finding in quantitative ECG recordings, a separate listing will be created as end-of-text display (based on the same display template as in Appendix 16.2), and the corresponding time profiles will be shown.

Comments regarding the ECGs will be listed.

<u>Analyses categorical Endpoints</u>

For the categorical endpoints, frequency tables will be provided.

Proprietary confidential information © 2018 Boehringer Ingelheim International GmbH or one or more of its affiliated companies

The findings (ECG abnormalities) resulting from morphological analyses of the ECGs will also analysed as categorical endpoints.

Analyses of quantitative endpoints

Descriptive statistics (N, mean, SD, min, median, max) will be provided for the absolute values as well as the changes from baseline over time for QTcF, HR, QT, PR and QRS. Time profiles of mean and SD for the changes from baseline on treatment will be displayed graphically by treatment.

For QTcF and HR changes from baseline the relationship to the corresponding plasma concentration is evaluated using an exposure response model.

For the following analyses, all time points with available ECG endpoints and corresponding plasma concentrations will be included. For patients on active drug, in case of BLQ values they will be replaced by ½ LLOQ for analysis.

The changes from baseline in QTcF (ΔQTcF) can be investigated as response variable. The placebo patients in the analysis will be included, with zero plasma concentrations.

As a first step, it is investigated if there is a potential delayed or accelerated (e.g. due to metabolites) effect of the drug on QTcF. A general visual impression will be provided by overlaying time profiles of plasma concentrations and QTcF changes from baseline (ΔQTcF).All figures will be generated for each patient (presented in Appendix 16.1.13.1.9.1 of the CTR), as well as for means per active treatment (presented in Section 15.3 of the CTR).

The relationship between BI 1467335 plasma concentrations and QTcF changes from baseline will be investigated in an exploratory manner using a random coefficient model to estimate the difference in means between BI 1467335 and placebo of QTcF change from baseline and its 90% confidence interval at the geometric mean of C_{max} for each dose. Additionally, the estimated overall slope with its 90% confidence interval will be provided. The used random coefficient model is based on a white paper from Garnett et. al. (R18-0143) with ΔQTcF as response variable, centered baseline QTc and plasma concentration as continuous covariates, treatment and day as fixed categorical effects, and a random intercept and slope for each patient. For more details refer to Section 9.1.2.

For visualization, a scatterplot of the BI 1467335 plasma concentration against the following individual QTcF values will be provided: For each patient on active treatment and each time point, subtract the mean value of all individual observed ΔQTcF values from the placebo group for this time point from the individual observed ΔQTcF value for this patient and time point. This results in estimates for "individual ΔΔQTcF" values, which should only be used for plotting purposes. The corresponding regression line and its pointwise confidence bands as well as and the geometric mean of C_{max} for each dose will additionally be displayed in the plot.

To check model assumptions, the conditional residuals will be plotted and presented in Appendix 16.1.13.1.9.1 of the CTR. In case of non-linearity or if there is evidence for a

Proprietary confidential information © 2018 Boehringer Ingelheim International GmbH or one or more of its affiliated companies

delayed effect, further models will be explored in order to better characterise the PK-ECG relationship (e.g. effect compartment models, non-linear models, etc.).

All of the above described graphical and statistical analyses will be also performed for HR in place of QTcF.

Appropriateness of heart rate correction methods of QT interval

To evaluate the appropriateness of the heart rate correction methods, the slope of the relationship of QTcF interval versus RR interval (values log-transformed using the natural logarithm) will be estimated by applying the random coefficient model described in Section 9.1.1 using all time points. A scatterplot of QTcF vs RR including the overall regression line will be included in the Statistical Appendix of the CTR. The resulting (fixed effect) slope together with two-sided 95% confidence intervals will be included in the footnote for this plot.

Proprietary confidential information © 2018 Boehringer Ingelheim International GmbH or one or more of its affiliated companies

8. REFERENCES

1.	*CPMP/ICH/363/96:* "Statistical Principles for Clinical Trials", ICH Guideline Topic E9, Note For Guidance on Statistical Principles for Clinical Trials, current version.
5.	*CPMP/ICH/137/95*: "Structure and Content of Clinical Study Reports", ICH Guideline Topic E3; Note For Guidance on Structure and Content of Clinical Study Reports, current version
7.	*R18-0143:* Garnett C, Bonate PL, Dang Q, Ferber G, Huang D, Liu J, et al; Scientific white paper on concentration-QTc modeling. J Pharmacokin Pharmacodyn (2017)
8.	*R05-0788*: Hoffman D, Kringle R, Lockwood G, Turpault S, Yow E, Mathieu G. Nonlinear mixed effects modelling for estimation of steady state attainment. Pharm Stat. 2005.
9.	*R17-1924:* Giltinan, D.M. & Ruppert, D. Fitting Heteroscedastic Regression Models to Individual Pharmacokinetic Data Using Standard Statistical Software. Journal of Pharmacokinetics and Biopharmaceutics (1989) 17: 601.
10.	*R10-1424*: Pinheiro J, Bornkamp B, Bretz F; Design and analysis of dose-finding studies combining multiple comparisons and modeling procedures.; J Biopharm Stat 16 (5), 639 - 656 (2006)
11.	*R15-4293:* Pinheiro J, Bornkamp B, Glimm E, Bretz F; Model-based dose finding under model uncertainty using general parametric models.; Stat Med 33 (10), 1646 - 1661 (2014)
12.	*R17-3207:* P. Angulo, Jason M, G Machesini, et al. The NAFLD Fibrosis Score: A Noninvasive System That Identifies Liver Fibrosis in Patients with NAFLD. Hepatology 2007; 45;846-854
13.	*R17-3206:* Sterling RK, Lissen E, Clumeck N, et. al. Development of a simple noninvasive index to predict significant fibrosis patients with HIV/HCV co-infection. Hepatology 2006;43:1317-1325.
14.	*R17-3205:* Lin ZH, Xin YN, Dong QJ, et al. Performance of the aspartate aminotransferase-to-platelet ratio index for the staging of hepatitis C-related fibrosis: an updated meta-analysis. Hepatology. 2011;53:726-36.

Proprietary confidential information © 2018 Boehringer Ingelheim International GmbH or one or more of its affiliated companies

17.	*R10-2920:* Ring A; Statistical models for heart rate correction of the QT interval; Stat Med 29, 786-796 (2010)
18.	*R15-2001:* Bornkamp B; Pinheiro J; Bretz F, Package 'DoseFinding' (February 19, 2015). http://cran.r-project.org/web/packages/DoseFinding/DoseFinding.pdf (access date: 28 April 2015) ; Comprehensive R Archive Network; 2015.

Proprietary confidential information © 2018 Boehringer Ingelheim International GmbH or one or more of its affiliated companies

Proprietary confidential information © 2018 Boehringer Ingelheim International GmbH or one or more of its affiliated companies

Proprietary confidential information © 2018 Boehringer Ingelheim International GmbH or one or more of its affiliated companies

Proprietary confidential information © 2018 Boehringer Ingelheim International GmbH or one or more of its affiliated companies

Proprietary confidential information © 2018 Boehringer Ingelheim International GmbH or one or more of its affiliated companies

Proprietary confidential information © 2018 Boehringer Ingelheim International GmbH or one or more of its affiliated companies

10. HISTORY TABLE

Table 10: 1 History table

**Version**	**Date (DD-MMM-YY)**	**Author**	**Sections changed**	**Brief description of change**
Initial	**03-APR-2017**		None	This is the initial TSAP with necessary information for trial conduct
FINAL	**10-AUG-2017**		All	This is the final TSAP version. All missing information from initial TSAP version was added.
