# Supplementary material for: A randomised Phase IIa trial of amine oxidase copper-containing 3 (AOC3) inhibitor BI 1467335 in adults with non-alcoholic steatohepatitis
Source: Nat Commun. 2023 Nov 6;14:7151. doi: 10.1038/s41467-023-42398-w (PMC10628239; doi:10.1038/s41467-023-42398-w)
Supplement: Supplementary file 3 — Reporting Summary [file 41467_2023_42398_MOESM3_ESM.pdf]

nature portfolio

Corresponding author(s): DBPR#NCOMMS-22-47514-T

Last updated by author(s): Jul 11, 2023

Reporting Summary

Nature Portfolio wishes to improve the reproducibility of the work that we publish. This form provides structure for consistency and transparency in reporting. For further information on Nature Portfolio policies, see our Editorial Policies and the Editorial Policy Checklist.

Statistics

For all statistical analyses, confirm that the following items are present in the figure legend, table legend, main text, or Methods section.

n/a	Confirmed	
☐	☒	The exact sample size (*n*) for each experimental group/condition, given as a discrete number and unit of measurement
☐	☒	A statement on whether measurements were taken from distinct samples or whether the same sample was measured repeatedly
☐	☒	The statistical test(s) used AND whether they are one- or two-sided<br>*Only common tests should be described solely by name; describe more complex techniques in the Methods section.*
☐	☒	A description of all covariates tested
☐	☒	A description of any assumptions or corrections, such as tests of normality and adjustment for multiple comparisons
☐	☒	A full description of the statistical parameters including central tendency (e.g. means) or other basic estimates (e.g. regression coefficient) AND variation (e.g. standard deviation) or associated estimates of uncertainty (e.g. confidence intervals)
☐	☒	For null hypothesis testing, the test statistic (e.g. *F*, *t*, *r*) with confidence intervals, effect sizes, degrees of freedom and *P* value noted<br>*Give P values as exact values whenever suitable.*
☒	☐	For Bayesian analysis, information on the choice of priors and Markov chain Monte Carlo settings
☒	☐	For hierarchical and complex designs, identification of the appropriate level for tests and full reporting of outcomes
☒	☐	Estimates of effect sizes (e.g. Cohen's *d*, Pearson's *r*), indicating how they were calculated

Our web collection on statistics for biologists contains articles on many of the points above.

Software and code

Policy information about availability of computer code

Data collection	SAS version 9.4 (SAS Inc., Cary, NC, USA)
Data analysis	SAS version 9.4 (SAS Inc., Cary, NC, USA)

For manuscripts utilizing custom algorithms or software that are central to the research but not yet described in published literature, software must be made available to editors and reviewers. We strongly encourage code deposition in a community repository (e.g. GitHub). See the Nature Portfolio guidelines for submitting code & software for further information.

Data

Policy information about availability of data

All manuscripts must include a data availability statement. This statement should provide the following information, where applicable:

- Accession codes, unique identifiers, or web links for publicly available datasets
- A description of any restrictions on data availability
- For clinical datasets or third party data, please ensure that the statement adheres to our policy

To ensure independent interpretation of clinical study results and enable authors to fulfil their role and obligations under the ICMJE criteria, Boehringer Ingelheim grants all external authors access to relevant clinical study data. In adherence with the Boehringer Ingelheim Policy on Transparency and Publication of Clinical Study Data, scientific and medical researchers can request access to clinical study data after publication of the primary manuscript and secondary analyses in peer-reviewed journals and regulatory and reimbursement activities are completed, normally within 1 year after the marketing application has been granted by major Regulatory Authorities. Researchers should use the https://vivli.org/ link to request access to study data and visit https://www.mystudywindow.com/msw/

datasharing for further information.

Human research participants

Policy information about studies involving human research participants and Sex and Gender in Research.

Reporting on sex and gender	Demographic information, including patient sex, were collected at the Screening visit; both male and female patients were eligible for the clinical trial. Of the 113 participants treated with BI 1467335 or placebo in this study, 58 (51.3%) were female (BI 1467335: 1 mg, n=10, 62.5%; 3 mg, n=8, 50.0%; 6 mg, n=9, 52.9%; 10 mg, n=18, 56.3%; placebo, n=13, 40.6%) and 55 (48.7%) were male (BI 1467335: 1 mg, n=6, 37.5%; 3 mg, n=8, 50.0%; 6 mg, n=8, 47.1%; 10 mg, n=14, 43.8%; placebo, n=19, 59.4%). Information regarding patient gender was not collected in this trial. Patient characteristics and demographics were similar between groups, and no subgroup analyses were conducted according to sex.
Population characteristics	Eligible patients were adults (18–75 years), with either clinical evidence of NASH defined as histological evidence no more than 3 years prior to screening or clinical imaging suggestive of NASH (evidence of hepatic steatosis by magnetic resonance imaging of proton density fat fraction or ultrasound and evidence of liver fibrosis defined by stiffness >3.64 kPa with magnetic resonance elastography or >7.2 kPa with transient elastography) no more than 3 years prior to screening or within the screening phase. Patients were also required to have an ALT level >1.5 to ≤5.0 × the upper limit of normal (ULN) or historic ALT >1.25 × ULN within 1 week to 3 months prior to screening and two consecutive ALT >1.5 × ULN measurements at least one week apart during the screening period to be included in the trial. Among the 113 patients who received BI1467335 or placebo, approximately half were male (n=55; 48.7%), most patients (n=109; 96.5%) were white and the mean age (standard deviation) was 51.1 years (12.5).
Recruitment	In total, 108 patients with clinical evidence of NASH were planned to be randomised to 1 of the 5 treatment arms. Approximately 50 sites in various countries were planned to participate. Recruitment was competitive. The patients who discontinued the trial following randomisation were not replaced and could not be re-enrolled at a later date. The reasons for early discontinuation were recorded. The permission to randomise more than 20 patients per site had to be obtained from the CTL at BI. This was only allowed after a careful review of the enrolment status.<br>The primary analysis findings were confirmed by sensitivity analyses performed with different methods of imputation and/or a different population. Further analysis of biomarker endpoints showed some differences between treatment groups at some time points, which are likely to be due to the large variability between patients, the presence of outliers and the small sample size in each group.
Ethics oversight	All patients provided written informed consent before entering the trial and this trial was conducted in accordance with Good Clinical Practice, the ethical principles laid down in the Declaration of Helsinki and applicable regulatory requirements. The full study protocol (version 3.0, 19 May 2017 and subsequent amendments) was approved by the Research Ethics Committee of the Coordinating Investigator of the trial (Prof. Philip Newsome), University Hospitals Birmingham NHS Foundation Trust and University of Birmingham, Birmingham, UK, and is available as a supplementary file.

Note that full information on the approval of the study protocol must also be provided in the manuscript.

Field-specific reporting

Please select the one below that is the best fit for your research. If you are not sure, read the appropriate sections before making your selection.

- [x] Life sciences
- [ ] Behavioural & social sciences
- [ ] Ecological, evolutionary & environmental sciences

For a reference copy of the document with all sections, see nature.com/documents/nr-reporting-summary-flat.pdf

Life sciences study design

All studies must disclose on these points even when the disclosure is negative.

Sample size	The sample size calculation was based on the primary endpoint, as well as the ALT change from baseline; the latter being the biomarker with the least favourable ratio of anticipated effect size to variance. A sample size of 108 patients with an allocation ratio of 2:1:1:1:2 for placebo, BI 1467335 1 mg, 3 mg, 6 mg or 10 mg, with an assumed 10% discontinuation rate and assumed maximum change of 30%, was anticipated to provide sufficient precision for the primary endpoint evaluation as well as a 84.8% probability of detecting a 20% relative change from baseline in ALT at Week 12. This calculation was based on 1000 simulations using Multiple Comparison Procedure – Modelling (MCPMod),35 assuming a baseline ALT of 80 U/L with an SD of 40, a placebo effect of −10 U/L and a treatment effect of −34 U/L at Week 12, with the null hypothesis of no dose relationship rejected at one-sided alpha 0.05.
Data exclusions	The treated population included 113 patients who received at least one dose of daily oral BI 1467335 1 mg (n=16), 3 mg (n=16), 6 mg (n=17), 10 mg (n=32) or placebo (n=32). In total, 16 patients had at least one important protocol deviation (treatment duration that was too short [n=11, preventative termination following consumption of tyramine-rich foods/tryptophan supplements]; n=5, failure to meet entry criteria; n=4, non-compliance to trial medication; n=2, prohibited medication use; n=2, missing on-treatment biomarker value; n=1, no trial medication taken), meaning the per protocol population included 98 patients. The safety population included 113 patients. The full analysis set, which was used for the sensitivity analysis of the primary endpoint, included 112 patients (one patient was excluded from the FAS due to the absence of on-treatment biomarker value for all of AOC3 activity, ALT, AST, AP, GGT, CK-18 caspase and CK-18 total).

Replication	Further development of BI 1467335 was stopped due to the risk of drug interactions of the compound with monoamine oxidase (MAO)-B in NASH patients identified in another Phase I trial. Replication or confirmation of these findings in further trials is therefore not anticipated.
Randomization	Patients were randomised using interactive response technology (IRT) 2:1:1:1:2 (block size 7) to receive placebo or BI 1467335 1 mg, 3 mg, 6 mg or 10 mg orally once daily for 12 weeks. Use of IRT provided a depersonalised patient identification code and ensured confidentiality of patient data; the randomisation list was generated using a validated system, which involved a pseudo-random number generator so that the resulting treatment was both reproducible and non-predictable.
Blinding	All trial participants, investigators and site staff were blinded to the assigned treatment.

Reporting for specific materials, systems and methods

We require information from authors about some types of materials, experimental systems and methods used in many studies. Here, indicate whether each material, system or method listed is relevant to your study. If you are not sure if a list item applies to your research, read the appropriate section before selecting a response.

Materials & experimental systems

n/a	Involved in the study
☒	☐ Antibodies
☒	☐ Eukaryotic cell lines
☒	☐ Palaeontology and archaeology
☒	☐ Animals and other organisms
☐	☒ Clinical data
☒	☐ Dual use research of concern

Methods

n/a	Involved in the study
☒	☐ ChIP-seq
☒	☐ Flow cytometry
☒	☐ MRI-based neuroimaging

Clinical data

Policy information about clinical studies

All manuscripts should comply with the ICMJEguidelines for publication of clinical research and a completedCONSORT checklist must be included with all submissions.

Clinical trial registration	ClinicalTrials.gov: NCT03166735
Study protocol	The full study protocol is available as a supplementary file.
Data collection	This multicentre, parallel-group, randomised, double-blind, placebo-controlled Phase IIa trial was conducted at 44 centres across the US, Germany, Spain, Belgium, the UK, Netherlands, Canada, France and Ireland. Between 27 July 2017 and 14 June 2019, 114 patients were enrolled and randomly assigned to one of five treatment groups.<br>Assessments of sAOC3 activity and concentration, ALT, AST, AP, GGT, CK-18 caspase and CK-18 total, and other exploratory biomarkers were carried out at each study visit. Study visits took place every 2 weeks for the first 8 weeks, at Week 12 and at a follow-up visit 4 weeks after trial drug termination (Week 16). TEAEs were recorded at each study visit, at follow-up and by telephone call on Days 27 and 83.
Outcomes	The primary endpoint was plasma AOC3 activity relative to baseline measured as a percentage, 24 hours post dose after 12 weeks of treatment. AOC3 activity was measured via a quasi-quantitative, one-step fluorometric activity assay (Amplex® Red Monoamine Oxidase Assay Kit). In this assay, hydrogen peroxide, produced during oxidation of benzylamine by AOC3, was used as a proxy for quantification of AOC3 activity. Hydrogen peroxide oxidized Amplex Red to its fluorescent analogue, resorufin, allowing for colorimetric analysis of AOC3 activity. The number and percentage of patients with drug-related adverse events (AEs) was a secondary endpoint in this trial. Safety and tolerability were further assessed based on the general occurrence of treatment emergent AEs, safety laboratory parameters, physical examination, vital sign measurements and a 12-lead electrocardiogram. The intensity of AEs was classified and recorded according to the Common Terminology Criteria for Adverse Events (CTCAE) v4.03. Secondary biomarker endpoints were relative changes from baseline in ALT, AST, AP, GGT, CK-18 caspase, and CK-18 total at Week 12. Further biomarker endpoints included markers of metabolism (e.g. fasting plasma glucose, insulin and lipids), inflammation (e.g. interleukins 1β, 6 and 8, and interferon γ) and markers and scores reflecting fibrosis status (e.g. APRI, Fib-4 score, ELF score, NAFLD fibrosis score and Pro-C3).<br>Statistical analyses were performed using SAS version 9.4 (SAS Inc., Cary, NC, USA). Primary and secondary non-safety endpoints were evaluated using the per-protocol population of all randomized patients, excluding patients with no baseline and/or on-treatment value and important protocol deviations leading to exclusion. Safety analyses used all randomized patients who received trial treatment. The dose–response relationship of the primary endpoint was analyzed using a nonlinear regression model (decreasing Emax curve) applied to the AOC3 activity at Week 12. The fitted regression model was used to derive the smallest dose where the mean plasma AOC3 activity curve dropped below 10%. For the secondary biomarker endpoints, the MMRM was used to generate adjusted mean and 90% CI estimates for the treatment effects at Week 12. These estimates, together with the corresponding covariance matrix, were used to analyze the dose–response relationship by examining the fit of eight shapes modeled using MCPMod36,37, allowing for simultaneous evaluation of different potential dose–response patterns, while protecting the overall probability of type I error (one-sided alpha of 0.05). A test for non-flat dose–response relationship was first performed; if a relationship could be shown, all significant models from a set of candidate models were selected and fitted to the data as a second step.
