# Supplementary material for: A randomised Phase IIa trial of amine oxidase copper-containing 3 (AOC3) inhibitor BI 1467335 in adults with non-alcoholic steatohepatitis
Source: Nat Commun. 2023 Nov 6;14:7151. doi: 10.1038/s41467-023-42398-w (PMC10628239; doi:10.1038/s41467-023-42398-w)
Supplement: Supplementary file 2 — Peer Review File [file 41467_2023_42398_MOESM2_ESM.pdf]

nature portfolio

Peer Review File

A randomised Phase IIa trial of amine oxidase copper-containing 3 (AOC3) inhibitor BI 1467335 in adults with non-alcoholic steatohepatitis

Open Access This file is licensed under a Creative Commons Attribution 4.0 International License, which permits use, sharing, adaptation, distribution and reproduction in any medium or format, as long as you give appropriate credit to the original author(s) and the source, provide a link to the Creative Commons license, and indicate if changes were made. In the cases where the authors are anonymous, such as is the case for the reports of anonymous peer reviewers, author attribution should be to 'Anonymous Referee' followed by a clear attribution to the source work. The images or other third party material in this file are included in the article's Creative Commons license, unless indicated otherwise in a credit line to the material. If material is not included in the article's Creative Commons license and your intended use is not permitted by statutory regulation or exceeds the permitted use, you will need to obtain permission directly from the copyright holder. To view a copy of this license, visit http://creativecommons.org/licenses/by/4.0/.

Editorial Note: This manuscript has been previously reviewed at another journal that is not operating a transparent peer review scheme. This document only contains reviewer comments and rebuttal letters for versions considered at *Nature Communications*.

REVIEWERS' COMMENTS

Reviewer #1 (Remarks to the Author):

The authors have dealt with the issues raised by previous reviewers.

In addition, I note the additional clarity over the sample size, randomisation and presentation of results which are welcome and conform with the included SAP.

Reviewer #2 (Remarks to the Author):

Minor comments:

1. Lines 209 to 224 in the discussion section are best suited for the introduction section. You may condense lines 69 to 87 to accommodate the additional text.
2. The primary outcome is not reported in the format mentioned in the protocol anywhere in the text - primary endpoint is the plasma amine oxidase copper-containing 3 (AOC3) activity
relative to baseline in %, 24 h post dose, after 12 weeks of treatment. Just mentioning that >80% reduction is achieved is not granular enough. Furthermore, when i look at Table S1 in the 10 mg cohort the baseline value of sAOC3 was 526 ± 137 ug/L and week 12 was 499 ± 143 ug/L and this resulted in >80% decrease in activity? Is there no correlation between serum levels and activity? Can you include the methodology for measurement of AOC activity or at least include a reference for it? The authors need to examine the relationship between the serum levels and activity and help the readers understand this relationship better.

Reviewer #3 (Remarks to the Author):

All concerns have adequately been adressed

NCOMMS-22-47514-T – A randomised phase IIa trial of amine oxidase copper-containing 3 (AOC3) inhibitor BI 1467335 in adults with non-alcoholic steatohepatitis

Corresponding author: Philip Newsome

Note: Page and line references given within the response section refer to the marked-up version of the re-submitted manuscript.

Reviewer 1

Remarks to the author:

The authors have dealt with the issues raised by previous reviewers.

In addition, I note the additional clarity over the sample size, randomisation and presentation of results which are welcome and conform with the included SAP.

Many thanks for the positive feedback; this is greatly appreciated.

Reviewer 2

Remarks to the author:

Minor comments

Comments:

1. Lines 209 to 224 in the discussion section are best suited for the introduction section. You may condense lines 69 to 87 to accommodate the additional text.
   Thank you for your guidance, the paragraph you have identified in the Discussion section has now been moved to the Introduction section, and the existing text has been shortened and adapted accordingly to accommodate this additional text (pages 3–4: lines 55–57; 63–65; 72–83; 88–89).

2. The primary outcome is not reported in the format mentioned in the protocol anywhere in the text - primary endpoint is the plasma amine oxidase copper-containing 3 (AOC3) activity relative to baseline in %, 24 h post dose, after 12 weeks of treatment. Just mentioning that >80% reduction is achieved is not granular enough. Furthermore, when i look at Table S1 in the 10 mg cohort the baseline value of sAOC3 was 526 ± 137 ug/L and week 12 was 499 ± 143 ug/L and this resulted in >80% decrease in activity? Is there no correlation between serum levels and activity? Can you include the methodology for measurement of AOC activity or at least include a reference for it? The authors need to examine the relationship between the serum levels and activity and help the readers understand this relationship better.
   Thank you for your comments, the descriptions of the primary endpoint found in the Abstract (page 2, lines 33 and 34) and in the Methods section (outcomes, page 12, lines 304–305) have now been amended to more closely reflect the definition provided in the protocol.

   Abstract: "Primary endpoint was AOC3 activity relative to baseline (%), 24 hours post dose after 12 weeks' treatment."

   Methods: "The primary endpoint was plasma AOC3 activity relative to baseline measured as a percentage, 24 hours post dose after 12 weeks of treatment."

To provide greater granularity, we have added a new Supplementary Table (Supplementary Table 1) showing plasma AOC3 activity relative to baseline (%) at Weeks 2, 4, 6, 8, 12 and 16 (Supplementary materials, page 7, lines 132–135). We have also detailed the corresponding AOC3 activity data for the primary endpoint (Week 12 data) in the Results section for the placebo group and each of the BI 1467335 dose groups (Primary outcome, page 5-6, lines 117–122). To assist with understanding the statements regarding >80% reduction/inhibition of activity, we also convert the AOC3 activity data to % inhibition, i.e. 100% − % activity. These changes are also mirrored in the Abstract (page 2, lines 34–36) for consistency.

*Abstract: "*Mean AOC3 activities relative to baseline at Week 12: no change (placebo), 26.5% (1 mg), 10.4% (3 mg), 5.0% (6 mg), 3% (10 mg). These changes indicated that BI 1467335 dose dependently inhibited AOC3 activity; ≥3 mg doses achieved >80% inhibition (<20% activity) at Week 4."

*Results: "*BI 1467335 resulted in dose-dependent inhibition of plasma AOC3 activity, with >80% reductions from baseline seen by Week 4 with doses of ≥3 mg. Relative to baseline values, mean AOC3 activity at Week 12 (24 hours post dose) was 10.4% in the 3 mg group (indicating 89.6% inhibition), 5.0% in the 6 mg group (95.0% inhibition) and 3.3% in the 10 mg group (96.7% inhibition); plasma AOC3 activity was 26.5% in the BI 1467335 1 mg dose group, (indicating 73.5% inhibition) and no change was observed in patients receiving placebo, thus meeting the primary endpoint (Supplementary Table 1)."

Table S2 shows AOC3 concentration, which fluctuated but remained similar to baseline levels throughout the trial in the placebo and BI 1467335 groups, whereas Figure 2 shows the change from baseline in AOC3 activity. We have now clarified this in the Results section by specifically stating that these data show the ability of BI 1467335 to inhibit plasma AOC3 activity *without* affecting the concentration of AOC3 in the plasma (Primary outcome, page 5, lines 129–132).

"Plasma AOC3 concentrations fluctuated but remained similar to baseline levels throughout the trial in the placebo and across all BI 1467335 groups (Table S2). Therefore, BI 1467335 inhibited AOC3 activity without altering the plasma concentration of AOC3."

As requested, methodology for measurement of AOC activity has now been added to the Methods section (Outcomes, page 12, lines 305–309).

"*AOC3 activity was measured via a quasi-quantitative, one-step fluorometric activity assay (Amplex® Red Monoamine Oxidase Assay Kit)*. In this assay, hydrogen peroxide, produced during oxidation of benzylamine by AOC3, was used as a proxy for quantification of AOC3 activity. Hydrogen peroxide oxidised Amplex Red to its fluorescent analogue, resorufin, allowing for colorimetric analysis of AOC3 activity."

Reviewer 3
Remarks to the Author:
All concerns have adequately been addressed

Many thanks for the positive feedback; this is greatly appreciated.
